# Supplementary material for: BioSANS: A software package for symbolic and numeric biological simulation
Source: PLoS One. 2022 Apr 18;17(4):e0256409. doi: 10.1371/journal.pone.0256409 (PMC9015124; doi:10.1371/journal.pone.0256409)
Supplement: S1 File — (PDF) [file pone.0256409.s001.pdf]

Supplementary Information for
BioSANS: A Software Package for Symbolic and Numeric Biological Simulation

Erickson Fajiculay[1,2,3], Chao-Ping Hsu[1,4,5*]

1. Institute of Chemistry, Academia Sinica, Taipei, 11529, Taiwan
2. Bioinformatics Program, Institute of Information Science, Taiwan International Graduate Program, Academia Sinica, Taipei, 11529, Taiwan
3. Institute of Bioinformatics and Structure Biology, National Tsinghua University, 30044, Taiwan
4. Physics Division, National Center for Theoretical Sciences, Taipei, 10617, Taiwan
5. Genome and Systems Biology Degree program, National Taiwan University, Taipei, 10617, Taiwan

* CorrespondingAuthor
Email : cherri@sinica.edu.tw

In this supplementary document, we include the user manual for BioSANS, including the guides for installation and usage, and considerations in the methodology included in the program.

2021

BioSANS Manual/Tutorials

MODEL CREATION, PROPAGATION & ANALYSIS

ERICKSON FAJICULAY

Table of contents

1 BioSANS Installation Procedure 6
1.1 Installation steps using executable file 6
1.1.1 Installation steps in Windows 6
1.1.2 Installation steps in MacOSX 7
1.1.3 Installation steps in Ubuntu 7
1.2 Installation steps using scripts/terminal commands 7
1.2.1 Installation steps in Windows OS using Anaconda/Python Terminal 8
1.2.2 Installation steps in Ubuntu OS 9
1.2.3 Installation steps in MacOSX 10
1.2.4 Installation steps for a General Operating system 11
1.3 BioSANS uninstall steps 11
2 Opening the BioSANS GUI 12
2.1 Steps to start BioSANS installed using executable 12
2.2 Steps to start BioSANS installed using script/terminal 12
2.2.1 STEPS to start BioSANS from a terminal/Python-associated terminal 12
2.2.2 General steps for any OS 13
3 Running Basic Simulations 14
3.1 Opening a topology file 14
3.2 Propagation of deterministic trajectory 15
3.3 Opening current working directory 17
3.4 Opening an output/settings file 18
4 Creating a Topology File 20
4.1 Basic topology file 20
4.2 Topology file with multiple independent reactions 21
4.3 Topology file with modified propensity 22
4.4 Topology file with concentration substitution 24
4.5 Topology file with conditional statement 26
4.5.1 Time-dependent events 27
4.5.2 Species-dependent events 27
4.5.3 Events with delay 28
4.6 Topology file with time-dependent propensity 28

4.7 Topology file with non-constant volume, stoichiometry, and rate constant 29
4.8 Encoding differential equation models into a topology file 30
4.8.1 The ODE file – an alternative input file 31
4.8.2 SBML files – most common file format in systems biology 32
4.8.3 List of math functions supported in SBML to topology file conversion 32
4.9 Topology file for parameter estimation 33
5 BioSANS Basic Analysis 34
6 BioSANS Modeling Tasks Use Cases and Workflow 38
6.1 GUI workflow 38
6.1.1 Steps to start BioSANS GUI 38
6.1.2 Loading topology or any file 38
6.1.3 Creating a new file 39
6.1.4 Saving file 39
6.1.5 Closing files 39
6.1.6 Modifying files (i.e., topology files) 39
6.1.7 Symbolic analytical expression for chemical species 39
6.1.8 Deterministic integration 40
6.1.9 Stochastic integration 41
6.1.10 Linear noise approximation (LNA) 41
6.1.11 Network localization 42
6.1.12 Parameter estimation 42
6.1.13 Post-processing/analysis 44
6.2 BioSANS as a Python import 45
6.2.1 List of method keywords 47
6.3 BioSANS console interface - structured simulation language (SSL) 48
6.3.1 List of currently supported commands: 49
7 List of Codes in BioSANS 51
7.1 Hierarchical tree structure of BioSANS 51
7.2 Description of codes in BioSANS 52
8 Deterministic Modeling Examples 54
8.1 Feed-forward loop with square wave input signal (Propagation) 54
8.2 Repressilator system using Hill function (Propagation, Phase portrait) 56
8.3 Lorenz system (Propagation, Phase portrait, 3D Phase portrait) 60

8.4 Simple gene expression model (symbolic LNA and species analytical expression) 63
8.5 Parameter estimation (A => B => C) 66
8.5.1 All rate constants unknown and all species trajectories are given 67
8.5.2 All rate constants unknown and some species trajectories are missing 67
8.5.3 Parameter estimation with propensity modification 68
8.6 Parameter estimation with Hill function 70
8.7 Parameter estimation at steady state (regular functions) 70
8.7.1 Finding roots ($ax^3 + bx^2 + cx + d = 0$) 71
8.7.2 Finding roots for systems of equation 71
9 Stochastic Modeling Examples 73
9.1 A + B <=> C => D + A 73
9.2 VGCN SYSTEM 75
9.3 A <=> B reversible reaction 79
10 Algorithm Implementation and Description 82
10.1 Symbolic computations 82
10.1.1 Species analytical expression as a function of time 82
10.1.2 LNA covariance matrix and steady-state concentration 83
10.1.3 Network localization 83
10.2 Numerical computations 84
10.2.1 Species concentration as a function of time 84
10.2.2 LNA covariance matrix 85
10.2.3 Estimation of rate constant given some experimental data 85
10.2.4 Propagation of stochastic trajectories 87
10.2.5 Miscellaneous functions 89
11 BioSANS testing results 90
11.1 Symbolic test cases 90
11.2 Performance on semantic test 93
11.3 Performance on stochastic tests 94
11.4 Performance in symbolic tests 95
11.5 Performance in parameter estimation 96
12 Additional Examples 97
12.1 BioSANS console interface (examples) 97
12.1.1 A => B => C (stochastic simulation) 97

12.1.2 Parameter estimation (A => B => C) 101
12.1.3 Symbolic computation 102
12.1.4 Propagation from topology file 102
12.1.5 Automated simulations in one file 103
12.2 Using BioSANS as a Python import 103
REFERENCES 105

1 BioSANS Installation Procedure

BioSANS is currently in Github and in test.pypi and pypi repositories. One can just download all source files and use Python to run the main package, which is "BioSANS2020". The installers and source code can be found in the following links;

Webpage:

https://efajiculay.github.io/SysBioSoft/

Source codes:

https://test.pypi.org/project/BioSANS2020/
https://pypi.org/project/BioSANS2020/
https://github.com/efajiculay/SysBioSoft/tree/BioSANS_updated/BioSANS/src

BioSANS executable installers:

https://github.com/efajiculay/BioSANS_installers

If a user is familiar with **anaconda terminal** and **python terminal**, the preferred or suggested way of installation is by using the **pip command** discussed in section 1.2 as follows;

```
pip install BioSANS2020 --upgrade
```

1.1 Installation steps using executable file

1.1.1 Installation steps in Windows

The following installation steps were tested in Windows 10. For earlier Windows versions, these steps still likely work. If the installation fails, please proceed to the steps in section 1.2.

1. Download any of the following executable files from our GitHub page https://efajiculay.github.io/SysBioSoft/

   BioSANS_installer_py3.7.4_x86_32.exe– for windows >XP
   BioSANS_installer_py3.7.4_x86_64.exe– for windows >XP
   BioSANS_installer_py3.9.5_amd32.exe – for windows > 7
   BioSANS_installer_py3.9.5_amd64.exe – for windows > 7

2. Right click the "*.exe" file and choose to run as administrator.
   Note: Installation by double click also works, but the user needs to allow the installation to proceed in the pop-up that will appear in the task bar.

3. The installation process may take a while for earlier Windows versions. The following link shows an example of what would appear during the installation process: https://youtu.be/g3ZmjWEGm40
4. The BioSANS shortcut can be seen on the desktop and in the start menu if the installation is successful.
5. Double click the shortcut to launch BioSANS graphical user interface (GUI).

1.1.2 Installation steps in MacOSX

The following steps were tested on MacOS Sierra. For other versions, these steps still likely work. If the installation fails, please proceed to the steps in section 1.2.

1. Download the following executable file from https://github.com/efajiculay/BioSANS_installers/tree/main/MacOSX
   MacOSX_Installer – no file extension
2. Double click this executable installer.
   For some Mac systems, add execution privilege to the installer first, which can be done with the following command using the Mac terminal.

```
cd Downloads   # or cd to the directory of downloaded file
chmod 777 MacOSX_Installer
```

3. Key in the password, press enter or type Y for the steps where it is required as the installation proceeds.
4. A BioSANS executable launcher is placed on the desktop and in Applications after the installation.
5. Double click the shortcut to launch BioSANS GUI.

1.1.3 Installation steps in Ubuntu

The following steps were tested in Ubuntu20.04. For other versions, these steps still likely work. If the installation fails, please proceed to the steps in section 1.2.

1. Download the following Ubuntu Installer executable file from https://github.com/efajiculay/BioSANS_installers/tree/main/Ubuntu
   Ubuntu_Installer – no file extension
2. Launch the Ubuntu Terminal using **Ctrl + t** and run the following commands in the terminal window:

```
cd Downloads   # or cd to the directory of downloaded file
./Ubuntu_Installer
```

3. Key in the Ubuntu password, press enter or type Y for the steps where required as the installation proceeds.
4. Files BioSANS.sh and BioSANS.desktop will appear on the desktop after the installation.
5. Right click the BioSANS.desktop file and click to allow launching.
6. Double click this shortcut to launch BioSANS.
7. Alternatively, BioSANS can be launched by running BioSANS.sh in the terminal.

1.2 Installation steps using scripts/terminal commands

BioSANS requires Python version ≥ 3.7 to properly work. Follow the Python installation procedure on designated Python websites. Python 3.7 cannot support Windows XP and earlier. This holds true for BioSANS as well.

1.2.1 Installation steps in Windows OS using Anaconda/Python Terminal

The following steps were tested in Windows 10 but likely work as long as the user first installs Anaconda or Python with Python ≥ 3.7. A lower version of Python might also work but with some performance issues.

1. Type Anaconda and/or cmd in the windows search box in the task bar

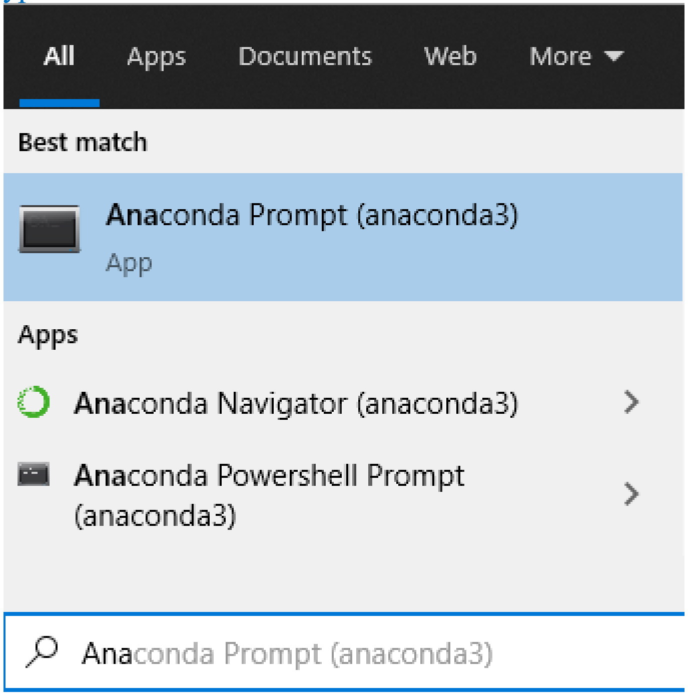

2. Click the Anaconda or the cmd terminal
3. In anaconda, a new environment can be created before installation. To create and activate a new conda environment called BioSANS, the following commands are needed:

```
conda create -n BioSANS python==3.9
conda activate BioSANS
```

4. For cmd and anacoda, just run the following to install BioSANS.

```
python -m pip install BioSANS2020 --upgrade
```

5. During installation, the process may ask for authorization and or may ask to continue the process. Type Y whenever necessary.
6. At the end of installation, you can run BioSANS in the following ways;

```
# To run BioSANS GUI, type any of the following commands
BioSANS
python -m BioSANS2020
python -m BioSANS2020.BioSANS

# To run BioSANS SSL console, type any of the following
commands
BioSSL
python -m BioSANS2020.BioSSL
```

1.2.2 Installation steps in Ubuntu OS

The following steps are tested for a freshly installed Ubuntu on a virtual machine. The Ubuntu image we used in the virtual machine is **ubuntu-20.04.1-desktop-amd64.iso.**

1. Open Ubuntu terminal (**Ctrl + t**).
2. Install using pip as follows;

```
python -m pip install BioSANS2020 --upgrade

or

python3 -m pip install BioSANS2020 --upgrade
```

3. During installation, the process may ask to continue the process, etc. Provide the details asked by the terminal to proceed in the installation.
4. At the end of installation, you can run BioSANS in the following ways;

```
# To run BioSANS GUI, type any of the following commands
BioSANS
python -m BioSANS2020
python -m BioSANS2020.BioSANS   # may need to use python3

# To run BioSANS SSL console, type any of the following
commands
BioSSL
python -m BioSANS2020.BioSSL   # may need to use python3
```

If the above steps did not work as expected, try the following steps in the Ubuntu terminal:

```
#The following script are optional
sudo apt update
sudo apt install software-properties-common
#sudo add-apt-repository ppa:deadsnakes/ppa   #optional

#If python3.7 or higher is not installed (normally already
have 3.8 by default)
sudo apt install python3
sudo apt-get install python3-tk
sudo apt install python3-distutils

#Installing pip here
sudo apt install curl
curl https://bootstrap.pypa.io/get-pip.py -o get-pip.py
python3 get-pip.py

#Installing BioSANS (The following command is one line)
python3 -m pip install BioSANS2020 --upgrade
```

1.2.3 Installation steps in MacOSX

The following steps requires python and or anaconda to be installed first by the user. During anaconda or python installation, the user should have ticked the add anaconda/python to the systems path. This will make python and anaconda accessible in MacOSX terminal.

1. Click the Launchpad.
2. Search for the terminal and open it.
3. Install using pip as follows;

```
python -m pip install BioSANS2020 --upgrade

or

python3 -m pip install BioSANS2020 --upgrade
```

4. During installation, the process may ask some details to continue the process, etc. Provide the details asked by the terminal.
5. At the end of installation, you can run BioSANS in the following ways;

```
# To run BioSANS GUI, type any of the following commands
BioSANS
python -m BioSANS2020          # may need to use python3
python -m BioSANS2020.BioSANS  # may need to use python3

# To run BioSANS SSL console, type any of the following
commands
BioSSL
python -m BioSANS2020.BioSSL   # may need to use python3
```

If the above steps did not work as expected, try the following steps in the terminal.

```
xcode-select —install

/bin/bash -c "$(curl -fsSL
https://raw.githubusercontent.com/Homebrew/install/master/i
nstall.sh)"

brew install python
python3 -m pip install --upgrade pip
brew install --cask anaconda

python3 -m BioSANS2020 --upgrade
```

1.2.4 Installation steps for a General Operating system

1. Install Python ≥ 3.7.
2. Open Python terminal.
3. Install pip.
4. Issue the following command in the Python terminal.

```
pip install BioSANS2020 --upgrade
```

   After this, BioSANS is already installed.

5. At the end of installation, you can run BioSANS in the following ways;

```
# To run BioSANS GUI, type any of the following commands
BioSANS
python -m BioSANS2020           # may need to use python3
python -m BioSANS2020.BioSANS   # may need to use python3

# To run BioSANS SSL console, type any of the following
commands
BioSSL
python -m BioSANS2020.BioSSL    # may need to use python3
```

1.3 BioSANS uninstall steps

BioSANS can be uninstalled by uninstalling via pip, uninstalling the corresponding Python installed during the BioSANS installation, and by deleting the BioSANS folder.

For Windows systems, if BioSANS was installed using the executable, one can uninstall the corresponding Python installed together with BioSANS in the control panel. One can also delete the specific BioSANS version from "C:\BioSANS2021". With BioSANS installed using the terminal, by Anaconda or a Python terminal, just issue the following commands:

conda activate BioSANS # for those who used anaconda
pip uninstall BioSANS2020

BioSANS can be uninstalled in any other OS using the above command in the default terminal.

2 Opening the BioSANS GUI

For any platform, a user needs to launch an anaconda terminal or any Python terminal before launching BioSANS. The libraries in **Table 1** are imported in the BioSANS codes and will normally be installed if the installation procedure in section 1.0 was followed. If this is the first time a user runs BioSANS, an error message would appear if there are missing libraries. Proceed to install the missing libraries using the commands provided in **Table 1**.

Table 1. List of libraries imported in BioSANS.

Library	Installation command	Library	Installation command
sys		libsbml	pip install python-libsbml
os		scipy	pip install scipy
datetime	pip install DateTime	warnings	
tkinter		random	
pathlib	pip install pathlib	math	
PIL	pip install Pillow<br>sudo apt-get install python-tk	ode_int	
numpy	pip install numpy	multiprocessing	
time		draw_figure	
threading	pip install thread6	re	
queue		inspect	
process		random	
pandas	pip install pandas	sde_int	pip install sdeint
matplotlib	pip install matplotlib==3.3.3	mpl_toolkits	
sympy	pip install sympy	func_timeout	pip install func_timeout

2.1 Steps to start BioSANS installed using executable

Double click the BioSANS shortcut created on the desktop. This shortcut is also available in the start menu in windows and in applications in Mac.

2.2 Steps to start BioSANS installed using script/terminal

The following steps assume that Python and pip are already installed.

2.2.1 STEPS to start BioSANS from a terminal/Python-associated terminal

1. Open the Anaconda/Python terminal.

2. For Anaconda-installed BioSANS, use the following command:

```
conda activate BioSANS

#Any of the following commands can lauched BioSANS
BioSANS
python -m BioSANS2020           #use python3 for Ubuntu/Mac
python -m BioSANS2020.BioSANS   #use python3 for Ubuntu/Mac

#Any of the following commands can lauched BioSSL
BioSSL
python -m BioSANS2020.BioSSL    #use python3 for Ubuntu/Mac
```

3. For terminal-installed BioSANS, run the commands in number 2 except for the first line, which is for Anaconda activation.

2.2.2 General steps for any OS

1. Open Python terminal or default OS terminal
2. Run the following command except for Anaconda activation if the terminal used during the installation is not Anaconda.

```
conda activate BioSANS   # not needed if conda environment
                         # is not created or if not using
                         # anaconda

#Any of the following commands can lauched BioSANS
BioSANS
python -m BioSANS2020           #use python3 for Ubuntu/Mac
python -m BioSANS2020.BioSANS   #use python3 for Ubuntu/Mac

#Any of the following commands can lauched BioSSL
BioSSL
python -m BioSANS2020.BioSSL    #use python3 for Ubuntu/Mac
```

3 Running Basic Simulations

To run basic simulations, a user needs to load a topology file. The basic component of a topology will be discussed in section 4.0. For this section, we will use the topology files in the "**TutorialTopoFiles,**" which can be downloaded at the following link:

https://github.com/efajiculay/BioSANS_installers/tree/main/TutorialTopoFiles

3.1 Opening a topology file

BioSANS has 3 main Tabs: the **File**, **Analysis**, and **Post proc** tabs. The **File** tab contains the **Open** menu, which has a submenu containing the **Topology/File** option. The following steps are needed to open a topology file.

***Warning: For Ubuntu OS, use arrow keys to navigate the sub-menu after clicking*.**

1. Click File => Open => Topology file

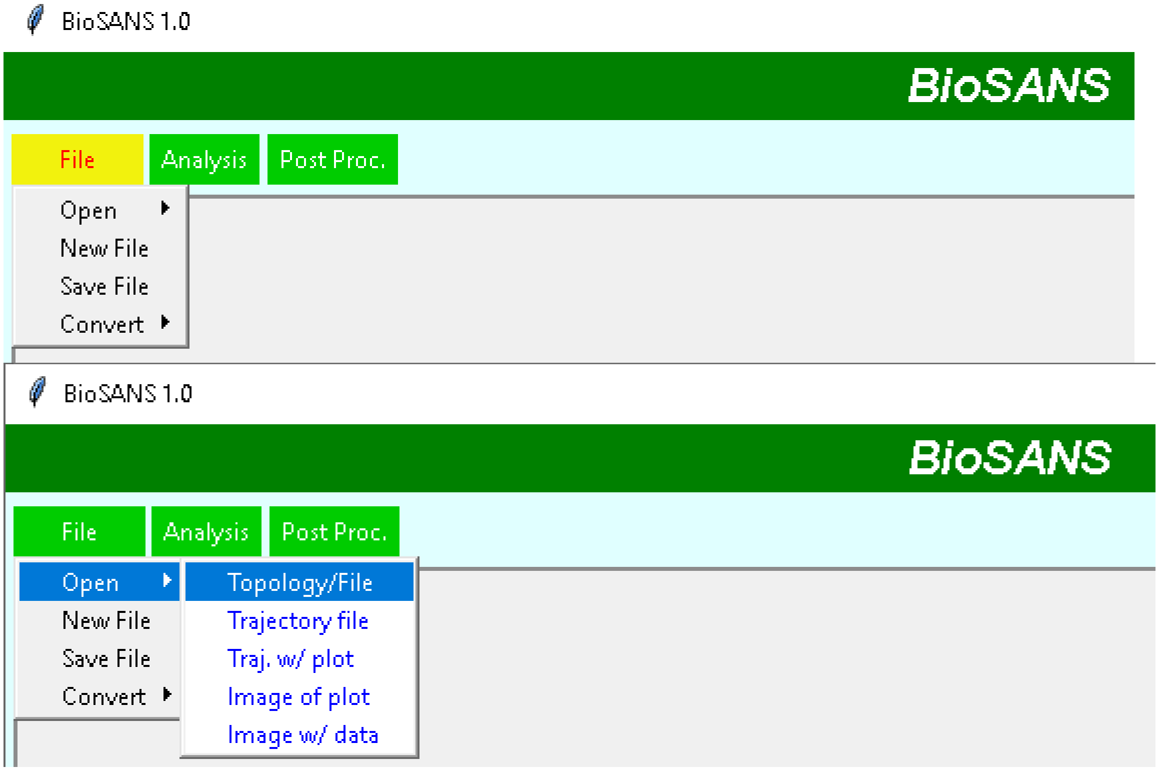

2. Browse the topology file. In this tutorial Browse for Q1a.txt

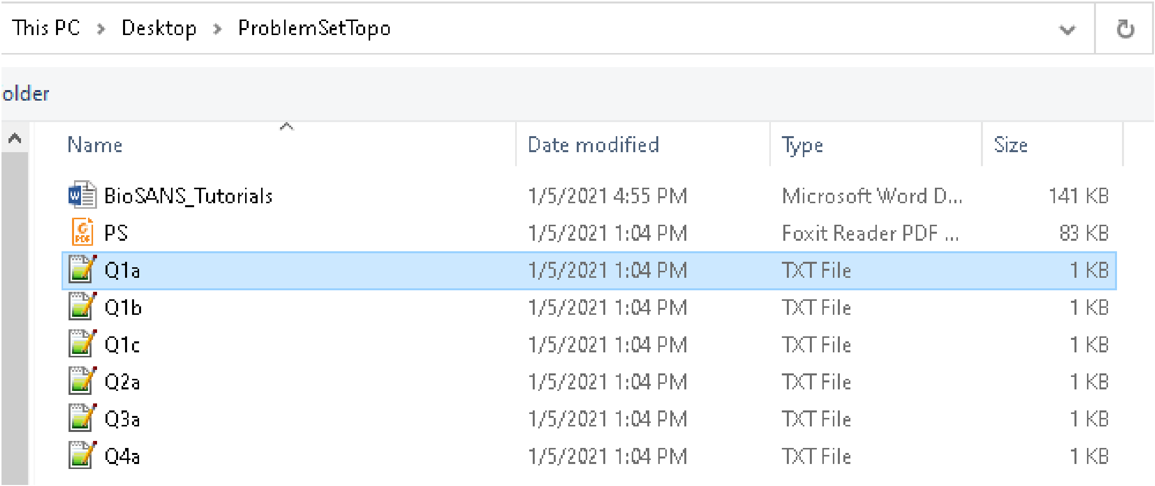

3. The content of the file will show up in the main window.

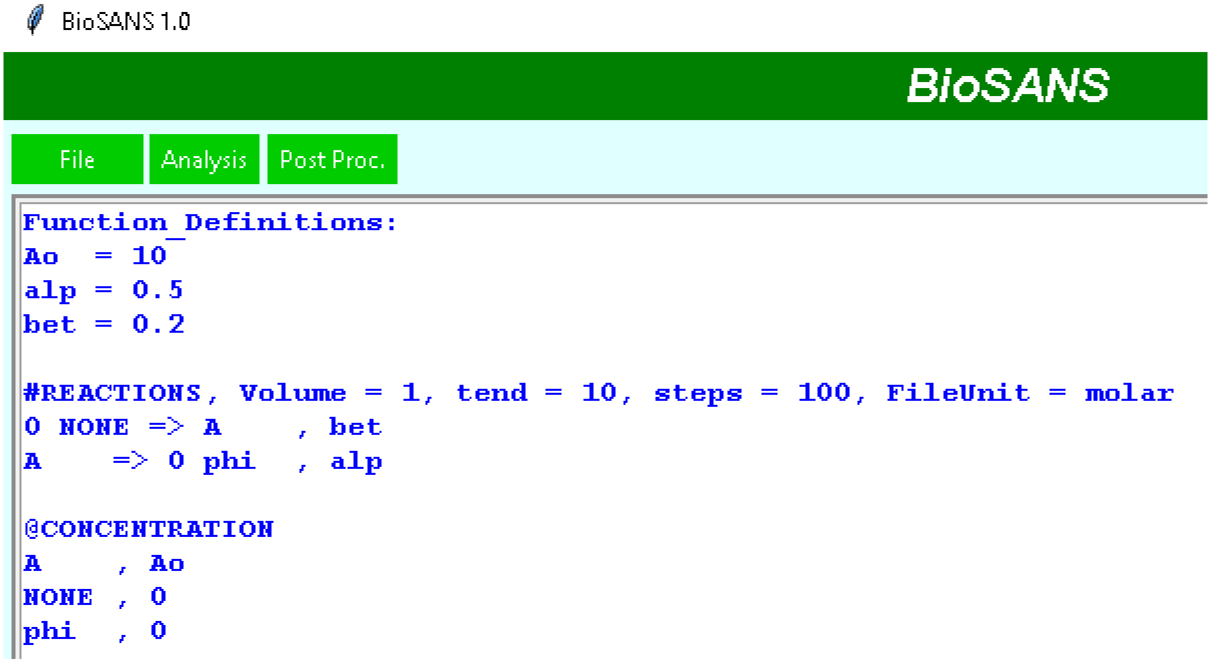

A new file can also be created by selecting the following sequence: File => New File => Blank file. This will show a new text area where a user can type or paste some content (which can be topology). The content can be saved by File => Save File.

3.2 Propagation of deterministic trajectory

This section focuses on the basic use of BioSANS for propagation of deterministic trajectory. With a few clicks, a user can easily poduce trajectories from a given topology file. The following steps represent one of the ways to propagate a deterministic trajectory:

1. Click Analysis => ODE int => molar (macro)

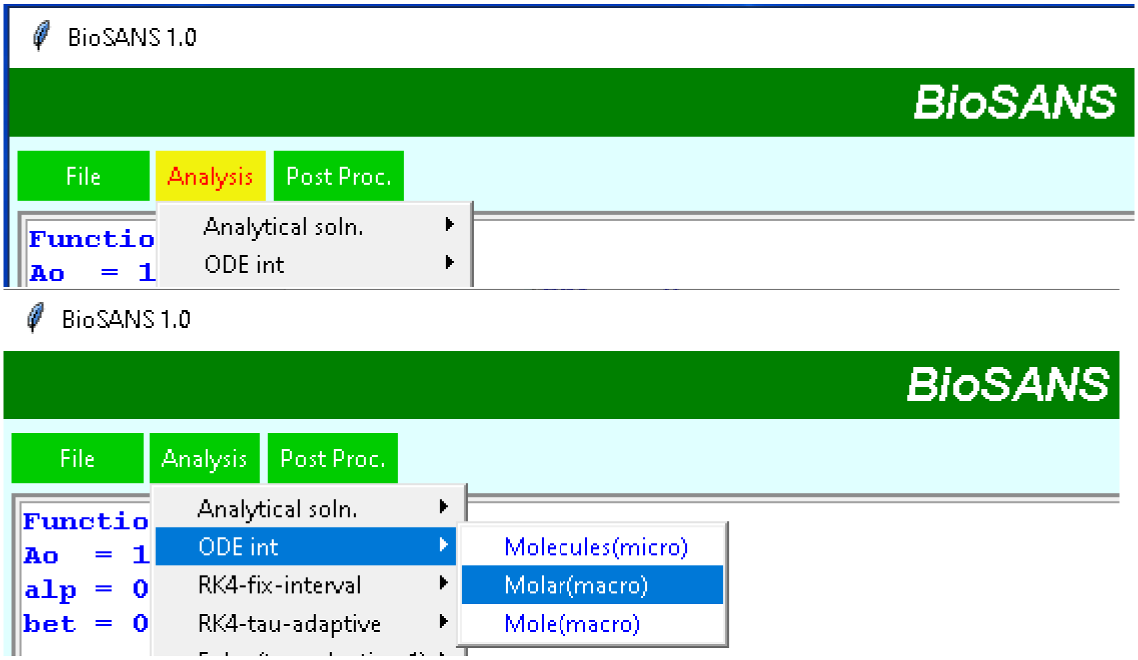

2. Modify parameter setting as needed

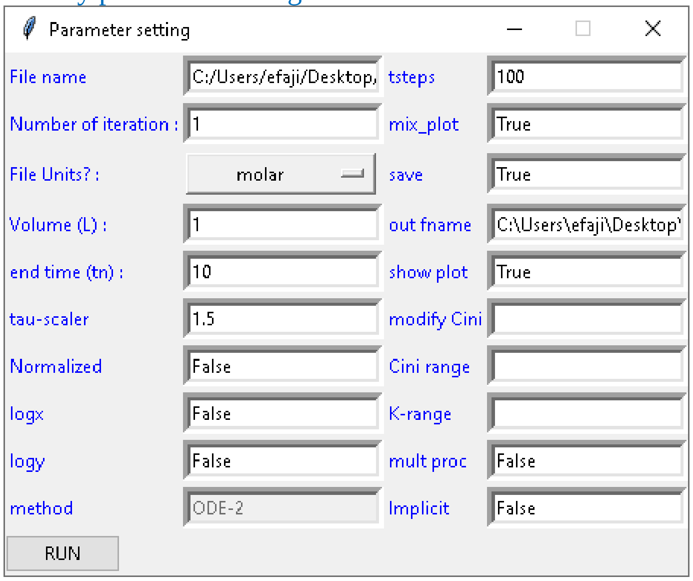

3. Click run

The parameter setting grabs some information from the topology file if it is provided in the same line as the #REACTIONS tag. Some predefined settings, if provided, are automatically selected.

The following image is the output from Q1a.txt in the **ProblemSetTopo** folder provided with this tutorial:

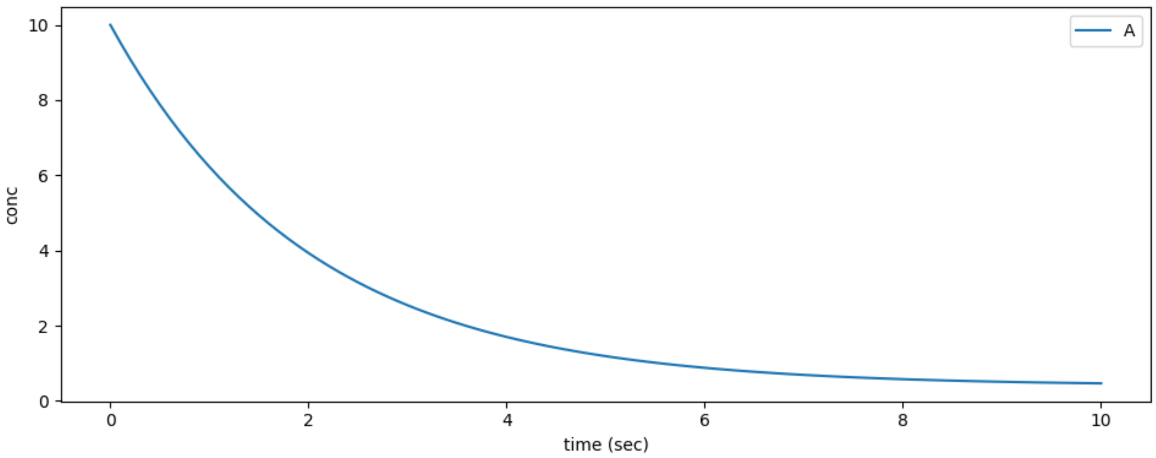

Figure 1. Output plot of Q1a.txt topology file after the simulation.

3.3 Opening current working directory

After performing propagation, the trajectory file is saved in the same directory where the topology files reside. To open the current working directory from BioSANS, follow this step:

File/Model => Open => Current Folder

Check the folder where the topology file "Q1a.txt" is. There are new files created after the run.

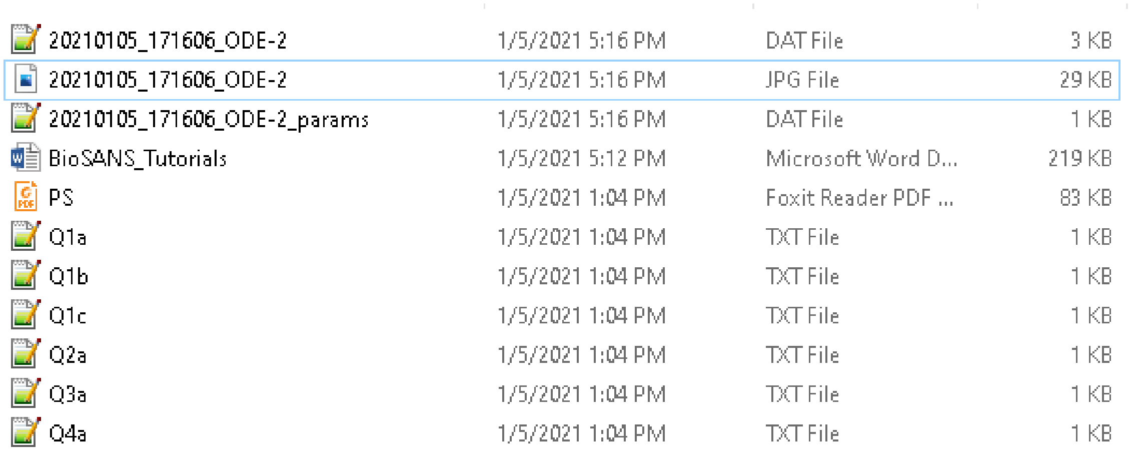

Figure 2. Trajectory, parameter details and plot of Q1a.txt topology file after the simulation.

The first 3 files in **Figure 2** are the output from Q1a simulation. The files are labelled with the current date and time and the method used to run the integration. The file with "**_params**" contains the parameter settings chosen. The **.jpg** file contains the plot and the **.dat** file contains the trajectory. These 3 files always have a very similar filename every run, which allows the user to identify them. The first line in the "_params.txt" file identifies which topology was associated with this run. In the "_params.txt" file in **Figure 3**, the first line contains the path to **Q1a.txt**. Hence, all files with "20210105_171606" in their name are associated with **Q1a.txt**.

A user can open the **.dat** file using notepad, any text editor, or BioSANS itself. There are two **.dat** files: one contains the trajectory and the other the settings used when producing the trajectory.

3.4 Opening an output/settings file

After the simulation, a user may want to see the trajectories and parameter settings used. This can be done by following these steps:

Click File => Open => Topology/File => The file you wish to see

For this tutorial, open “20210105_171606_ODE-2_params.dat”. The name will be different on each machine, but it is the file with the **“_params.dat”** in its file name. This file contains the parameters chosen in step 2 of propagating trajectories.

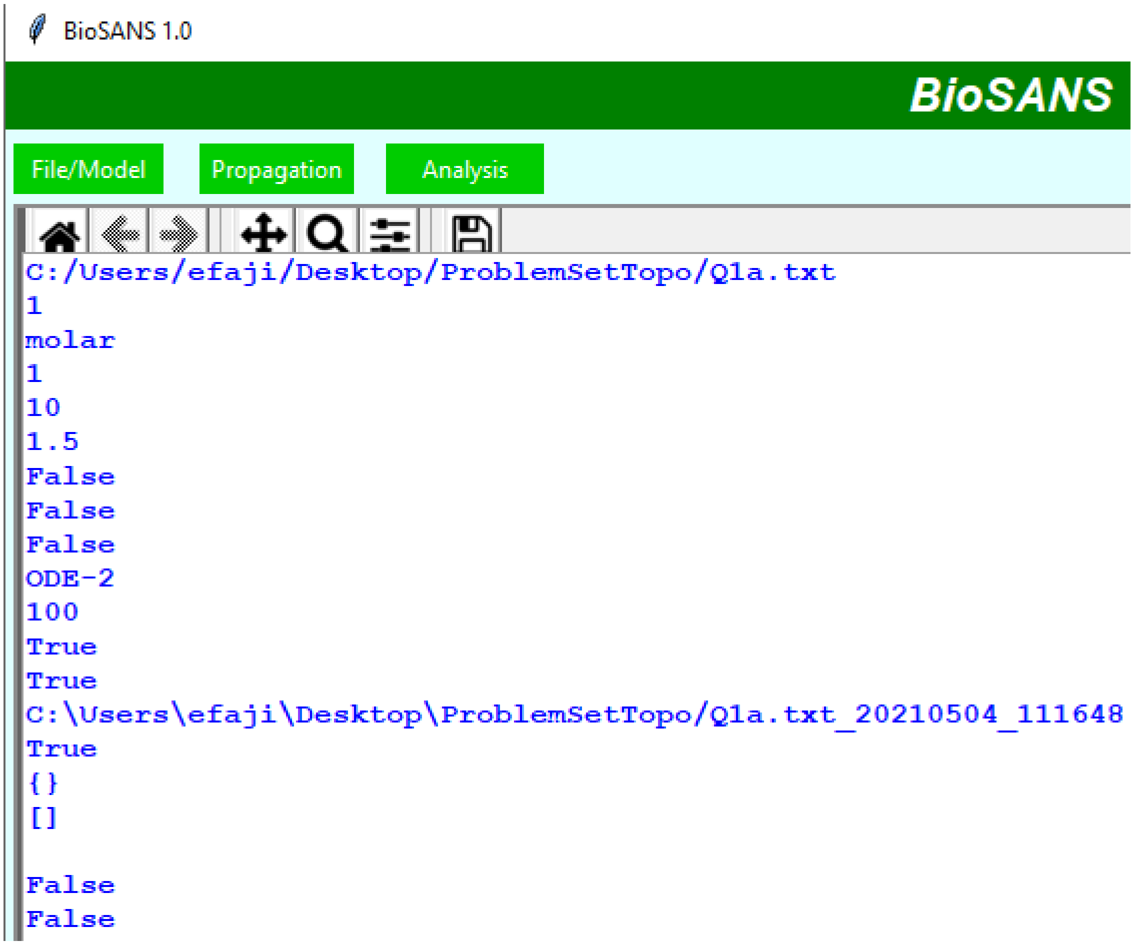

Figure 3. The parameter settings chosen for the Q1a.txt topology file.

Now, open the actual trajectory file. It is the **.dat** file ***without*** **“_params”** in its filename.

Click File => Open => Topology/File => “20210105_171606_ODE-2.dat”

The trajectories will show up in a text area for viewing. No further analysis can be done unless this trajectory is loaded in memory.

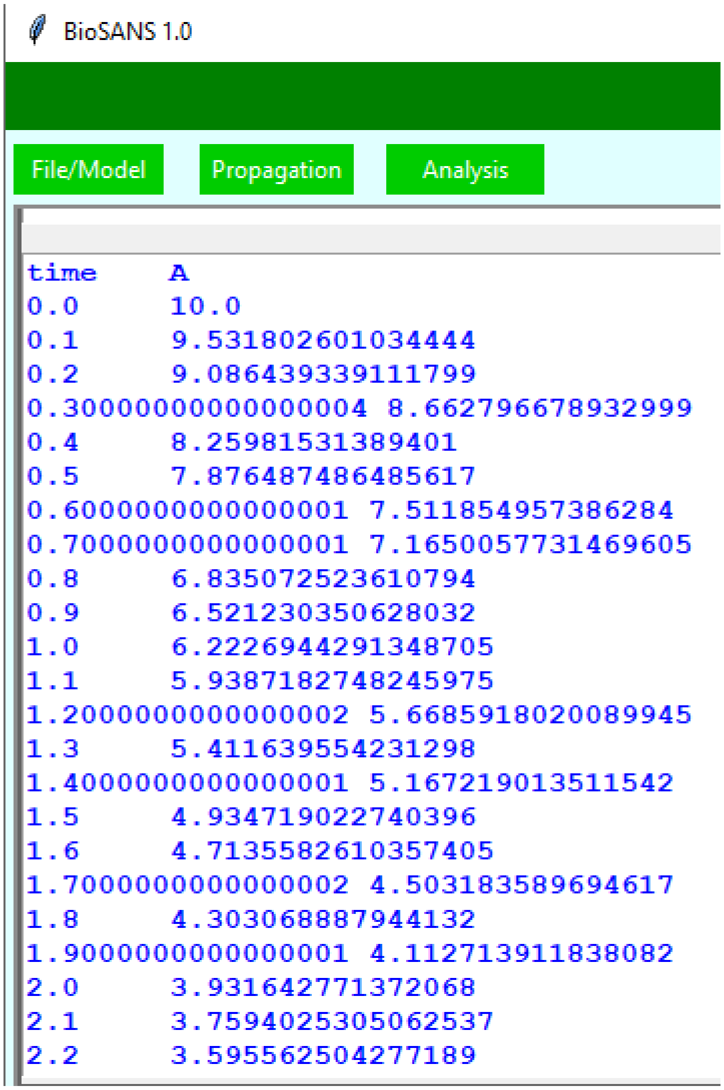

Figure 4. Portion of the trajectory after running the Q1a.txt topology file.

To load the trajectory in memory and for further analysis, follow the following sequence of steps:

Click File => Open => Trajectory/File => “Your trajectory file”

and choose among the analysis options in the **Analysis** tab.

4 Creating a Topology File

4.1 Basic topology file

The topology file is the main input file in BioSANS. It contains tags before each entry that describe what the following entries are. There are 3 main tags in BioSANS:

> **Function_Definitions:**
> **#REACTIONS**
> **@CONCENTRATION**

The **Function_Definitions:** tag tells BioSANS that the following expression is an assignment or a one-line lambda function declaration. This tag is optional but helps define variables and functions in one place and use them in the topology file. The **#REACTIONS** tag is required and means that the following entries are a list of reactions in the system. The **@CONCENTRATION** tag is also required and is followed by the list of initial conditions. Below is an example of a typical BioSANS topology file. We can see that the **Function_Definitions:** is followed by a declaration of constants in the system. If we don't want to define them, we can just use actual values when those variables are present.

```
Function_Definitions:
Ao   = 10
alp = 0.5
bet = 0.2

#REACTIONS, Volume = 1, tend = 10, steps = 100, FileUnit = molar
0 NONE => A      , bet
A       => 0 phi , alp

@CONCENTRATION
A     , Ao
NONE  , 0
phi   , 0
```

Figure 5. Example of topology file showing basic tags in BioSANS topology file.

The **#REACTIONS** tag can have additional information, including **Volume,** which refers to the volume of the system; **tend,** the end time of simulation; **steps,** the number of steps taken from time = 0 to time = tend; and **FileUnit,** the concentration unit used in the file. The rate constant unit if unit applies will follow the molecularity of the corresponding reaction but using the FileUnit declared for the amount raised to corresponding power based on molecularity. Following the **#REACTIONS** tag are the chemical reactions in the system. In
Figure 5, we can see the following reactions:

```
0 NONE => A      , bet
A       => 0 phi , alp
```

Those reactions pertain to the formation or production of A and degradation of A, respectively. In each line, the rate constant follows after the comma (,). If there are 2 rate constants (also 2 commas), then the reaction is reversible. In this reaction, we don't know what is the source of A and we don't know what is the degradation product. The 0 (zero) before NONE and 0 (zero) before phi only tells BioSANS that those species are not needed and will not be shown in the plot. For the production of A, it also means zero order and independent of the source. The above reaction will translate to the following ordinary differential equation:

$$\frac{dA}{dt} = bet * (None)^0 - alp * A$$

which can be simplified as

$$\frac{dA}{dt} = bet - alp * A$$

For the **@CONCENTRATION** tag, ***all species appearing in the reaction including NONE and phi should be declared with initial concentration***. For NONE and phi, we can declare them as zero because they are not needed but only serve as a place holder for reactant and product.

4.2 Topology file with multiple independent reactions

BioSANS allows for multiple independent reactions for deterministic computation in one topology file. For stochastic simulation, we can also do this except for Gillespie and tau-leaping2 because we need to isolate their treatment of propensities, which is currently not supported.

Figure 6 is an example of multiple instances of the same reaction with varying initial conditions, which can be summarized in the following ordinary differential equation (ODE):

$$\frac{dA}{dt} = bet - alp * A$$

There are 4 different initial conditions, which in **Figure 6** is represented as A0, A5, A8, and A10. If we run this file, we will see the behavior of the plot at varying initial conditions.

```
Function_Definitions:
alp = 0.5
bet = 0.2

#REACTIONS, Volume = 1, tend = 10, steps = 500, FileUnit = molar
0 NONE => A10     , bet
A10    => 0 phi   , alp

0 NONE  => A8     , bet
A8      => 0 phi  , alp

0 NONE => A5      , bet
A5     => 0 phi   , alp

0 NONE => A0      , bet
A0     => 0 phi   , alp

@CONCENTRATION
A10     , 10
A8      , 8
A5      , 5
A0      , 0
NONE    , 0
phi     , 0
```

Figure 6. Example **of** topology file showing multiple independent reactions with varying initial conditions.

4.3 Topology file with modified propensity

BioSANS also offers a way to modify the propensity of a reaction if such reaction does not follow mass action kinetics. If no modification is provided, BioSANS will assume mass action expression to the reactions. The following file shows some modified propensity after the reactions rate constant. The modification is the incorporation of a Hill function.

In **Figure 7**, lines 11 and 14 have "**:::::**", which serves as a delimiter that tells BioSANS to use the expression after "**:::::**" instead of using a mass action expression and the rate constant provided. For example, in the following reaction,

```
0 NONE => A     , 1   ::::: lambda R : betA*K/(K+R)
```

whatever we put after the comma will not be used but we still need to put something as a place holder of the rate constant. The corresponding ODE of the reaction in **Figure 7** is as follows:

$$\frac{dA}{dt} = betA * K/(K + R) - alpA * A$$

$$\frac{dR}{dt} = betR * A/(K + A) - alpR * R$$

```
Function_Definitions:
alpA = 0.5
betA = 0.2

alpR = 0.5
betR = 0.2

K = 0.2

#REACTIONS, Volume = 1, tend = 10, steps = 100, FileUnit = molar
0 NONE => A       , 1   ::::: lambda R : betA*K/(K+R)
A      => 0 phi   , alpA

0 NONE => R       , 1   ::::: lambda A : betR*A/(K+A)
R      => 0 phi   , alpR

@CONCENTRATION
A    , 0
R    , 0
NONE , 0
phi  , 0
```

Figure 7. Example of topology file showing multiple reaction with modified propensity.

How to construct the lambda expression. The lambda expression consists of 2 things delimited by **":"**. The left-hand side is the keyword lambda itself followed by variables (separated by comma) needed in the expression in the right-hand side (RHS). All variables in the RHS that are ***not declared*** in the **Function_Definitions:** should be listed (separated by comma) after the lambda keyword. In **Figure 7**, we saw the following expression:

lambda R **:** betA*****K/**(**K**+**R**)**

We can see that R follows the lambda keyword because R is the only variable in the RHS of "**:**" that is not declared in the **Function_Definitions:**. If there is more than one variable in RHS that is not declared, we can have more variables after lambda keyword such as the following:

lambda x,y,z : x*y+y*z+10

Another example of the use of a lambda expression can be found in **Figure 8**. We can see that X and Y variables are not declared in the **Function_Definitions:** and they appear after the lambda keyword.

```
Function_Definitions:
alpX = 0.5
betX = 0.2

alpY = 0.55
betY = 0.25

K = 0.2
n = 2

#REACTIONS, Volume = 1, tend = 100, steps = 100, FileUnit = molar
0 NONE => X       , 1    ::::: lambda Y : betX*(K**n)/(K**n+Y**n)
X      => 0 phi   , alpX

0 NONE => Y       , 1    ::::: lambda X : betY*(K**n)/(K**n+X**n)
Y      => 0 phi   , alpY

@CONCENTRATION
X    , 5
Y    , 2
NONE , 0
phi  , 0
```

Figure 8. Additional example of modified propensity function.

4.4 Topology file with concentration substitution

BioSANS also offer concentration substitution or species substitution in the entries after the **@CONCENTRATION** tag. This is another way of incorporating a special function without altering the propensity. **Figure 9** is an example of a topology file with concentration substitution. Lines 29, 30, and 31 have the lambda function separated by a comma from the declared initial concentration. The initial concentration will not be used and the lambda expression will be evaluated at every time point and will be taken as the value of -Sa, -Sb, and -Sc. Here, the negative sign in the species tells BioSANS not to plot -Sa, -Sb, and -Sc. It will still work even if we just use Sa, Sb, and Sc, but they will be plotted.

The reactions in **Figure 9** translate to the following ODE expression:

$$\frac{dmA}{dt} = 100 * [-Sa] - 1 * mA = 100 * \frac{1}{1 + C^2} - 1 * mA$$

$$\frac{dA}{dt} = 1 * mA - 1 * A$$

$$\frac{dmB}{dt} = 100 * [-Sb] - 1 * mB = 100 * \frac{1}{1 + A^2} - 1 * mB$$

$$\frac{dB}{dt} = 1 * mB - 1 * B$$

$$\frac{dmC}{dt} = 100 * [-Sc] - 1 * mC = 100 * \frac{1}{1 + B^2} - 1 * mC$$

$$\frac{dC}{dt} = 1 * mC - 1 * C$$

```
#REACTIONS, Volume = 1, tend = 100, steps = 100, FileUnit = molar

-Sa     => mA          , 100
mA      => 0 phi       , 1
mA      => A + mA      , 1
A       => 0 phi       , 1

-Sb     => mB          , 100
mB      => 0 phi       , 1
mB      => B + mB      , 1
B       => 0 phi       , 1

-Sc     => mC          , 100
mC      => 0 phi       , 1
mC      => C + mC      , 1
C       => 0 phi       , 1

@CONCENTRATION, UNITS = molecules, VOLUME = 1 Liter

mA     , 1
mB     , 0
mC     , 0

A      , 0
B      , 0
C      , 0

phi    , 0
-Sa    , 0    ,lambda C : 1/(1+C**2)
-Sb    , 0    ,lambda A : 1/(1+A**2)
-Sc    , 0    ,lambda B : 1/(1+B**2)
```

Figure 9. Example of topology file showing modified concentration after the initial concentration declared. In this file, the lambda expression will be evaluated every time instead of using the propagated ODE concentration and initial concentration.

We did not show the ODE expression for -Sa, -Sb, and -Sc because they will just be substituted anyway. The trajectory obtainable from **Figure 9** can also be reproduced by using pure mass action kinetics but one needs to choose the proper rate constant such that upon simplification, the form of the Hill function in **Figure 9** will come out.

We note that the model shown in **Figure 9** is essentially a simplified allosteric expression for the binding of C to Sa (and A to Sb, B to Sc). The sequential binding of these factors can be laid out, forming a model shown in **Figure 10**. By assuming the fast equilibrium of these bindings and association, the mass-action kinetics can be simplified to form the Hill expressions seen in the model depicted in **Figure 9**. Details and discussion can be found in [1,2].

```
#REACTIONS, Volume = 1, tend = 100, steps = 100, FileUnit = molar
Sa  + C <=> SaC       , 1000  , 100000
SaC + C <=> SaC2      , 100000, 1000
Sa       => mA + Sa   , 100
mA       => 0 phi     , 1
mA       => A + mA    , 1
A        => 0 phi     , 1

Sb  + A <=> SbA       , 1000  , 100000
SbA + A <=> SbA2      , 100000, 1000
Sb       => mB + Sb   , 100
mB       => 0 phi     , 1
mB       => B + mB    , 1
B        => 0 phi     , 1

Sc  + B <=> ScB       , 1000  , 100000
ScB + B <=> ScB2      , 100000, 1000
Sc       => mC + Sc   , 100
mC       => 0 phi     , 1
mC       => C + mC    , 1
C        => 0 phi     , 1

@CONCENTRATION
mA    , 1
mB    , 0
mC    , 0

A     , 0
B     , 0
C     , 0

phi   , 0
Sa    , 1
Sb    , 1
Sc    , 1

SaC   , 0
SbA   , 0
ScB   , 0

SaC2  , 0
SbA2  , 0
ScB2  , 0
```

Figure 10. Mass action equivalent of **Figure 9**.

In **Figure 10**, lines 3, 4, 10, 11, 17, and 18 are reversible reactions. This is because there are 2 rate constants separated by a comma after the reaction and the arrow is a double-headed arrow.

4.5 Topology file with conditional statement

BioSANS supports conditional statements on the topology file, which allows for handling events, events with delay, etc. However, the ode_int from Python does not support this, so

we need to use another integrator in BioSANS for this type of topology. This is quite complicated, but upon mastery of this strategy, the topology file will be like a programmable input file.

4.5.1 Time-dependent events

```
Function_Definitions:


#REACTIONS
0 NONE =>         A, 0
0 NONE =>   timer_A, 1

@CONCENTRATION
timer_A,    0, lambda timer_A  :  0 if timer_A >= 30 else timer_A
A,         10, lambda timer_A  :  0 if timer_A >= 15 else 10
NONE, 0
```

Figure 11. Example of conditional statements in a topology file creating a square wave signal.

In **Figure 11**, we show a time-dependent event. To invoke time dependency, we need to declare a timer to the species (i.e., A) in both the reaction tag, and concentration tag. The format of the timer is "**timer_species**". If we ***do not follow*** this format, it will be treated as a regular species and will still work (not properly for Gillespie and tau-leaping2), but it will be plotted. In the reaction tag, we can see that the timer is represented as a reaction with "**0 NONE**" in the reactant and "**timer_A, 1**" in the product. This is because the rate of change of the timer should be equal to the rate of change of time, which is 1. In the concentration tag, the "**timer_A**" is declared with 0 initial amount to make its value initially equal to actual time. The lambda expression after the comma tells us that the value of "**timer_A**" is 0 if "**timer_A ≥ 30**", else "**timer_A**". This means that whenever it reaches the value of 30 during ODE propagation, its value will be reset to zero and will increase again until reaches 30 and reset again and repeat this process. The interesting part is what happens to species A. Here, "**A**" is declared with initial value 10. The lambda expression after the comma tells us that if **"timer_A ≥ 15"**, the value of **"A"** will be substituted with 0, else substituted with 10. Since the "**timer_A**" value is periodic from 0 to 30 back to 0, the value of **"A"** will also be periodic with an initial value of 10 when "**timer_A**" is still < 15, and 0 when "**timer_A**" is ≥ 15. The overall effect of this is a square wave. The **"0 NONE => A, 0"** after the reaction tag is not used but is still needed because BioSANS requires species to be both in reaction and concentration tags. If we omit it, there will be an error. It only says that **"A"** is formed from nothing with 0 rate.

4.5.2 Species-dependent events

Species-dependent events are much simpler than time-dependent events because we just need to focus on the lambda expression. It is normally a conditional statement that fires after meeting some concentration rules.

```
Function_Definitions:

#REACTIONS
0 NONE =>  A, 10

@CONCENTRATION
A    ,    0, lambda A  :  0 if A >= 15 else A
NONE,    0
```

Figure 12. Species-dependent event.

Figure 12 shows an example of species-dependent event. The concentration of A will be substituted by 0 when during propagation A reaches 15 then increased again from zero and this process is repeated.

4.5.3 Events with delay

This can be mimicked by combining several timers. The logic can be very complicated, but almost anything is possible when we combine several conditional statements.

```
Function_Definitions:

#REACTIONS
0 NONE =>  A       , 10
0 NONE =>  timer_A,  1

@CONCENTRATION
timer_A, 0, lambda A, timer_A  :  timer_A if A >= 15 else 0
A      , 0, lambda A, timer_A  :  0 if timer_A >=  5 else A
NONE   , 0
```

Figure 13. Topology file example for events with delay.

Figure 13 shows how we can achieve events with delay by combining species-dependent and time-dependent events. The **"timer_A"** has a value of 0 and will only be propagated when A is ≥ 15. When **"A"** reaches 15, **"timer_A"** will start increasing from 0 and after it reaches the value of 5, the concentration of **"A"** will reset to 0. When A is < 15, the value of **"timer_A"** resets to 0. This process continues until the end time of simulation.

4.6 Topology file with time-dependent propensity

Similar to what was discussed earlier in time-dependent events, we also need a timer for time-dependent species in their propensity. **Figure 14** shows a **"timer_A"** that serves as a timer for species **"A"**. Each species can have its own timer and can also have a common timer. Other species can also use a timer for other species.

```
Function_Definitions:


#REACTIONS
0 NONE =>  A, 0           ::::: lambda timer_A : sin(timer_A)
0 NONE =>  timer_A, 1

@CONCENTRATION
timer_A, 0
A      , 0
NONE   , 0
```

Figure 14. Topology file with time-dependent propensity.

Time-dependent propensity is supported in both deterministic and stochastic settings in BioSANS.

4.7 Topology file with non-constant volume, stoichiometry, and rate constant

BioSANS supports multiple independent reactions in one topology file as mentioned in section 4.2. We need to take advantage of this to facilitate non-constant values for volume, stoichiometry, and rate constants. This is similar to the timers we previously showed.

```
Function_Definitions:
kf1 = 0.5
kf2 = 0.5

#REACTIONS, tend = 10, tlen = 100
0 NONE =>  A         , 1       ::::: lambda Volume :kf1/Volume
      A =>  0 NONE    , 1       ::::: lambda A, Volume :kf2*A/Volume
0 NONE =>  Volume    , 0.5

@CONCENTRATION
Volume , 1
A      , 0
NONE   , 0
```

Figure 15. Non-constant volume in topology file.

The **"Volume"** of the compartment in **Figure 15** changes with a rate of 0.5, which affects the rate of formation and degradation of species **"A"**. However, this is not suitable for stochastic simulation unless the volume is assumed to be stochastic as well. To make it suitable for stochastic simulation, we put a "timer_" tag on it, which makes BioSANS think it is not a species and will be treated differently. So instead of using just **"Volume"**, we can use **"timer_Volume"**. This does not mean it is related to time but just to tag the volume as not a species. When we do so, we can use this for stochastic simulation, but it will not be plotted because it is not considered a species.

```
Function_Definitions:

#Reactions Volume = 1.0, tend = 2, steps = 100
1.0 S1   => 1.0 S2 ,1    :::::   lambda S1,k1 :1.0*(1.0)*k1*S1
0 NONE   => k1      , 0.5

@Concentrations
S1    , 0.15
S2    , 0.0
k1    , 1.0
NONE  , 1.0
```

Figure 16. Non-constant rate constant in topology file.

The analogy of the change in rate constant shown in **Figure 16** and the change in stoichiometry shown in **Figure 17** is similar to that in non-constant volume. To make this applicable to stochastic analysis, we can also put a **"timer_"** tag on the symbols that are not meant to be stochastic.

```
Function_Definitions:
kf = 0.2

#Reactions Volume = 1.0, tend = 10, steps = 100
1.0 A   => 1.0 B    , 1    :::::   lambda A,S : S*kf*A
0 NONE   => S       , 1

@Concentrations
A     , 0.15
B     , 0.0
S     , 1.0
NONE  , 1.0
```

Figure 17. Non-constant stoichiometry in topology file.

4.8 Encoding differential equation models into a topology file

Sometimes we do not have a chemical reaction in mind but we have a differential equation that describes the system. In BioSANS, differential equations can be encoded in the same way reactions are written, but we need to override the propensity using a lambda function. Consider the following differential equation from the Lorenz system:

$$\frac{dx}{dt} = 10\,(y - x)$$

$$\frac{dy}{dt} = -xz + 28x - y$$

$$\frac{dz}{dt} = x * y - \frac{8}{3}z$$

To encode them in BioSANS using the same algebraic expression as the ODE above shows, we need to write the variables as a formation reaction coming from “**0 NONE**” and forming the desired variable with a dummy rate constant that will not be used.

```
#REACTIONS
0 NONE => x, 1 ::::: lambda x, y    : 10*(y-x)
0 NONE => y, 1 ::::: lambda x, y, z : -z*x+28*x-y
0 NONE => z, 1 ::::: lambda x, y, z : x*y-(8/3)*z

@CONCENTRATION
x   , 9
y   , 9
z   , 28
NONE, 0
```

Figure 18. Encoding the Lorenz ODE into a topology file.

In **Figure 18**, the lambda expression after the “:::::” contains the actual expression in the ODE above. The formation reaction before the “,” indicates only the differential equation of the formed variable. The rate constant here is not used but only serves as a place holder.

Topology files with **multiple terms** in the algebraic propensity may **not** be **suitable** for **stochastic** propagation because it implies multiple reactions represented as one algebraic expression. This problem should be broken down into smaller ODEs such that each line in the topology is just one reaction or one unique propensity. In such case, BioSANS provides an ODE file input that can be converted by BioSANS into a topology file suitable for stochastic propagation.

4.8.1 The ODE file – an alternative input file

The BioSANS ODE file contains only a few tags as shown in **Figure 19**. This is to provide a way to define models using mathematical equations that can be translated by BioSANS into elementary reactions. The resulting reactions are in topology file format that can be propagated even with stochastic methods because propensity uniqueness is handled when inferring the reaction from the ODE expressions.

```
ODE_DECLARATIONS:
A = -ka*A*B/(1+C**2) + kf1/(1+B**2)
B = -ka*A*B/(1+C**2)
C = -kc*C + kf2
D = ka*A*B/(1+C**2) - kf2

INI_CONCENTRATIONS:
A = 100
B = 200
C = 150
D = 0

RATE_CONSTANTS:
ka = 0.02
kf1 = 0.2
kc = 0.03
kf2 = 0.01
```

Figure 19. Example of an ODE file in BioSANS.

The **ODE_DECLARATIONS:** tag enumerates the list of ODEs that describe the system. The species that change over time are equated with an equal sign to the ODE expression of behavior. The **INI_CONCENTRATIONS:** and **RATE_CONSTANTS:** tags enumerate the conditions.

The ODE file needs to be converted to a topology file before propagation can be done. BioSANS offers this conversion feature. The limitation of the ODE file is that it does not support conditional statements. When we convert it to a topology file, we can add the needed conditional statement if there is one.

4.8.2 SBML files – most common file format in systems biology

Systems biology markup language (SBML)[3] is a common model file in systems biology; it serves as a common format for software to interchange and use previous models. There have been repositories of models using the SBML format. BioSANS supports SBML file conversion to topology files facilitating propagation from models provided in this format. BioSANS also supports topology-file to SBML-file conversion but this is currently not tested.

4.8.3 List of math functions supported in SBML to topology file conversion

Table 2. List of SBML functions supported in conversion from SBML to topology files.

abs(x)	atanh(x)	sqr(x)
acos(x)	arctanh(x)	sqrt(x)
arccos(x)	ceil(x)	sin(x)
acosh(x)	ceiling(x)	sinh(x)
arccosh(x)	cos(x)	tan(x)
acot(x)	cosh(x)	tanh(x)
arccot(x)	cot(x)	And(*x)
acoth(x)	coth(x)	Not(x)
arccoth(x)	csc(x)	Or(*x)
acsc(x)	csch(x)	xor(*x)

arccsc(x)<br>acsch(x)<br>arccsch(x)<br>arcsec(x)<br>asech(x)<br>arcsech(x)<br>asin(x)<br>asinh(x)<br>arcsinh(x)<br>arcsin(x)<br>atan(x)<br>arctan(x)	factorial(x)<br>exp(x)<br>floor(x)<br>ln(x)<br>log(x)<br>log10(x)<br>piecewise(*x)<br>pow(x,y)<br>power(x,y)<br>root(n,x)<br>sec(x)<br>sech(x)	eq(*x)<br>geq(*x)<br>gt(*x)<br>leq(*x)<br>lt(*x)<br>neq(x,y)<br>plus(*x)<br>times(*x)<br>minus(x,y)<br>divide(x,y)<br>multiply(*x)

4.9 Topology file for parameter estimation

The topology file for parameter estimation is not so different from regular topology files. The only difference is that we put a negative sign (-) to the rate constant and species initial concentration that we want to estimate. See section 8.5 and 8.7 for a detailed description of topology files used in parameter estimation. Check how to set up parameter estimation properly in those sections.

5 BioSANS Basic Analysis

Now, try running “**Q4a.txt**” and see how it looks:

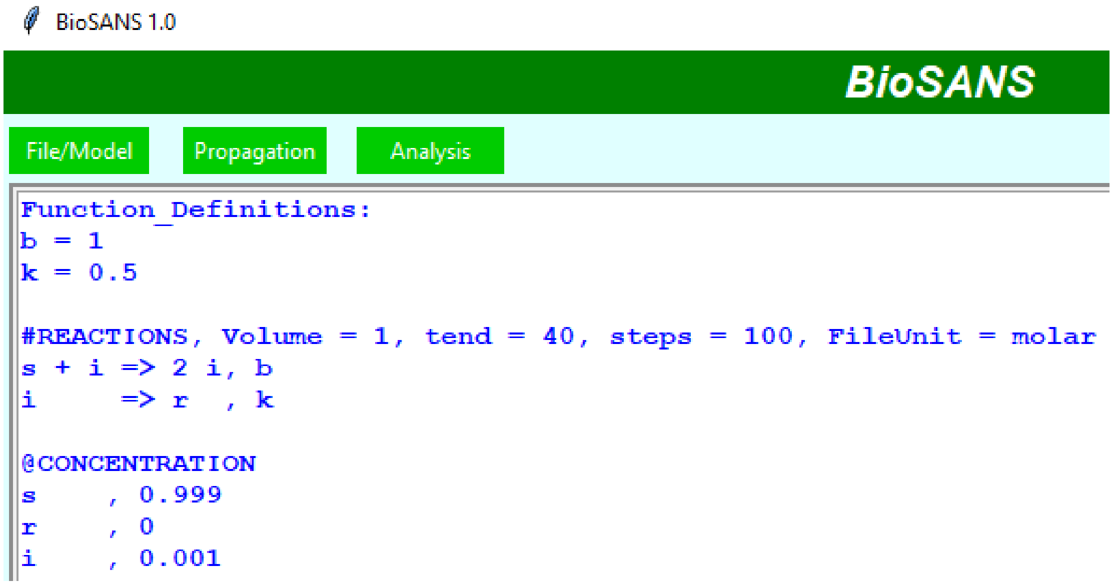

Figure 20. Topology file for Q4a.txt.

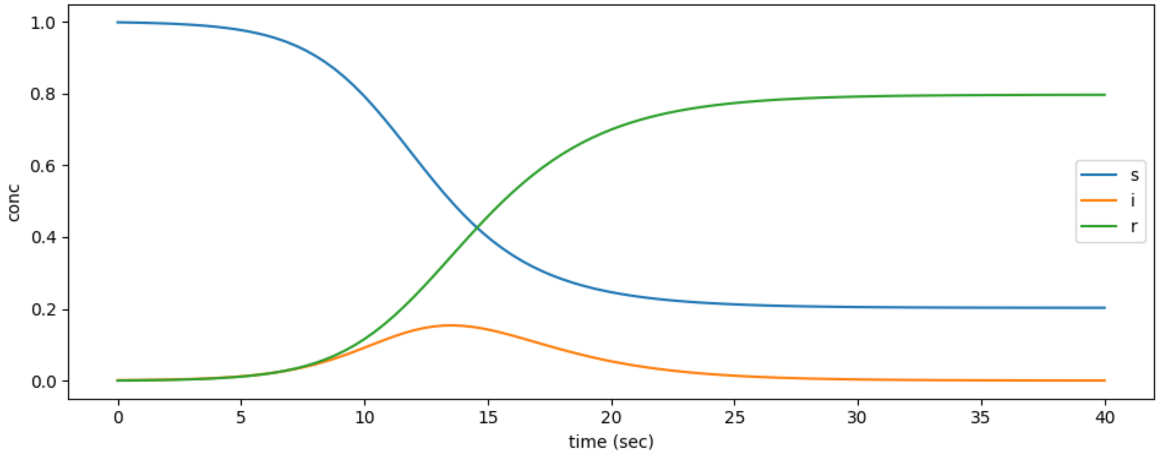

Figure 21. Plot of simulation for Q4a.txt topology file.

Opening a regular text file

Click File => Open => Topology/File => the .dat without _params

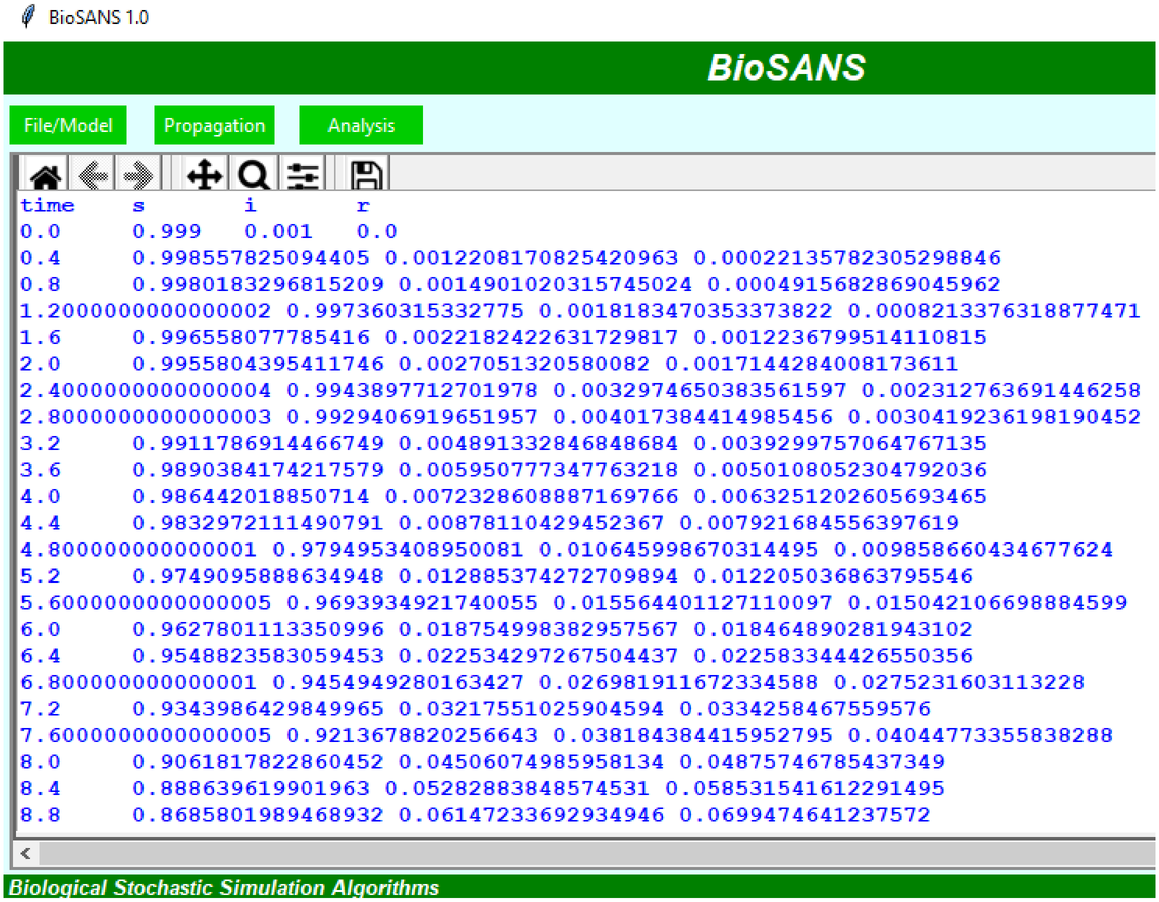

Figure 22. Trajectory tabulation for each species in Q4a.txt topology file after simulation.

Loading trajectories

1. Click File => Open => Trajectory file

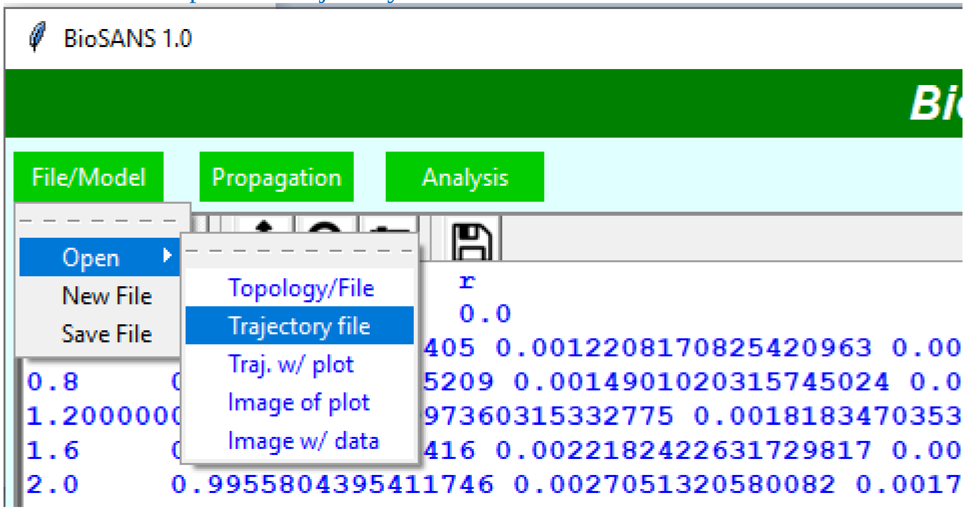

Figure 23. Loading trajectory data.

2. Choose the .dat file without the "_params"

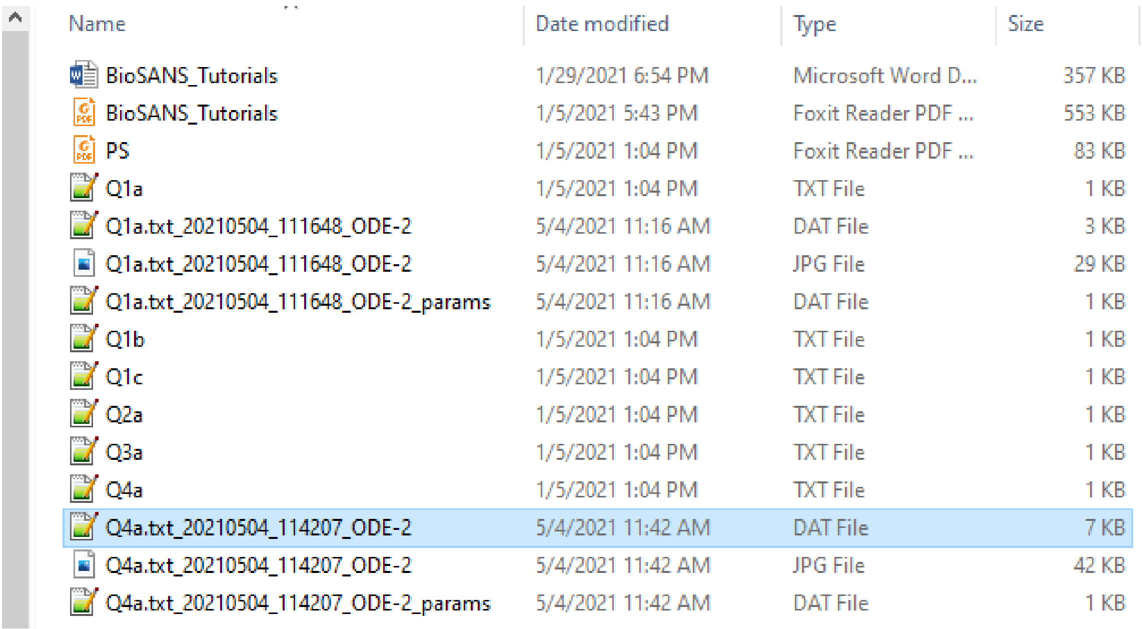

Figure 24. Selecting trajectory data to load.

Now that the trajectory data are loaded in BioSANS, and we can perform post-simulation analysis of the trajectory.

Plotting phase portrait

Click Analysis => Phase portrait

The Phase portrait selection window will appear. We can choose the axis to plot in the phase portrait.

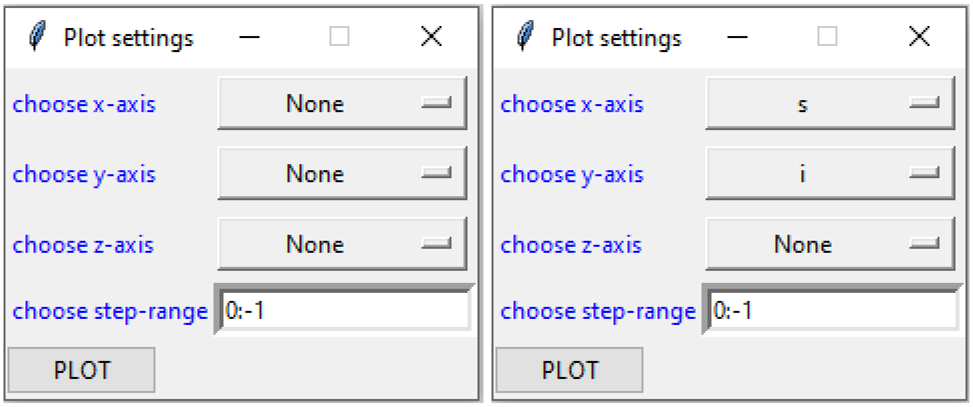

Figure 25. Phase portrait selection window.

For now, choose s and i and click “plot” to see a 2D phase portrait.

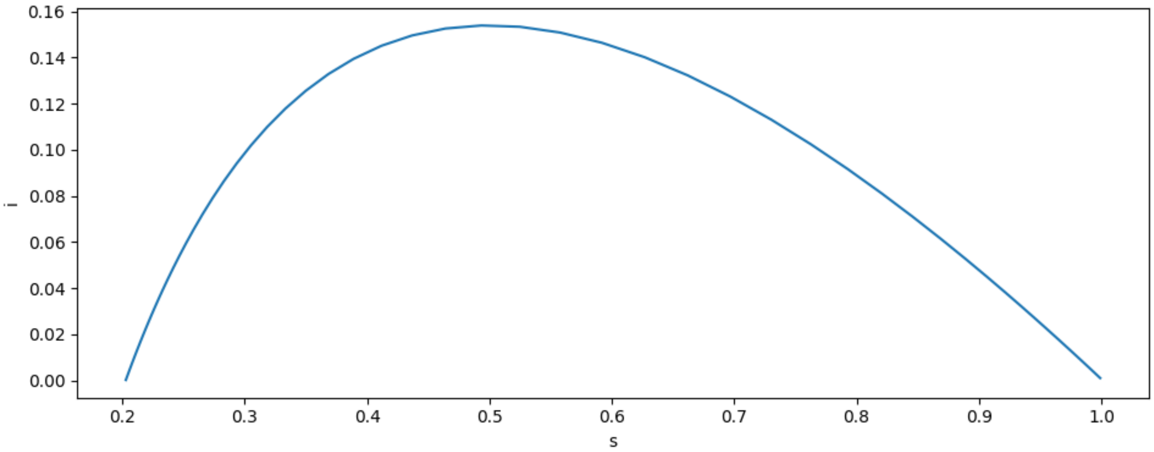

Figure 26. Phase portrait for species s and i.

We can also plot a 3D phase portrait as follows:

Plotting 3D phase portrait:

1. Close BioSANS and all plots
2. Open a new BioSANS GUI => Click Analysis => Phase portrait
3. Browse trajectory file
4. Choose species to plot
5. Click plot

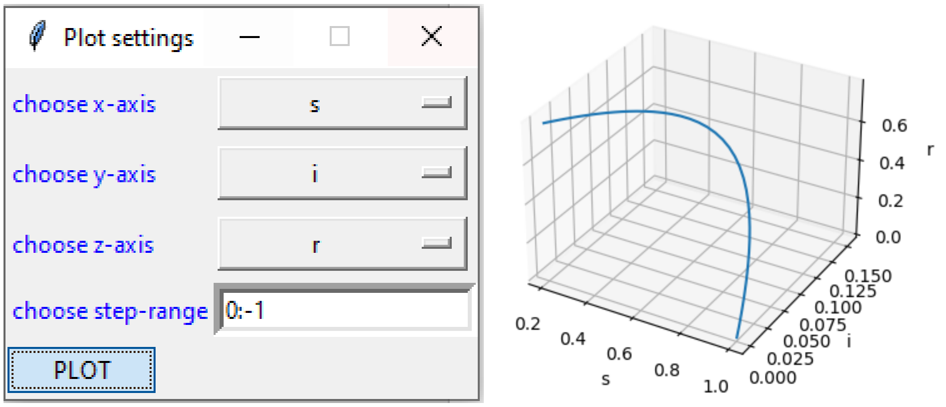

Figure 27. 3D Phase portrait for species s, i, and r.

6 BioSANS Modeling Tasks Use Cases and Workflow

This section focuses on step-by-step cases in BioSANS. BioSANS can be used via the GUI, as a Python import, or as a console interface. Assuming that all necessary libraries are installed, this section will serve as a guide to launch BioSANS and perform simple to complex simulation tasks.

A detailed description of the installation procedure is in section 1. For those who skip section 1, BioSANS can simply be installed in a Python or Anaconda terminal by using the following command.

```
pip install BioSANS2020 --upgrade
```

To launch BioSANS installed by using the above command, we need to run any one of the following commands:

```
BioSANS
python -m BioSANS2020
python -m BioSANS2020.BioSANS
python -m BioSANS2020.BioSSL    # command line version
```

6.1 GUI workflow

6.1.1 Steps to start BioSANS GUI

For Windows, Mac, and Ubuntu users who use the executable installer, double click the BioSANS shortcut created in the desktop and in start menu.

The following steps will launch BioSANS using a terminal

a. Open Anaconda or Python terminal
b. Type BioSANS and press enter

The following commands will also launch BioSANS.

a. Python – m BioSANS2020
b. Python – m BioSANS2020.BioSANS

6.1.2 Loading topology or any file

a. Click “File => Open => Topology/File” — this will open a File Browser window
b. Browse the topology or file — this will show the content of the topology or file in the BioSANS window.

6.1.3 Creating a new file

a. Click "File => New File=>Blank file" — this will open a white text area for typing.
b. Choose the Topo file and or the ODE file instead of Blank file whenever necessary. Topo and ODE files already contain the tags for those file types.
c. Type any text in the blank file. It can be a Python script. For the topo file, complete the model in each tag. For the ODE file, type the ODE descriptions.
d. Click the middle mouse button — this will open a save file window.
e. Write the file name and click save.

6.1.4 Saving file

a. Click "File => Save File" — this will open a save file window.
b. Write the file name and click save.

6.1.5 Closing files

a. Click the red x: button in the upper right corner of the internal window

6.1.6 Modifying files (i.e., topology files)

a. For any file opened by "File => Open => Topology/File => Browse File", we can edit its contents by typing new contents, deleting, modifying etc.
b. Save the file using "File => Save File" or by clicking the middle mouse button.
c. Write the file name and click save.

6.1.7 Symbolic analytical expression for chemical species

The symbolic computation feature in BioSANS is described in detail in section 10.1.1. Here we present the steps to perform symbolic computation using the GUI.

a. Load topology files using "File => Open => Topology/File => Browse File"
b. Click "Propagation/Analysis => Analytical soln. => Choose one of the modes"
    I. Pure symbolic: f(t, xo, k) — expression is a function of time initial, concentration and rate constant
    II. Semi symbolic: f(t, xo) — expression is a function of time and initial concentration
    III. Semi symbolic: f(t, k) — expression is a function of time and rate constant
    IV. Semi symbolic: f(t) — expression is a function of time
    V. For wxmaxima — this will create codes to run symbolic computation in wxmaxima. Users need to copy the codes, paste it to wxmaxima, and run it there. This may give a better analytical form.

6.1.8 Deterministic integration

A full description of deterministic integration algorithms use can be found in section 10.2.1. The following steps are BioSANS GUI steps to use the algorithms in 10.2.1.

a. Load topology files using "File => Open => Topology/File => Browse File"
b. Click "Propagation/Analysis => Any of the following five algorithms"
    i. ode_int — uses the LSODA algorithm from scipy.integrate
        - currently does not support conditional statements (events, etc.)
    ii. RK4-fix-interval — manually coded to support most SBML features
    iii. RK4-tau-adaptive — supports most SBML features
    iv. Euler-tau-adaptive-1 — fast and can handle not stiff to moderately stiff cases
    v. Euler-tau-adaptive-2 — slower but can handle stiff cases
c. Choose among the following concentration options
    i. Molecule — concentration will be converted to molecule before integration
    ii. Molar — concentration will be converted to molar before integration
    iii. Mole — concentration will be converted to mole before integration
d. Change settings as needed
    i. File name — no need to change
    ii. Number of iterations — normally no need to change for deterministic case For stochastic case this serves as the number of trajectories.
    iii. File units — this is what the program uses to know how to perform unit conversion in letter c. We need to select between
        1. Molecule — topology file has molecular units
        2. Molar — topology file has molar units
        3. Mole — topology file has mole units
    iv. Volume — type the volume to use in simulation
    v. End time — the end time in the propagation
    vi. tau-scaler — modifier of time step (it is the ***f*** in the algorithm section of this supplementary information)
    vii. Normalized — if True, the y axis will be normalized based on max value
    viii. logx — if True, the x-axis will be in log scale
    ix. logy — if True, the y-axis will be in log scale
    x. method — this is preselected based on what the user selects in b.
    xi. tsteps — number of steps reported in the final result
    xii. mix_plot — if True, all species are plotted in one frame
    xiii. save — if True, the trajectory file will be saved as a file
    xiv. outfname — directory of topology file + the filename of the output. The user can change the last word after the last "/" to any filename.
    xv. showplot — if True, this will plot the result of the simulation. It also creates an image of the plots in the directory of the topology file
    xvi. time label — allows for changing the x-axis label of the plot.
    xvii. Cini range — allows for changing the range of initial concentration for a particular species. For example, typing "A, 5, 10, 15, 20", will initiate the simulation at varying A with concentrations of 5, 10, 15, 20.

xviii. K-range — allows for changing the rate constant range. For example, to change the top-most forward reaction, the user can type something like “kf1, [0.01, 0.02, 0.03]”. This will use values 0.01, 0.02, and 0.03 for the forward rate constant in the first reaction in topology. Similarly, we can also do this for kb, (i.e., “kb2, [0.01, 0.02, 0.03]”), which will vary the backwards rate constant of reaction 2 in the topology. We can vary many rate constants for using BioSANS as a library or in console. The k-range has the following inputs for varying many rate constants: [[r1,r2],x], where r1 is the list of row numbers, r2 is a list of 0 or 1, where the forward reaction is 0 and the backward reaction is 1, and x is a list or rate constant.

Example: [[[0,2], [0,1]], [0.1, 0.2]] which means kf1 = 0.1, kb1 = 0.2

xix. multiproc — if True, it will use multiprocessing (useful for stochastic case)

xx. Implicit — if True, it will give fixed-interval output of integration

e. Click RUN — this will perform integration, plotting, saving results to file, etc.

6.1.9 Stochastic integration

Stochastic integration is discussed in section 10.2.4. The following steps show how to use stochastic algorithms with BioSANS GUI.

a. Load topology files using “File => Open => Topology/File => Browse File”
b. Click “Propagation/Analysis => Any of the following 5 algorithms”
    I. CLE (tau-adaptive) — performs our version of tau-adaptive chemical Langevin propagation
    II. CLE (fix-Intvl) — performs regular chemical Langevin propagation
    III. Tau-leaping — uses Yang Cao’s tau-leaping algorithm implemented on 3 different ways
        1. Tau-leapingV1 — pure tau-leaping
        2. Tau-leapingV2 — swapping between Gillespie and tau-leaping
        3. Sim-TauLeap — with our modification
    IV. Gillespie — Exact algorithm. Makes use of the direct method.
c. Change settings as needed. It follows the same setting choices in 6.1.8 d
    I. Number of iterations — tells how many trajectories to run
    II. multiproc — change this to True for faster simulation
d. Click RUN — this will perform integration, plotting, saving results to file, etc.

6.1.10 Linear noise approximation (LNA)

Sections 10.1.2 and 10.2.2 show the algorithmic details of symbolic and numeric LNA. The following steps show how to use LNA in BioSANS GUI.

a. Load topology files using “File => Open => Topology/File => Browse File”
b. Click “Propagation/Analysis => Linear Noise Appx.” and choose among the following options:

I. Numeric values — solves LNA using the solve_continuous_lyapunov function from SciPy. It follows the following form AC + CA.T + BB.T = 0. Deterministic trajectory is initially solved up to a point close to steady state. The nullspace of the ODE is then calculated. Lyapunov form is the set up for the solve_continuous_lyapunov function.
II. Symbolic, Microscopic — uses microscopic equations. Symbolically solves LNA using SymPy. The variables are first manipulated to AC + CA.T + BB.T = 0 form before we can solve the covariance matrix.
III. Symbolic, Macroscopic — uses macroscopic equations. Symbolically solves LNA using SymPy and reports solutions as a function of initial concentration and rate constant.
IV. Symbolic, f(xo), Macroscopic — report solutions as a function of initial concentration
V. Symbolic, f(ks), Macroscopic — report solutions as a function of rate constant
VI. Symbolic, values, Macroscopic — report solution as numerical values
VII. COV-time-dependent — time-dependent covariance matrix with plots
VIII. FF-time-dependent — time-dependent Fano-Factor with plots

6.1.11 Network localization

a. Load topology files using "File => Open => Topology/File => Browse File"
b. Click "Propagation/Analysis => Network Localization." and choose among the following options:
   I. Symbolic, Macroscopic — solve network sensitivity matrix and report answer in analytical form (full details are in section 10.1.3)
   II. Numeric, Macroscopic — solve network sensitivity matrix numerically (refer to 10.1.3)

6.1.12 Parameter estimation

The details of parameter estimation are discussed in section 10.2.3.

a. Load topology files using "File => Open => Topology/File => Browse File"
   There is a main difference between topology files for parameter estimation and regular topology files. For parameter estimation, we still need to indicate the estimated rate constant. For the rate constant we want to estimate, put a negative sign to our estimate. This will tell BioSANS that we want to estimate that particular rate constant. The following image is an example of a topology file for parameter estimation:

```
Function_Definitions:
Ao = 100
Bo = 10

kf1 = 0.5
kb1 = 0.3

#REACTIONS
2 A <=> B, -kf1, -kb1

@CONCENTRATION
A, Ao
B, Bo
```

The negative sign on kf1 and kb1 tells BioSANS that we want to estimate their values. Although we put some value for kb1 and kf1, they will not be used but are just there as a placeholder.

b. Click “File/Model => Estimate Params.” and choose among the following options:
   i. Nelder Mead (NM), Macroscopic — uses SciPy Nelder-Mead algorithm
   ii. Nelder Mead (NM), Microscopic
   iii. Powell, Macroscopic — uses SciPy Powell algorithm
   iv. Powell, Microscopic
   v. L-BFGS-B, Macroscopic — uses SciPy L-BFGS-B algorithm
   vi. L-BFGS-B, Microscopic
   vii. NM-Diff. Evol., Macroscopic — combined Nelder-Mead and differential evolution algorithm
   viii. NM-Diff. Evol., Microscopic
   ix. Parameter slider/scanner — GUI to manually change parameter by slider and compare to actual data
   x. MCEM, Macroscopic — Our implementation of MCEM algorithm
   xi. MCEM, Microscopic
c. After selecting the algorithm, browse the file containing the species trajectory data. It should be tab-delimited with the header of species using the names of species in the topology file. The first column is time, the second column and so on are for species concentration at that particular time. The following image is an example of the data file:

```
time     A        B
0.0      100.0    0.0
0.25     88.24969025197632 11.750309748023732
0.5      77.88007831231087 22.119921687689185
0.75     68.72892784164061 31.27107215835944
1.0      60.65306592491437 39.346934075085684
1.25     53.526142785532 46.473857214468055
1.5      47.236655135816875 52.763344864183218
1.75     41.68620193454698 58.31379806545308
2.0      36.78794415253036 63.21205584746969
2.25     32.46524678349081 67.53475321650924
2.5      28.650479737056997 71.349520262943060
```

Although the ideal case is that we know all species trajectories, in BioSANS we can still perform parameter estimation even if only a few species are available. The species label should match that in the topology file.

6.1.13 Post-processing/analysis

a. For post-processing, we have to do simulation first then we can load the trajectory file of the simulation result in any of the following ways:
   I. "File => Open => Trajectory file" — loads trajectory data in memory
   II. "File => Open => Traj. w/ plot" — loads trajectory data and plots it
   III. "File => Open => Image w/ data" — loads trajectory data and image of plot
b. Browse the trajectory file or the image of the trajectory plot. Select the file and click Open
c. Click "Analysis/post proc => Select among the following options:"
   I. Covariance — calculates covariance for stochastic trajectories
   II. Fano-Factor — calculate fano-factor for stochastic trajectories
   III. Cross-correlation — calculate cross-correlation for stochastic trajectories
   IV. Probability density — calculate probability density for stochastic trajectories
   V. Frequency distribution with respect to time — calculator time-dependent frequency distribution
   VI. Histogram slice of time — gives a histogram slice of time
   VII. Average of trajectory — takes the mean of all stochastic trajectories
   VIII. Phase Portrait — allows plotting species vs. species in 2d and 3d
      1. choose x-axis — the species for x-axis
      2. choose y-axis — the species for y-axis
      3. choose z-axis — optional species for z-axis
      4. choose step-range — 0:-1 means all data. A user may put N:-1, which means the first N time points are not used. N can be any integer less than the total number of time points.
   IX. Plot Data — allows for selecting what to plot as a function of time
      1. Put a check in the species to include them in plotting.
      2. Click plot

6.2 BioSANS as a Python import

Python experts can take advantage of BioSANS by importing it as a library. To use BioSANS in Python, we need to import the biosans_lib.py module. For stochastic simulation the proc_global.py module is also needed. An example import set-up to start basic simulation is shown in **Error! Reference source not found.** with parameters defined in **Figure 29**.

```
# proc_global is needed only for stochastic simulation
from BioSANS2020.myglobal import proc_global
from BioSANS2020 import biosans_lib as biosans


# the if main is needed only for stochastic simulation
if __name__ == '__main__':

    modelA = """
        #REACTIONS
        A <=> B, 0.5, 0.3

        @CONCENTRATION
        A, 100
        B, 10
    """

    my_model = biosans. \
               model(modelA, FileIn="molecules"). \
               save_traj("AtoBrev.txt").plot()

    proc_global.init(proc_global)
    data = my_model.run(method="Gillespie_", ntraj=30, mult_proc=True )
    my_model.clean()    # delete intermediate files generated
```

Figure 28. An example of using BioSANS as a library.

Additional examples are provided in the following webpage under the BioSANS as a python import link in the left-hand side of the page.

https://efajiculay.github.io/SysBioSoft/

```
.model(self, topo=None, sbml=None, FileIn=None, Volume=None)

topo           #  topology file to used
sbml           #  sbml file for models written in sbml format
FileIn         #  can be "mole","molar", or "molecules" - topo file units
Volume         #  Volume used in the model


The following can be appended to model defined above


.data(self, exp_data_file=None)

exp_data_file  #  Experimental data or given data

.plot(self, normalize=False, mix_plot=True, logx=False, logy=False)

normalize      #  If True, the y axis will be normalized based on max value
mix_plot       #  If True, all species are plotted in one frame
logx           #  if True, the x-axis will be in log scale
logy           #  if True, the y-axis will be in log scale

.save_traj(self, out_fname=None)

out_fname      #  output filename appended to output files

.extra(self, c_input={}, vary="")

c_input        #  No longer supported
vary           #  Varying initial concentration

.clean(self)


The .run() method is submitting the settings above to the process module.


.run(self, method=None, ntraj=None, tend=None, step_size_scaler=None,
            steps=None, mult_proc=None, implicit=True, cpu_mult=0.9)

method            #  A list of all available method keywords is listed below
ntraj             #  Number of iteration for stochastic integration
tend              #  end time of simulation
step_size_scaler  #  f -factor for modifying time steps
steps             #  number of steps reported in the final result
mult_proc         #  If True, it will use multiprocessing
implicit          #  True means report in time intervals similar to the
                  #  input time intervals



# The method keyword inside .run() is the key to perform all kinds of
simulation that BioSANS can provide
```

Figure 29. Parameter definitions in process call.

Several examples of using BioSANS as a Python import are shown in the following github link:

https://github.com/efajiculay/BioSANS_installers/tree/main/BioSANS_as_python_library

6.2.1 List of method keywords

The following are the list of method keywords in BioSANS. The unit label molar, molecules, and mole are the units that method will be working on. If the file unit is different, BioSANS will convert the units. The sub-labels micro and macro pertain to the equations used, which are either microscopic or macroscopic. The microscopic equation uses molecule number in the propensity expressions (Gillespie-like propensity), whereas the macroscopic uses deterministic propensity expressions (similar to regular elementary ODE per molecularity).

Stochastic (refer to section 10.2.4)

1. "CLE" - Molecules(micro), tau-adaptive
2. "CLE2" - Molecules(micro), cle-fixIntvl
3. "Gillespie_" - Molecules(micro), Direct method
4. "Tau-leaping" - Molecules(micro), Not swapping with Gillespie
5. "Tau-leaping2" - Molecules(micro), Swapping with Gillespie
6. "Sim-TauLeap" - Molecules(micro), Simplified, Swapping with Gillespie

Deterministic (refer to section 10.2.1)

7. "Euler-1" - Molecules(micro), tau-adaptive-1
8. "Euler-2" - Molar (macro), tau-adaptive-1
9. "Euler-3" - Mole (macro), tau-adaptive-1
10. "Euler2-1" - Molecules(micro), tau-adaptive-2
11. "Euler2-2" - Molar (macro), tau-adaptive-2
12. "Euler2-3" - Mole (macro), tau-adaptive-2
13. "ODE-1" - Molecules(micro), using ode_int from SciPy
14. "ODE-2" - Molar(macro), using ode_int from SciPy
15. "ODE-3" - Mole(macro), using ode_int from SciPy
16. "rk4-1" - Molecules(micro), fix-interval
17. "rk4-2" - Molar(macro), fix-interval
18. "rk4-3" - Mole(macro), fix-interval
19. "rk4-1a" - Molecules(micro), tau-adaptive
20. "rk4-2a" - Molar(macro), tau-adaptive
21. "rk4-3a" - Mole(macro), tau-adaptive

LNA (refer to 10.1.2 & 10.2.2)

22. "LNA" - Numeric, values

23. "LNA-vs" - Symbolic, values, Macroscopic
24. "LNA-ks" - Symbolic, f(ks), Macroscopic
25. "LNA-xo" - Symbolic, f(xo), Macroscopic
26. "LNA2" - Symbolic, f(xo,ks), Microscopic
27. "LNA3" - Symbolic, f(xo,ks), Macroscopic
28. "LNA(t)" - COV-time-dependent, Macroscopic
29. "LNA2(t)" - FF-time-dependent, Macroscopic

Network Localization (refer to 10.1.3)

30. "NetLoc1" - Symbolic, Macroscopic
31. "NetLoc2" - Numeric, Macroscopic

Parameter estimation (refer to 10.2.3)

32. "k_est1" - MCEM, Macroscopic
33. "k_est2" - MCEM, Microscopic
34. "k_est3" - NM-Diff. Evol., Macroscopic
35. "k_est4" - NM-Diff. Evol., Microscopic
36. "k_est5" - Parameter slider/scanner
37. "k_est6" - Nelder-Mead (NM), Macroscopic
38. "k_est7" - Nelder-Mead (NM), Microscopic
39. "k_est8" - Powell, Macroscopic
40. "k_est9" - Powell, Microscopic
41. "k_est10" - L-BFGS-B, Macroscopic
42. "k_est11" - L-BFGS-B, Microscopic

Symbolic/analytical expression of species (refer to 10.1.1)

43. "Analyt" - Pure Symbolic :f(t,xo,k)
44. "Analyt-ftx" - Semi-Symbolic :f(t,xo)
45. "SAnalyt" - Semi-Symbolic :f(t)
46. "SAnalyt-ftk" - Semi-Symbolic :f(t,k)
47. "Analyt2" - Creates commands for wxmaxima

6.3 BioSANS console interface - structured simulation language (SSL)

Our console interface is similar to MySQL in terms of readability and ease of use but is currently of limited capability. It can be launched from a Python terminal or from the BioSANS GUI.

From the terminal, BioSSL can be launched as follows:

1. Open Anaconda/Python terminal
2. Activate conda environment for BioSANS if it was created during installation. Type “conda activate BioSANS” and press enter
3. Type “BioSSL” and press enter

4. As an alternative, the user can type “python –m BioSANS2020.BioSSL” and press enter

From the BioSANS GUI, go to “File/Model” and select “Run SSL”.

The scripts of **Figure 30** are an example of the set of commands that we can run in BioSSL. ***Each command in BioSSL ends with* “;”**. To quit BioSSL type **quit()** and press enter.

```
propagate
    A => B, 0.2 &
    B => C, 0.3
where
    A=100 &
    B=0.2 &
    C=0
using CLE
with
    tn=50 &
    tlen=1000 &
    miter=30 &
    mult_proc=True &
    fout=Traj1
;

propagate A => B, 0.2 & B => C, 0.3 where A=100 & B=0.2 & C=0 using CLE
with tn=50 & tlen=1000 & miter=2 & mult_proc=True & fout=Traj2;
```

Figure 30. BioSSL commands example. The first one is multiple lines, and the second one is inline.

6.3.1 List of currently supported commands:

1. ls - list and prints the contents of the current directory.
2. cd - change directory
3. pwd - print current working directory
4. open_pwd - open current working directory using default file browser
5. mkdir <dirname> - create new directory. For example

    mkdir MyFolder:
6. propagate <rxn> where <ini condition> using <method> with <additional constraint> — propagate a trajectory. An example is shown above in **Figure 30**.
7. load <SSL file> — load and run SSL file
8. SSL file is a text file containing SSL commands. For example, the commands in **Figure 30** can be saved into a file named **mycommand.txt**. To run that file, we can type

    load example1.txt;
9. read_traj <traj. file> as <variable> — save trajectory data into a variable. This command is best suited ***if the user wants to perform numerical stuff*** in the data. For example;

    read_traj example1.out_CLE.dat as data;
10. pdread_traj <traj. file> as <variable> — save trajectory data into a variable. This command is best suited ***if the user wants to plot the data or print the data nicely***. It makes use of pandas. For example:

    pdread_traj example1.out_CLE.dat as data;
11. print <variable> — print the data read from the trajectory file using pdread_traj or read_traj. For example, if A, B, C is in trajectory read by pdread_traj or read_traj;

print data A B C:

12. plot <variable> <species> — plot the data read from the trajectory file using pdread_traj. For example,

    plot data A B; **-** plot the phase portrait A vs. B
    plot data time A; **-** plot A vs. time
    plot data time A B; **-** plot A and B vs. time

13. calc_covariance <variable> <number of points> — calculate covariance of the data read from the trajectory file using read_traj. For example,

    calc_covariance data 100; — calculate cov from the last 100 time points

14. prob_density <variable> — create probability density plots of the data read from the trajectory file using read_trajdata. For example,

    prob_density data;

15. calc_average <variable> — calculate average of the data read from the trajectory file using read_traj. For example,

    calc_average data;

16. prob_density_wtime <variable> — calculate time-dependent probability density of the data read from the trajectory file using read_traj. For example,

    prob_density_wtime data;

17. quit() – exit BioSSL. ***This command doesn't need* "; "**

    quit()

The list of possible options after the "with" keyword are the following with their defaults:

tn = 100, Vol = 1.0, tsc = 1.5, tlen=100, mult_proc = False, fout = "temp_traj", fileUnit = "molecules", plot = True, mixp = True, norm = False, logx = False, logy = False, EdataFile = None, topo=None. The tsc value if the tau-scaler and the EdataFile is the experimental data for trajectory in tab delimited format. See additional examples in section 12.

7 List of Codes in BioSANS

This section describes the codes in BioSANS. The tree structure of BioSANS2020 is presented in **Figure 31**. When using BioSANS as a Python import, this tree may help as a guide on how to construct the import especially if a user wants to grab specific functions from each module. For a detailed description of each algorithm, please see the algorithms section of this manual.

7.1 Hierarchical tree structure of BioSANS

```
BIOSANS2020
│   BioSANS.py
│   BioSSL.py
│   LICENSE
│   RunBioSANS.py
│   tree.dat
│   __init__.py
│   __main__.py
│
├───analysis
│   │   __init__.py
│   │
│   ├───numeric
│   │   │   sample_points.py
│   │   │   Transform.py
│   │   │   __init__.py
│   │
│   ├───plotting
│   │   │   plot_traj.py
│   │   │   __init__.py
│
├───cli_functs
│   │   ssl_calls.py
│   │   __init__.py
│
├───examples
│       VCGN.py
│       __init__.py
│
├───gui_functs
│   │   draw_figure.py
│   │   prepare_canvas.py
│   │   scrollable_text.py
│   │   __init__.py
│
├───math_functs
│   │   sbmlMath.py
│   │   __init__.py
│
├───model
│   │   new_file.py
│   │   topology_view.py
│   │   __init__.py
│   │
│   ├───ode_parse
│   │   │   odeExtract.py
│   │   │   __init__.py
│   │
│   ├───param_est
│   │   │   myMCEM.py
│   │   │   param_estimate.py
│   │   │   param_slider.py
│   │   │   __init__.py
│
├───myglobal
│   │   mglobals.py
│   │   proc_global.py
│   │   __init__.py
│
├───prepcodes
│   │   process.py
│   │   processes_hub.py
│   │   __init__.py
│
├───propagation
│   │   create_wxmaxima_command.py
│   │   NetworkLoc.py
│   │   propensity.py
│   │   recalculate_globals.py
│   │   __init__.py
│   │
│   ├───deterministic
│   │   │   Euler.py
│   │   │   LNAapprox.py
│   │   │   LNAfunctionOfTime.py
│   │   │   ode_int.py
│   │   │   runge_kutta4.py
│   │   │   __init__.py
│   │
│   ├───stochastic
│   │   │   Gillespie.py
│   │   │   Gillespie_new.py
│   │   │   mystiffcle.py
│   │   │   mytauleap.py
│   │   │   sde_int.py
│   │   │   Tau_leaping.py
│   │   │   Tau_leaping2.py
```

```
|   |
|   |-------fileconvert
|   |   |   Conversion.py
|   |   |   process_sbml.py
|   |   |   __init__.py
|   |   |
```

```
|   |   |   __init__.py
|   |
|   |-------symbolic
|   |   |   Analytical_sol.py
|   |   |   LNAapprox2.py
|   |   |   __init__.py
```

Figure 31. BioSANS code tree structure.

7.2 Description of codes in BioSANS

The Python codes listed in **Table 3** contain the algorithms that make BioSANS work. These codes are grouped according to **Figure 31** and each sub-package performs a specific function. GUI, CLI, and mathematical stuff have their own separate package.

Table 3. Descriptions of codes in BioSANS.

Python files	Purpose
Analytical_sol.py	For calculating analytical expression of species concentration
BioSSL.py	The structured simulation language codes
BioSANS.py	Contains the main GUI
Conversion.py	Convert topology files to SBML files
create_wxmaxima_command.py	For exporting symbolic computation problem into wxmaxima codes
draw_figure.py	For displaying plots and images
Euler.py	Integrating ODE using tau-adaptive euler (my own version) and another tau-adaptive euler
Gillespie.py	Direct method of Gillespie algorithm
LNAapprox.py	For numeric LNA
LNAapprox2.py	Symbolic LNA
mglobals.py	Header import for files needing to access, manipulate, and calculate global variables
myMCEM.py	Current parameter estimation algorithm in BioSANS
mystiffcle.py	Regular chemical Langevin equation (CLE) and my own tau-adaptive version of CLE
mytauleap.py	My modified version of tau-leaping algorithm
new_file.py	For creating new files
ode_int.py	Integrating ordinary differential equation using LSODA algorithm
param_est.py	Parameter estimation from data file (trajectory)
param_est2.py	Parameter estimation (custom function) - disabled
plot_traj.py	Loading trajectory and plotting
prepare_canvas.py	Prepare canvas from drawing plots

proc_global.py	Global processes manager
process.py	Interpret topology files, prepare stoichiometric matrix, grab rate constants, rate expressions, initial concentrations, etc. Prepare inputs for simulations.
process_sbml.py	Converting SBML files to Biostoch topology files
processes_hub.py	Serves as the center of decision where to transfer the input from process to the actual simulation
propensity.py	Calculates propensity at each iteration step for any simulation algorithm (numeric and symbolic)
runge_kutta4.py	Runge-Kutta 4 and adaptive Runge-Kutta
sample_points.py	Sampling points from trajectory
sbmlMath.py	Python equivalent of SBML function calls
scrollable_text.py	Automatically create text data input for displaying equations, solutions, results, etc.
sde_int.py	Integrating stochastic differential equations (not used/disabled)
ssl_calls.py	Data processing codes called by SSL
Tau_leaping.py	Simplified version of Yang Cao’s tau-leaping algorithm
Tau_leaping2.py	Yang Cao’s tau-leaping algorithm
topology_view	For viewing topology files
Transform.py	Data processing for Biostoch GUI

8 Deterministic Modeling Examples

https://github.com/efajiculay/BioSANS_installers/tree/main/TutorialTopoFiles

8.1 Feed-forward loop with square wave input signal (Propagation)

```
Function_Definitions:
alp  = 0.15
rx   = 0.12
ry   = 0.7
kx   = 0.5
ky   = 0.1
Yo   = 0.7
Zo   = 0
theta = lambda x,k,n : (x**n)/(x**n+k**n)

#REACTIONS
0 NONE =>  X, 0
0 NONE =>  timer_X, 1

0 NONE =>  Y, 0  ::::: lambda X, Y    : -alp*Y + rx*theta(X,kx,2)
0 NONE =>  Z, 0  ::::: lambda X, Y, Z : -alp*Z +
rx*theta(X,kx,2)*ry*theta(Y,ky,2)


@CONCENTRATION
timer_X,    0, lambda timer_X  :  0 if timer_X >= 32 else timer_X
X,          1, lambda timer_X  :  0 if timer_X >= 16 else 1
Y,          Yo
Z,          Zo
NONE, 0
```

Figure 32. Feed-forward loop.txt file provided in the tutorial

Modeling steps:

1. File => Open => Topology/File =>Select the topology "Feed_forward_loop.txt"

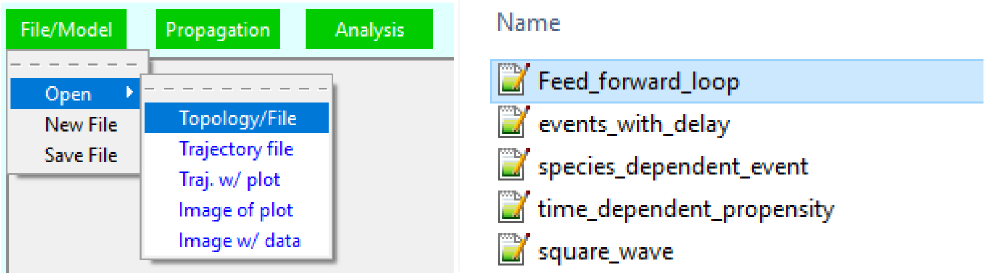

2. Propagation => RK4-fx-interval => Molar(macro)

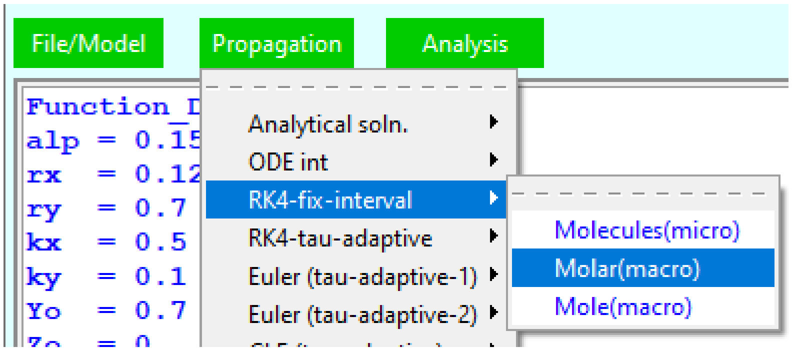

3. Change file unit to molar

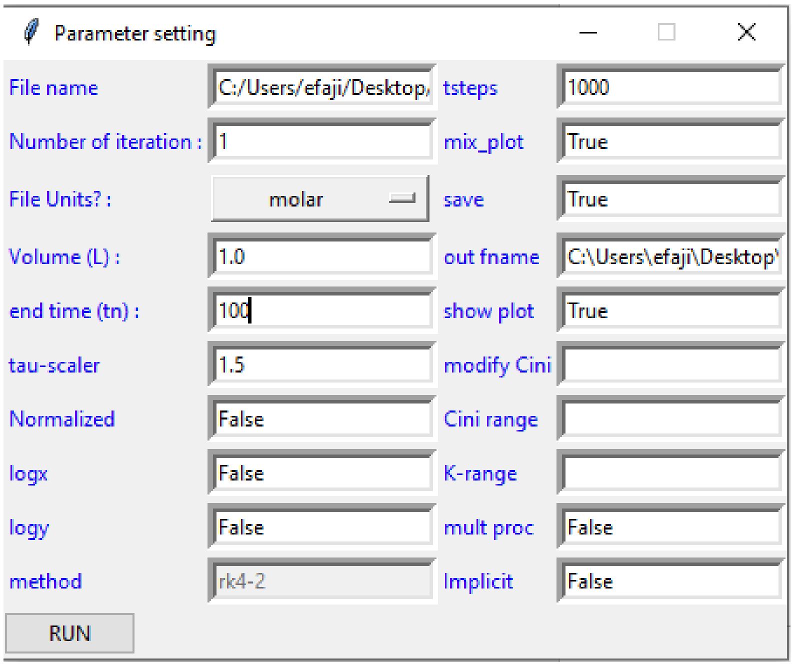

4. Click run.

The following output plot shows the square wave signal in species X and the corresponding response of species Y and Z as X varies. In **Figure 32**, "timer_X" and species "X" conditional statements handle the square wave. Species Y and Z are propagated normally, but because X is changing, they respond to X values and also oscillate.

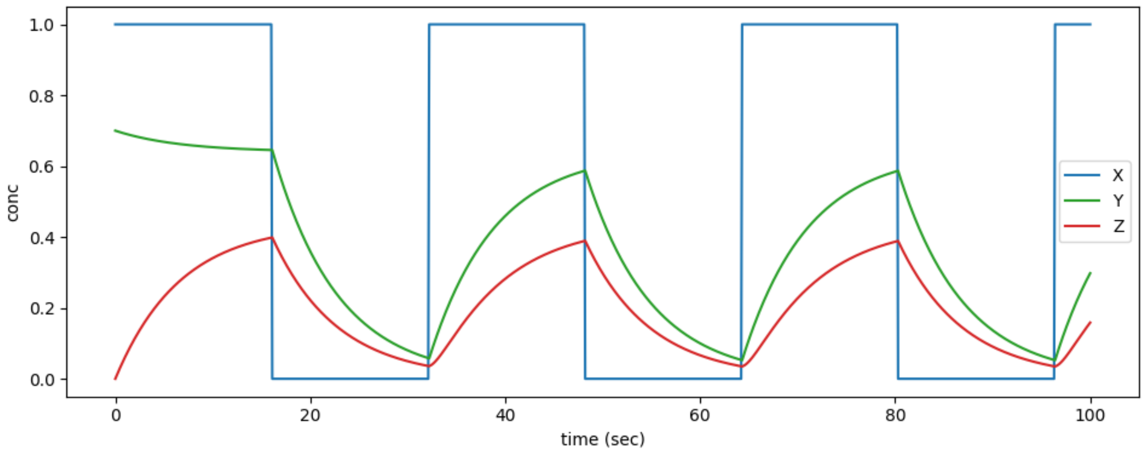

8.2 Repressilator system using Hill function (Propagation, Phase portrait)

The topology file shown in **Figure 9** is a repressilator [4] consisting of 3 proteins, **“A, B, C”,** which repress each other’s production in a loop by repressing mRNA activity. In this tutorial, we plot the limit cycle in both 2D and 3D as well as time-dependent phase portrait.

Modeling steps:

1. File => Open => Topology/File
2. Select the topology “RepressilatorWithHillFunction.txt” and click open

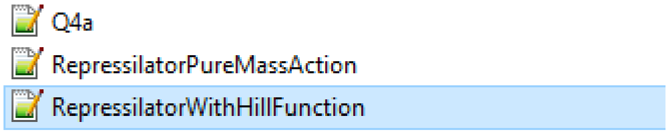

3. Propagation => ODE int => Molar(macro)
4. Change tsteps to 1000

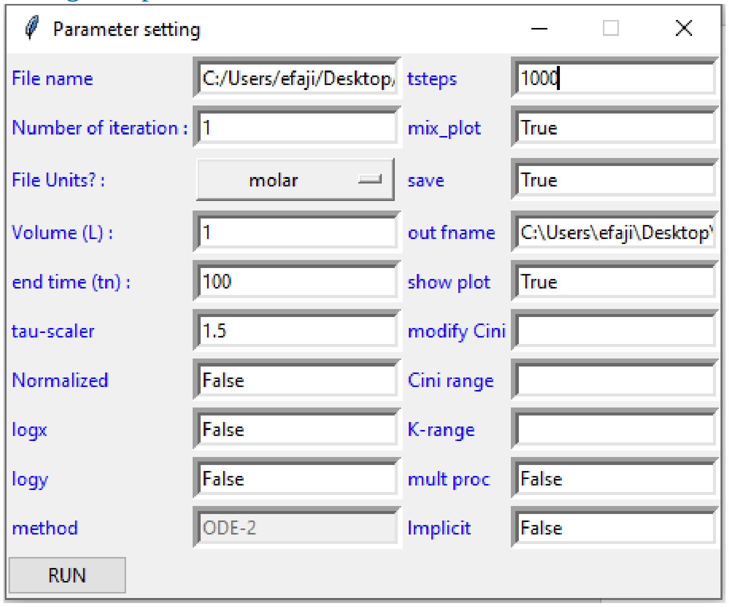

5. Click run

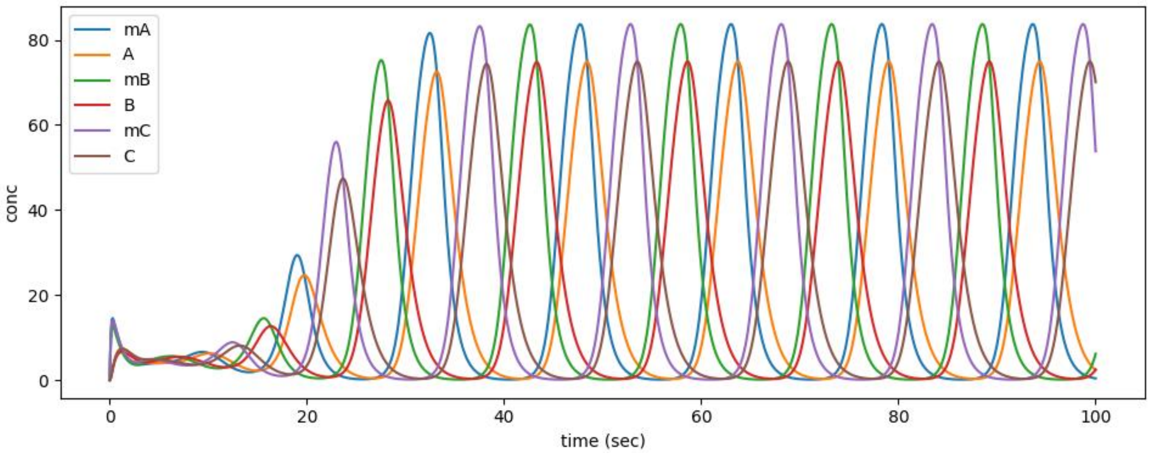

6. Load trajectory file using "Open => Trajectory file => "Repressilator…dat"

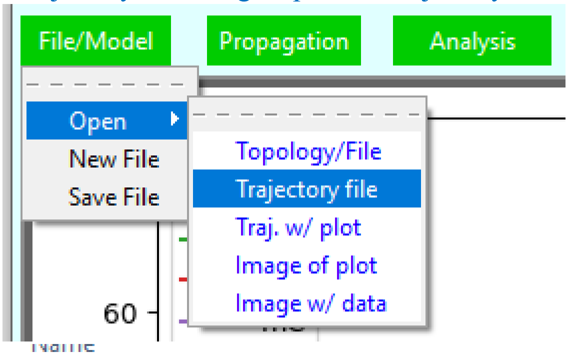

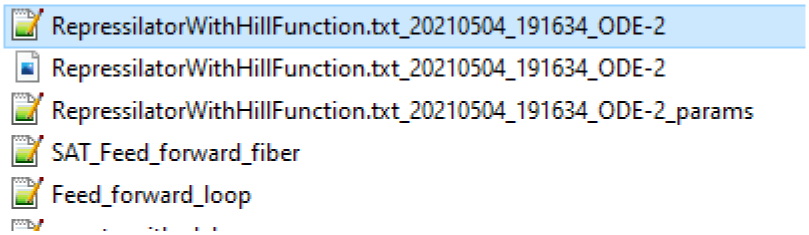

7. Click open
8. Analysis => Phase portrait
9. Choose x-axis and y-axis

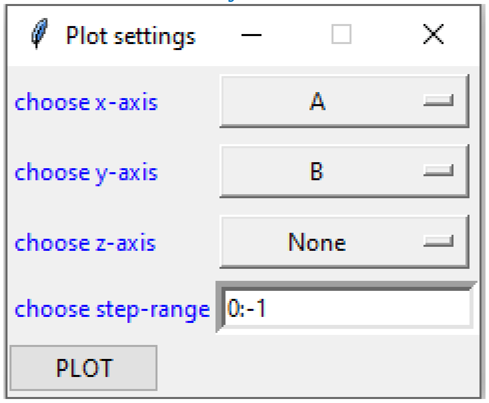

10. Click plot

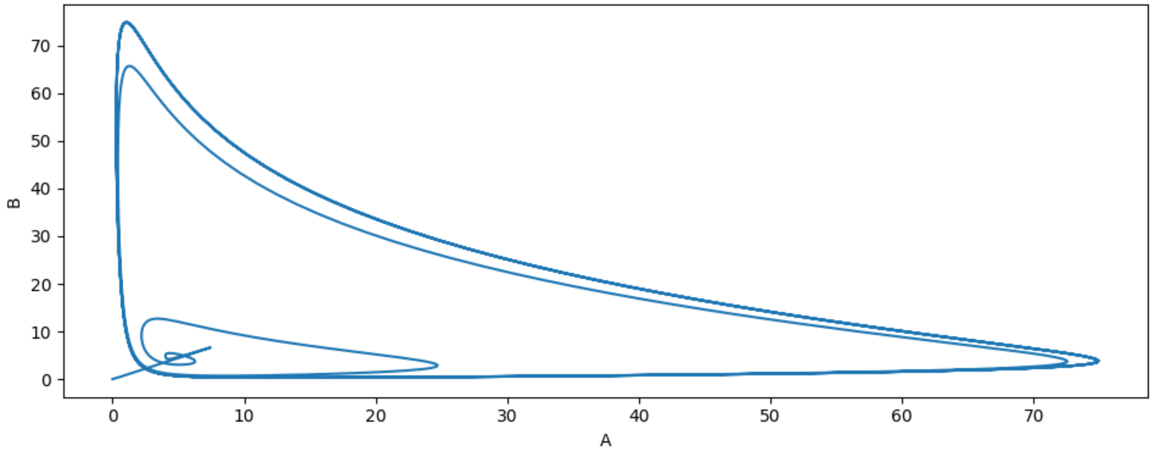

11. Change the range in the plot setting as follows, discarding first 500 time points:

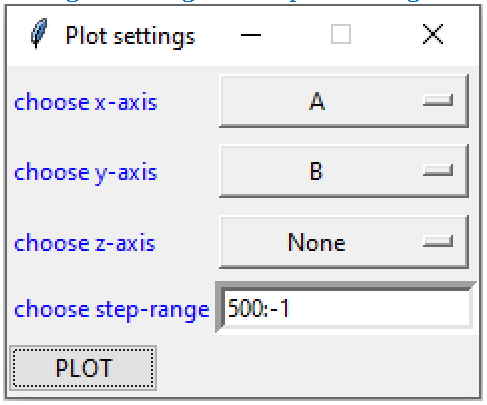

12. Click plot

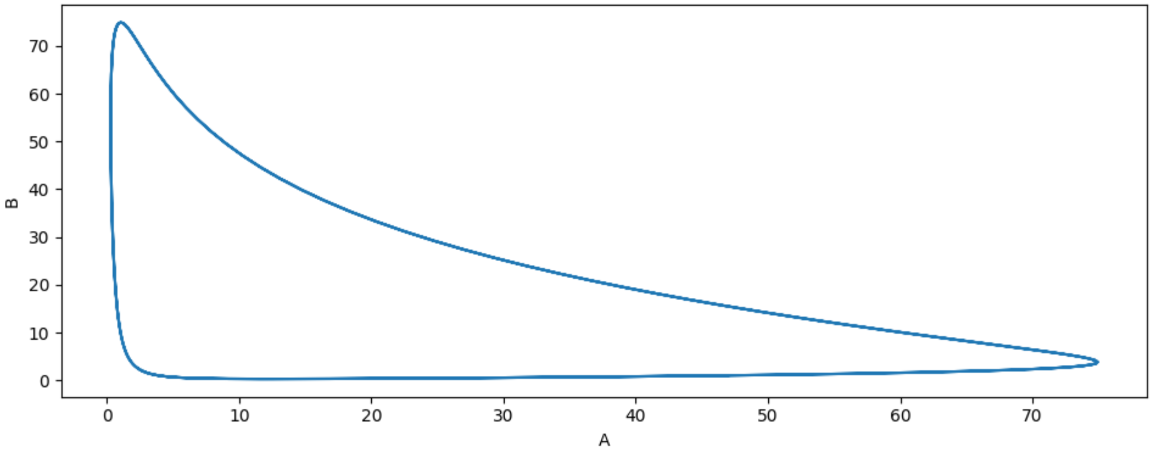

Now, try the 3D phase portrait. Before proceeding, close everything and restart BioSANS GUI. This is required for the 3D phase portrait because currently it only works for a freshly opened BioSANS window.

Modeling steps:

1. File/Model => Open => Trajectory file
2. Select the trajectory file (it is the ".dat" file without params in the name)

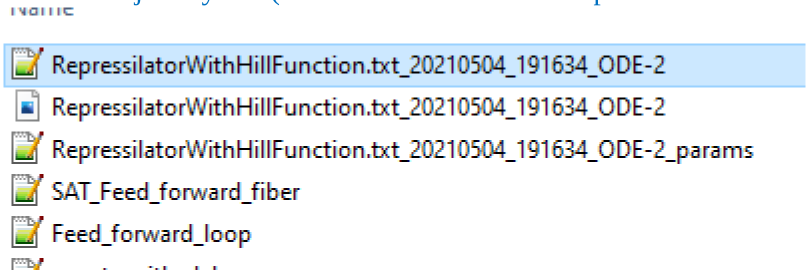

3. Click open
4. Analysis => Phase portrait
5. Select A, B, C and click plot

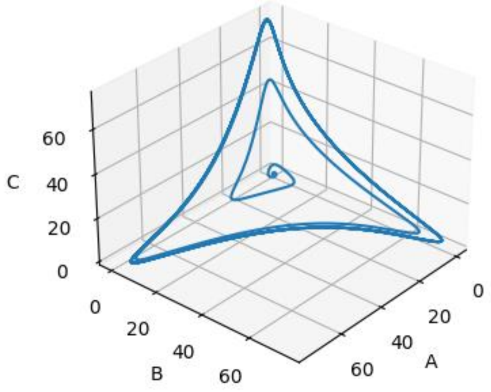

6. Close the plot and change range as follows:

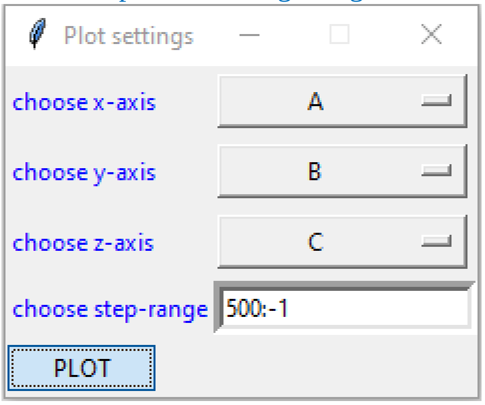

7. Click plot

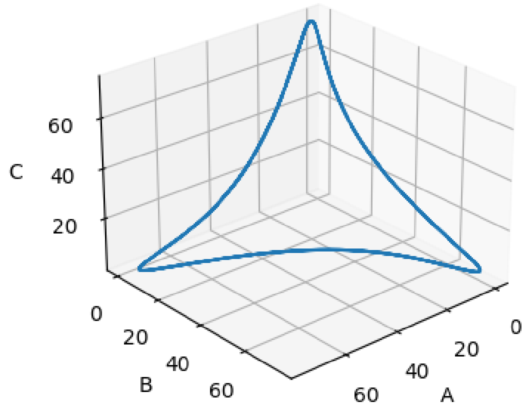

8. Close the plot and change the z-axis to time as follows:

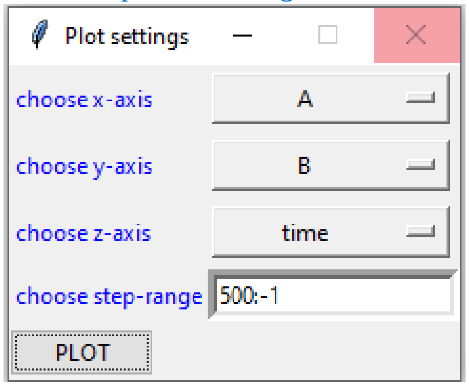

9. Click plot

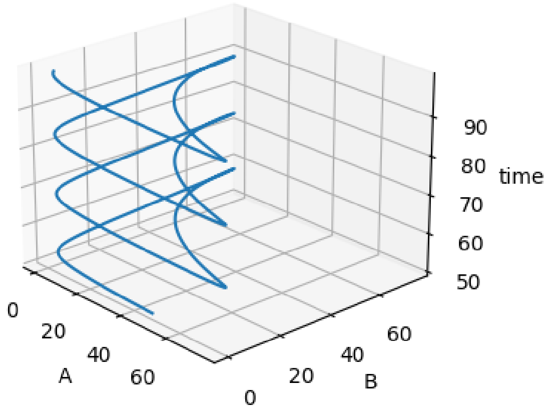

8.3 Lorenz system (Propagation, Phase portrait, 3D Phase portrait)

The Lorenz system [5] shown in
Figure 18 shows chaotic behavior because of the existence of a strange attractor. Here we model the system as for the repressilator.

Modeling steps:

1. File => Open => Topology/File

2. Select the topology “LorenzSystem.txt”

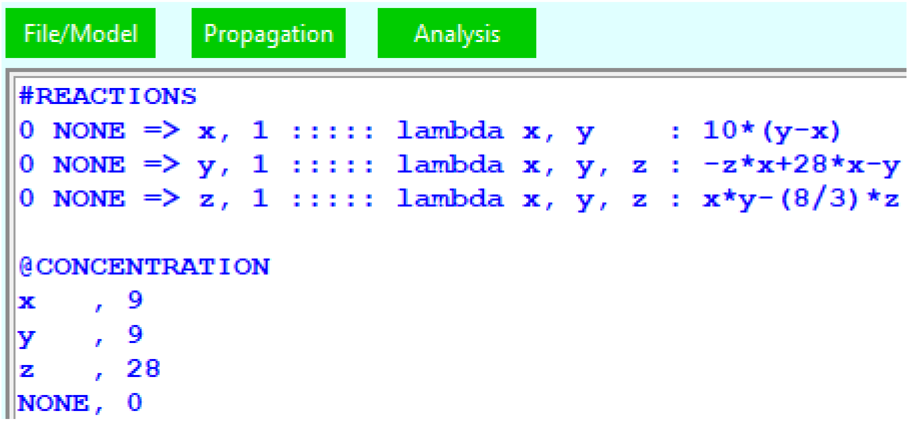

3. Propagation => ODE int => Molar(macro)
4. Change tsteps to 100000
5. Change File Units to molar
6. Click run

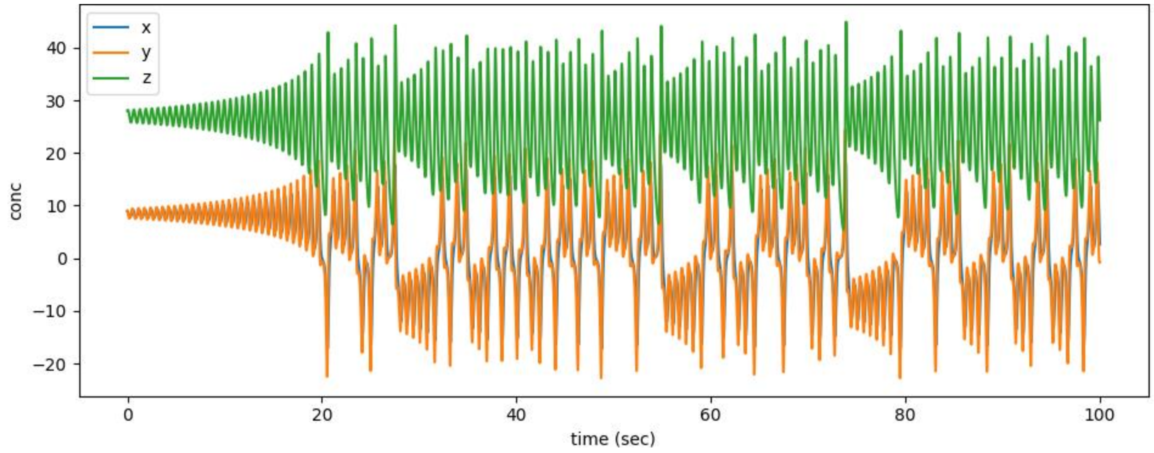

7. Change end time to 1000 and click run

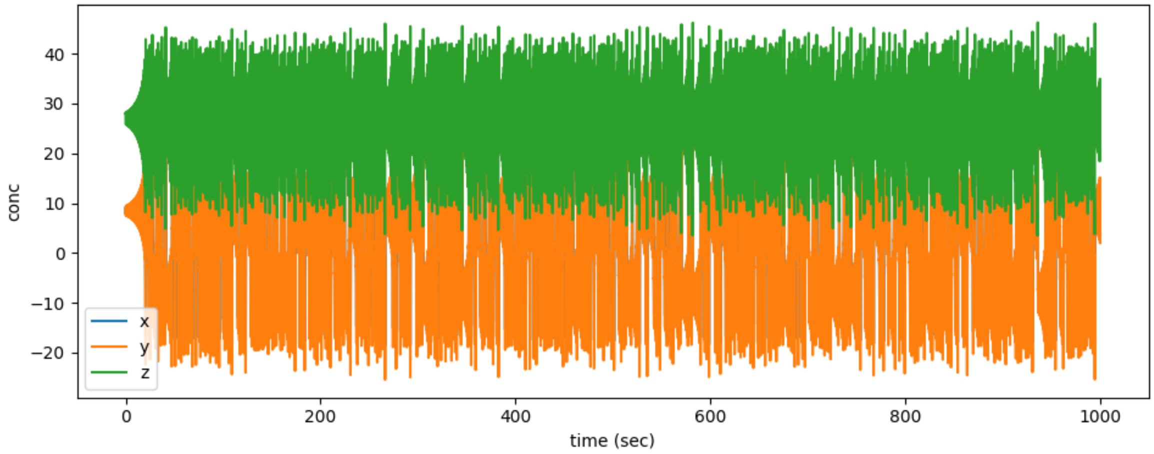

8. Close all windows associated with BioSANS
9. Start a new BioSANS window GUI

10. File/Model => Open => Trajectory file

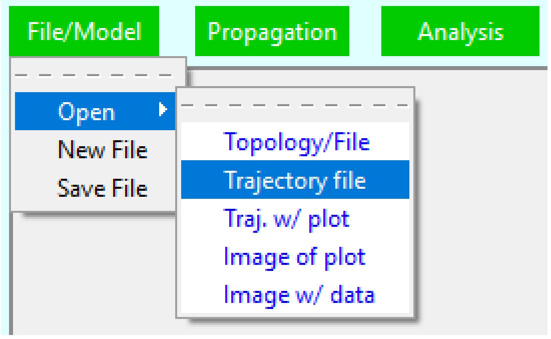

11. Select the .dat file without “params” in the filename and click open

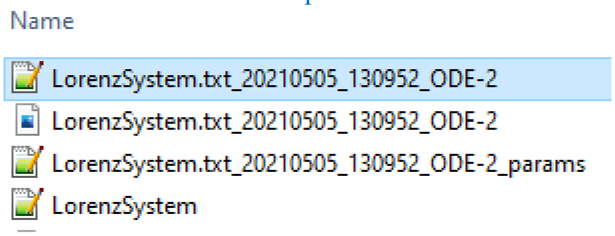

12. Analysis => Phase portrait => select x, y, z

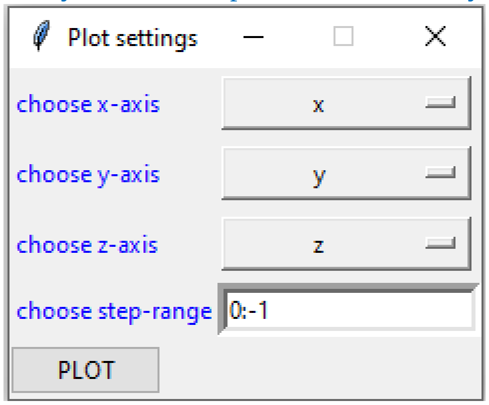

13. Click plot

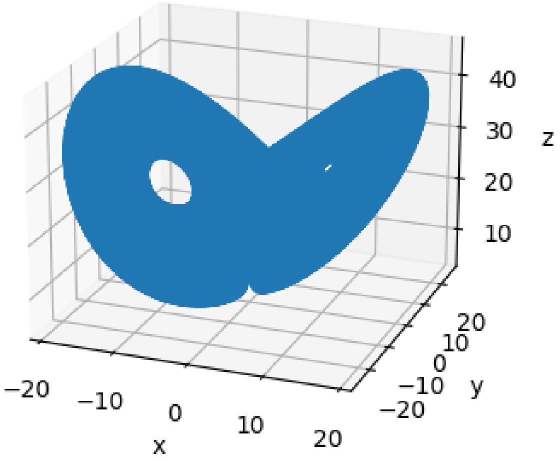

14. Close plot. Change the z-axis to None, the y-axis to z and click plot

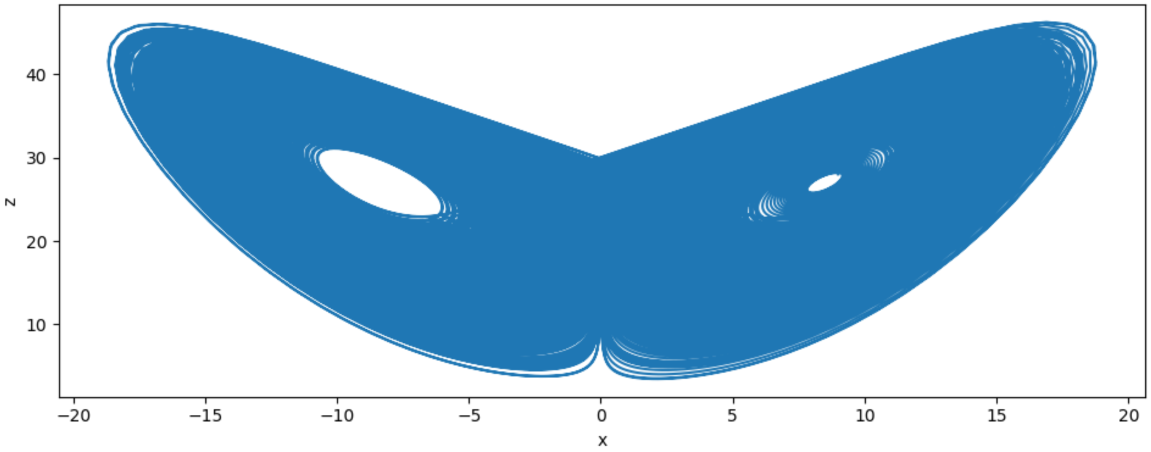

8.4 Simple gene expression model (symbolic LNA and species analytical expression)

```
#REACTIONS
0 None   => mf        , 0.2
     mf  => 0 phi     , 0.2
     mf  => P + mf    , 800
     P   => 0 phi     , 0.09

@CONCENTRATION
None   , 1
mf     , 0
phi    , 0
P      , 0
```

Figure 33. Simple gene expression model.

Modeling steps:

1. File => Open => Topology/File
2. Select the topology "SimpleGeneExpression.dat"
3. Click open
4. Propagation => Analytical soln. => Pure Symbolic :f(t,xo,k)

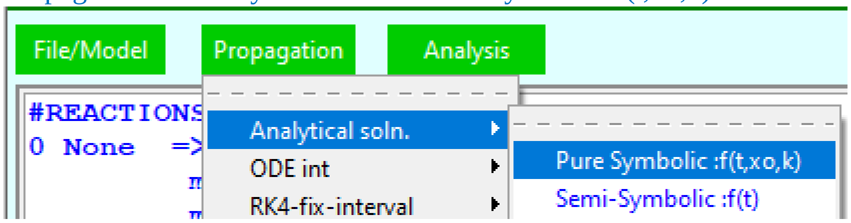

5. Drag down the scroll bar to see result:

```
#REACTIONS
0 None    => mf       , 0.2
         mf  => 0 phi    , 0.2
         mf  => P + mf   , 800
         P   => 0 phi    , 0.09

@CONCENTRATION
None   , 1
mf     , 0
phi    , 0
P      , 0
```

6. The answer is in the text area. Take note of grouping symbols.

```
Complex Analytical expressions


The complex expression is because sympy do not
know how you want to simplify the expression

mf(t)  =  1.0*(kf1*exp(kf2*t) - kf1 + kf2*mfo)*exp(-kf2*t)/kf2

P(t)   =  1.0*(kf1*kf3*(kf2 - kf4)*exp(t*(kf2 + 2*kf4)) -
          kf3*kf4*(1.0*kf1 - kf2*mfo)*(exp(kf2*t) - exp(kf4*t))*exp(kf4*t)
          + (kf2 - kf4)*(Po*kf2*kf4 - kf1*kf3)*exp(t*(kf2 + kf4)))
           *exp(t*(-kf2 - 2*kf4))/(kf2*kf4*(kf2 - kf4))
```

7. We can also see the underlying differential equation that we can do even before we attempt to have an analytical expression in the following:

Propagation => Analytical soln. => For wxmaxima

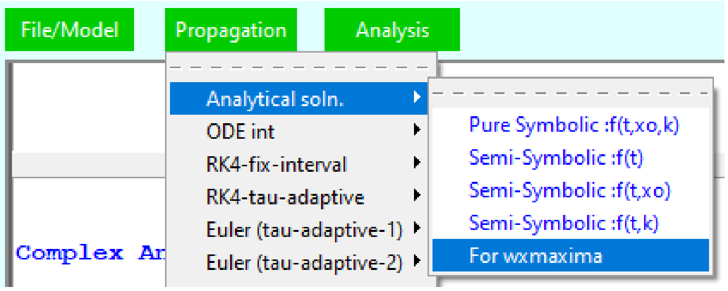

8. The following is the associated differential equation, which we can paste in wxmaxima if we want to solve it using wxmaxima.

```
/*Copy and paste to wxmaxima and run the cell*/

f1:diff(mf(t), t) = 1.0*kf1 - 1.0*kf2*mf(t);
f2:diff(P(t), t) = 1.0*kf3*mf(t) - 1.0*kf4*P(t);

atvalue(mf(t),t=0,mfo);
atvalue(P(t),t=0,Po);

desolve([f1, f2],[mf(t), P(t)]);


/*Copy and paste to wxmaxima and run the cell*/

f1:diff(mf(t), t) = 0.2 - 0.2*mf(t);
f2:diff(P(t), t) = -0.09*P(t) + 800.0*mf(t);

atvalue(mf(t),t=0,0.0);
atvalue(P(t),t=0,0.0);

desolve([f1, f2],[mf(t), P(t)]);
```

9. Now, try another symbolic mode. Follow the following steps:

    1. Propagation => Analytical soln. => Semi-symbolic : f(t)
    2. Drag the scroll bar

```
Simple semi-analytical expression

mf(t)  =  1.0 - 1.0*exp(-0.2*t)

P(t)  =  8888.88888888889 + 7272.72727272727*exp(-0.2*t)
         - 16161.6161616162*exp(-0.09*t)
```

    3. Propagation => Analytical soln. => Semi-symbolic : f(t,xo)

```
Complex Analytical expressions


The complex expression is because sympy do not
know how you want to simplify the expression

mf(t)  =  (1.0*mfo + 1.0*exp(0.2*t) - 1.0)*exp(-0.2*t)

P(t)  =  1.0*Po*exp(-0.09*t) - 7272.72727272727*mfo*exp(-0.2*t)
      + 7272.72727272727*mfo*exp(-0.09*t) + 8888.88888888889
      + 7272.72727272727*exp(-0.2*t) - 16161.6161616162*exp(-0.09*t)
```

10. Now, try LNA (symbolic)

    Follow the following steps:

1. Propagation => Linear Noise Appx. => Symbolic, Macroscopic

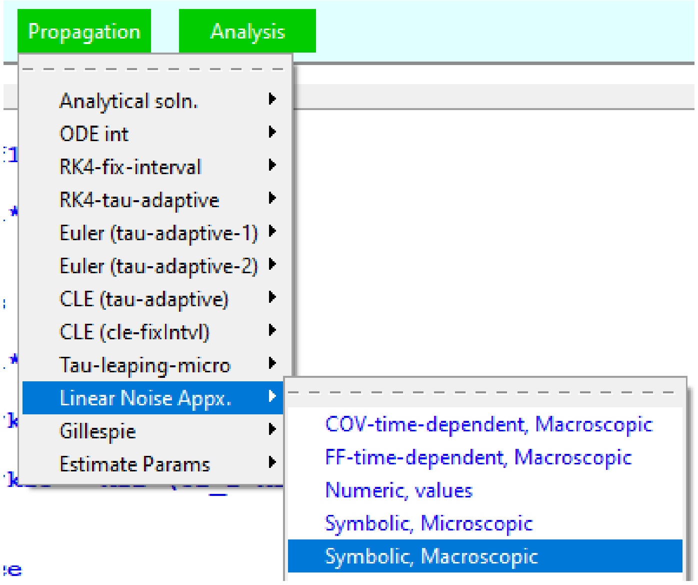

2. Drag scroll bar (now there two scroll bars to drag) to view the result. The derivation step can be seen as well.

```
Using Algebraic manipulation of AC + CA.T + BT = 0

Steady state concentrations

mf  =  kf1/kf2
P  =  kf1*kf3/(kf2*kf4)

Equations to solve

-2.0*C1_1*kf2 + 2.0*kf1
1.0*C1_1*kf3 + C1_2*(-1.0*kf2 - 1.0*kf4)
2.0*(kf1*kf3 + kf2*(C1_2*kf3 - C2_2*kf4))/kf2

Covariance

Cov(mf_mf)  =  kf1/kf2
Cov(mf_P)  =  kf1*kf3/(kf2*(kf2 + kf4))
Cov(P_P)  =  kf1*kf3*(kf2 + kf3 + kf4)/(kf2*kf4*(kf2 + kf4))
```

8.5 Parameter estimation (A => B => C)

To perform parameter estimation, we need both a topology file and trajectory file associated with the topology. The trajectory file is **"tab-delimited,"** which means columns are separated by **"tabs"**. The trajectory has a header row containing column labels such as time and species label. Normally if the user copies a file from Excel and saves it into a text file, it will already be tab-delimited. Several examples of this are provided in the "ParameterEstimation_examples" folder.

8.5.1 All rate constants unknown and all species trajectories are given

The topology shown in **Figure 34** contains a negative rate constant. This is needed in parameter estimation to let BioSANS know which parameter to estimate. For non-mass action topology, we can use concentration substitution as well as propensity modification with a few tweaks.

```
Function_Definitions:
Ao = 100
Bo = 0
Co = 0
kf1 = 100
kf2 = 200

#REACTIONS
A => B, -kf1
B => C, -kf2

@CONCENTRATION
A, Ao
B, Bo
C, Co
```

Figure 34. (A=>B=>C) system for parameter estimation. The real value is 0.5 and 0.3 for kf1, kf2.

Modeling steps:

1. File/Model => Open => Topology/File
2. Choose "AtoBtoC.txt"
3. File/Model => Estimate Params
4. Choose Nelder-Mead (NM), Macroscopic
5. Choose experimental data for "AtoBtoC.txt", which is named "AtoBtoC_data.txt"
6. The following result will appear:

```
Final result =
  final_simplex:
           fun: 3.3751499392044003e-18
       message: 'Optimization terminated successfully.'
          nfev: 134
           nit: 69
        status: 0
       success: True
             x: array([0.5, 0.3])

kf1 = 0.4999999996395983
kf2 = 0.30000000000836313
```

8.5.2 All rate constants unknown and some species trajectories are missing

For experimental data with missing species, we can still perform parameter estimation. In the topology file, we also need to put a negative sign on the initial concentration of missing species. BioSANS will estimate their value during propagation to facilitate rate constant estimation.

```
Function_Definitions:
Ao = 100
Co = 0

#REACTIONS
A => B, -1
B => C, -1

@CONCENTRATION
A, Ao
B, -1
C, Co
```

Figure 35. Parameter estimation with missing species trajectory. Here, B concentrations will also be estimated. The real value of Bo is 0 similar as above.

Modeling steps:

1. File/Model => Open => Topology/File
2. Choose “AtoBtoC_missingB.txt”
3. File/Model => Estimate Params
4. Choose Nelder Mead (NM), Macroscopic
5. Choose experimental data for “AtoBtoC_missingB.txt” which is named “AtoBtoC_data_missingB.txt”
6. The following result will appear:

```
Final result =
  final_simplex:
           fun: 3.501796205184981e-20
       message: 'Optimization terminated successfully.'
          nfev: 347
           nit: 190
        status: 0
       success: True
             x: array([5.000e-01, 3.000e-01, 6.565e-12])

kf1 = 0.5000000000024356
kf2 = 0.29999999999767996
Bo = 6.5654557539791444e-12
```

7. In step 6, the estimate of the initial concentration of missing species is labelled Bo.

We can also perform parameter estimation of various combinations of missing species and rate constants. The more the missing elements, the higher the chances of getting wrong values.

8.5.3 Parameter estimation with propensity modification

The rate constant to estimate in reactions with modified propensity is not easy to detect by BioSANS. We need to create artificial reactions for the rate constant as species so as to not change the concentration. Lines 3 and 4 in the reaction tag shown in **Figure 36** is an example of how to create an artificial reaction for a rate constant. We need to put them also

in the concentration tag with a negative initial concentration for BioSANS to know they have to be estimated.

```
Function_Definitions:
Bo = 0
Co = 0

#REACTIONS
A => B, 1 ::::: lambda k1, A : k1*A
B => C, 1 ::::: lambda k2, B : k2*B
0 NONE => k1, 0
0 NONE => k2, 0

@CONCENTRATION
A,    -1
B,    Bo
C,    Co
k1,   -1
k2,   -1
NONE, 0
```

Figure 36. Parameter estimation with modified propensity. Ao is also unknown.

Modeling steps:

1. Similar as above using "AtoBtoC_missingA.txt" and "AtoBtoC_data_missingA.txt"
2. The following result will appear:

```
Final result =
  final_simplex: (array([[100. ,    0.5,    0.3],
           fun: 1.478441568988223e-20
       message: 'Optimization terminated successfully.'
          nfev: 1015
           nit: 556
        status: 0
       success: True
             x: array([100. ,    0.5,    0.3])

Ao = 100.00000000002035
k1o = 0.49999999999922046
k2o = 0.3000000000007572
```

The reactions "0 NONE => k1, 0" and "0 NONE => k2, 0" means k1 and k2 are constant. The initial amount is -1, which will be treated by BioSANS as estimating an initial amount. The NONE in the concentration tag is needed because it is also present in the reaction tag, although its amount is not changing either. We can see that the k1, k2, and Ao are very well estimated even if the A trajectory is missing.

Parameter estimation for ODE with special function (i.e., Hill function) requires concentration substitution. We could not use propensity modification because BioSANS will not be able to know which rate constant to estimate. We need to encode the ODE in concentration substitution.

8.6 Parameter estimation with Hill function

The basic instruction for handling parameter estimation with modified propensity was covered in section 8.5. It is basically the same for simple to complex propensity functions.

```
Function_Definitions:
alpA = 0.5
betA = 0.2

alpR = 0.5
betR = 0.2


#REACTIONS, Volume = 1, tend = 10, steps = 100, FileUnit = molar
0 NONE => A     , 1   ::::: lambda R, K : betA*K/(K+R)
A     => 0 phi  , alpA

0 NONE => R     , 1   ::::: lambda A, K : betR*A/(K+A)
R     => 0 phi  , alpR

0 NONE => K     , 0

@CONCENTRATION
A     , 0
R     , 0
NONE  , 0
phi   , 0
K     , -1
```

Figure 37. Parameter estimation with Hill function.

Modeling steps:

1. Similar as above using “Q2a_mod.txt” and “Q2a_mod_data.txt”
2. The following result will appear:

```
Final result =
  final_simplex:
       message: 'Optimization terminated successfully.'
          nfev: 42
           nit: 21
        status: 0
       success: True
             x: array([0.2])

Ko = 0.19999999999602372
```

8.7 Parameter estimation at steady state (regular functions)

To perform parameter estimation for simple functions that we don’t need to propagate, BioSANS needs a few simple tweaks that changes the problem to a propagated system. The following examples show how it can be done.

8.7.1 Finding roots ($ax^3 + bx^2 + cx + d = 0$)

```
Function_Definitions:
a = 12
b = -50
c = 30
d = -8409

#REACTIONS
0 NONE => Fx, 1 ::::: lambda x : a*x**3 + b*x**2 + c*x + d
0 NONE => x , 0

@CONCENTRATION
x           , -1
Fx          , 0
NONE        , 0
```

Figure 38. Example of topology in finding roots of functions.

In **Figure 38**, we create a reaction "0 NONE => Fx, 1 :::: **lambda** x : a*x**3 + b*x**2 + c*x + d", which contains the objective function in the modified propensity lambda expression. We also create a reaction for the formation of x. Fx and x as well as NONE appear in the concentration tag as well. The x variable has a negative initial concentration, which will make BioSANS think it is for estimation. The value of Fx should be zero at any time point. We can create a data file for this as follows:

```
time    Fx
0    0
1    0
```

Figure 39. Example data file for simple function.

There are only 2 time points, which is sufficient to constrain our system for BioSANS to know that Fx = 0, dFx/dt = 0. This time, x will be estimated to satisfy the data file.

Modeling steps:

1. File => Open => Topology/File => choose "SteadyStateExample.txt"
2. File/Model => Estimate Params => MCEM, Macroscopic => choose "SteadyStateExample_data.txt"
3. The result will be:

```
Final result =
 [10.402299180583924] 1.8189894035458565e-12 1.3234889800848443e-23
 #Final answer                error estimate-1                 error estimate-2
xo = 10.402299180583924
```

8.7.2 Finding roots for systems of equation

This time we will solve the roots in a system of ODE. The analog is very much the same as in the previous example. Here, x, y, Fx, and Gx as well as NONE are very important.

```
#REACTIONS
0 NONE => Fx, 1 ::::: lambda x, y :  0.5*x**2 + 2*x*y +y**2 - 948.5
0 NONE => Gx, 1 ::::: lambda x, y : -10*x**2 + 12*x*y -15*y**2 + 8830
0 NONE => x, 0
0 NONE => y, 0

@CONCENTRATION
x         , -1
y         , -1
Fx        , 0
Gx        , 0
NONE      , 0
```

Figure 40. Parameter estimation for systems of equations.

```
time    Fx  Gx
0   0   0
1   0   0
```

Figure 41. Data file to facilitate parameter estimation for systems of the equation above.

Modeling steps:

1. File => Open => Topology/File => choose "SystemsOfEquation.txt"
2. File/Model => Estimate Params => Nelder Mead (NM), Macroscopic => choose "SystemsOfEquation_data.txt"
3. The result will be:

```
Final result =
  final_simplex: (array([[ 5., 26.],
       [ 5., 26.],
       [ 5., 26.]]), array([2.403e-17, 3.787e-17, 7.594e-17]))
           fun: 2.4031807344379632e-17
       message: 'Optimization terminated successfully.'
          nfev: 199
           nit: 102
        status: 0
       success: True
             x: array([ 5., 26.])

xo = 4.999999999991312
yo = 26.000000000004242
```

This type of parameter estimation is useful for LNA at steady state given the experimental data are at steady state. We can estimate the variance/covariance at steady state, which will allow us to perform parameter fitting using expressions derived via symbolic LNA.

9 Stochastic Modeling Examples

https://github.com/efajiculay/BioSANS_installers/tree/main/TutorialTopoFiles

9.1 A + B <=> C => D + A

```
#REACTIONS, tend = 1000, tsteps = 100
A + B <=> C     ,0.045  ,0.011
C       => D     ,0.01

@CONCENTRATION
A, 100
B, 90
C, 0
D, 0
```

Figure 42. Bi-molecular reaction with equilibrium.

Modeling steps:

1. File => Open => Topology/File
2. Select the topology "A+BtoCrevtoD.txt"

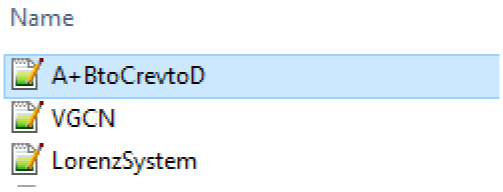

3. Click Open
4. Propagation => Gillespie => Direct method

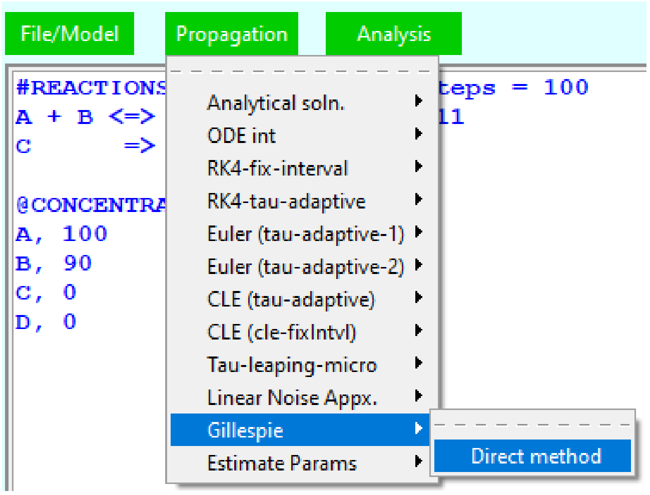

5. Change parameter settings as follows:
   set "logx" to True
   set "mult proc" to True

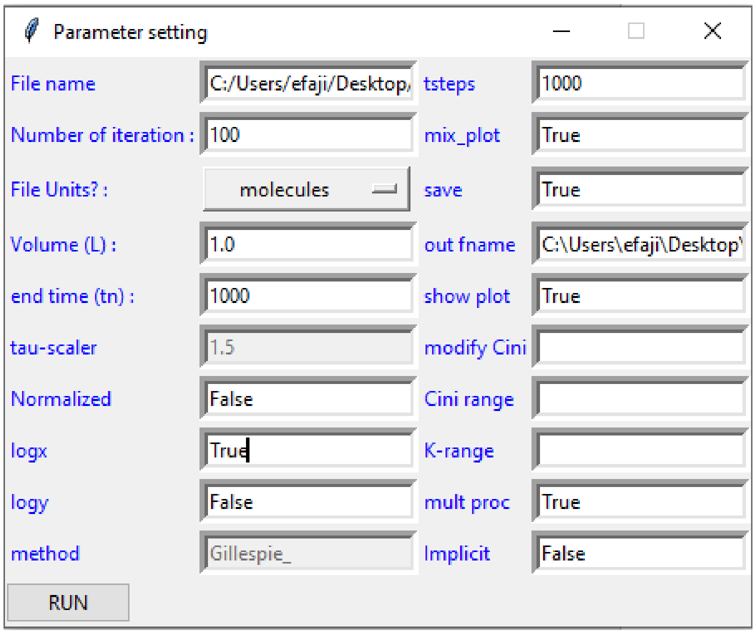

6. Click run

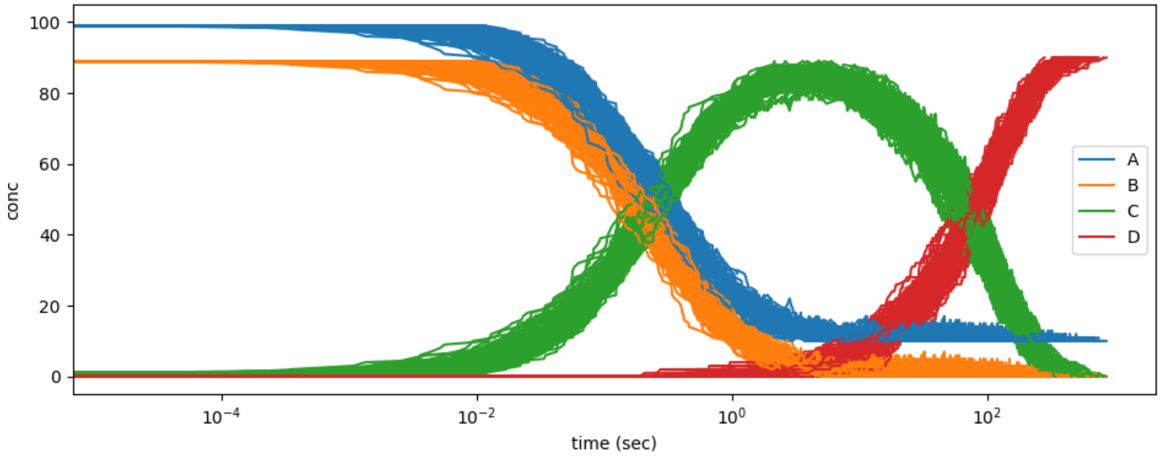

7. Load trajectory file using Open => Trajectory file

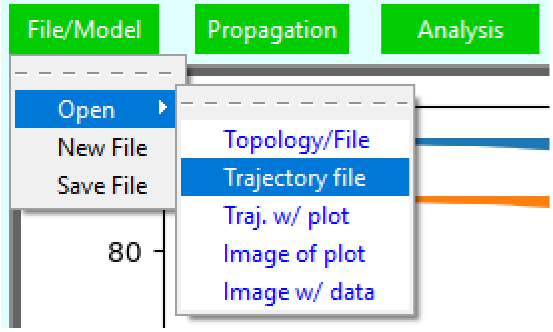

8. Select the .dat file without "_params" in the file name.

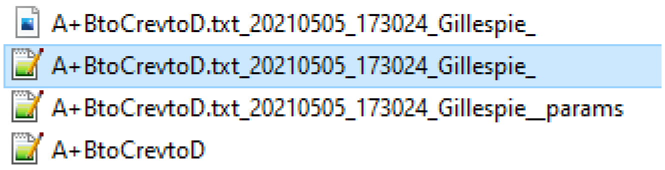

9. Click open
   Wait for a while until the trajectory is fully loaded in memory. A pop-up will confirm that the trajectory loading is successful.
10. Click Analysis => Average of traj.
    This will give an average of the trajectory plot. Axes are not in log here.

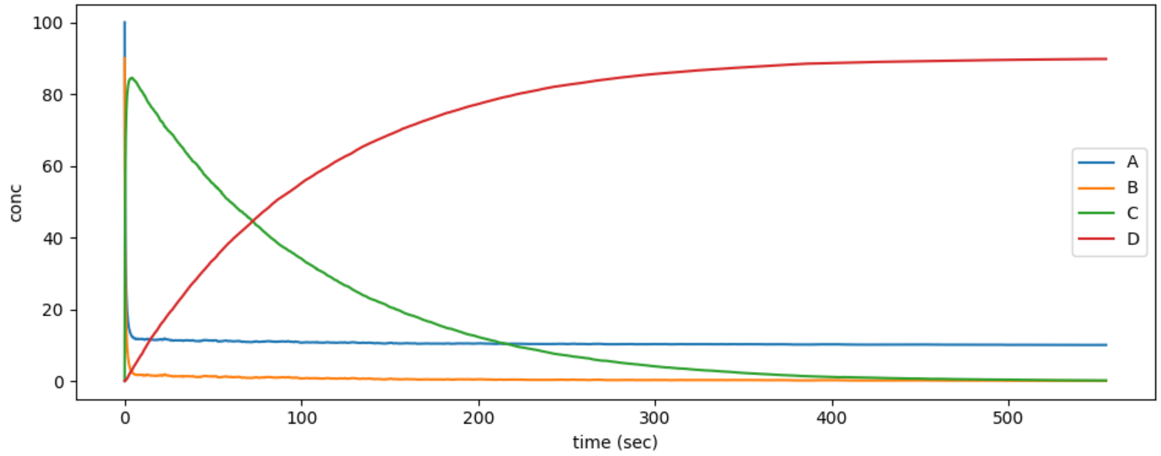

9.2 VGCN SYSTEM

```
#REACTIONS, Volume = 1.0e-19, to = 0, tend = 1, FileUnit = molar, logx = True
    X <=> 2 Y, 512, 512
X + Y <=> Z  ,   8,   8
Y + Z <=> X  ,   1,   1

@CONCENTRATION
X, 0.5
Y, 0.5
Z, 0.5
```

Figure 43. The VGCN system.

Modeling steps:

4. File => Open => Topology/File
5. Select the topology "VGCN.dat"

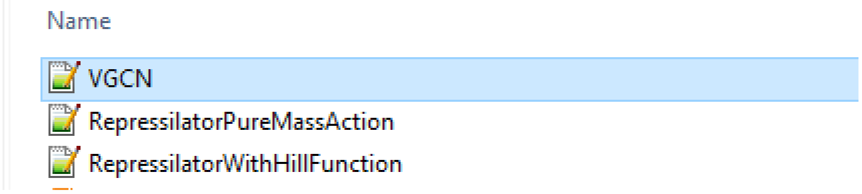

6. Click open

7. Propagation => CLE(tau-adaptive) => Molecules(micro)

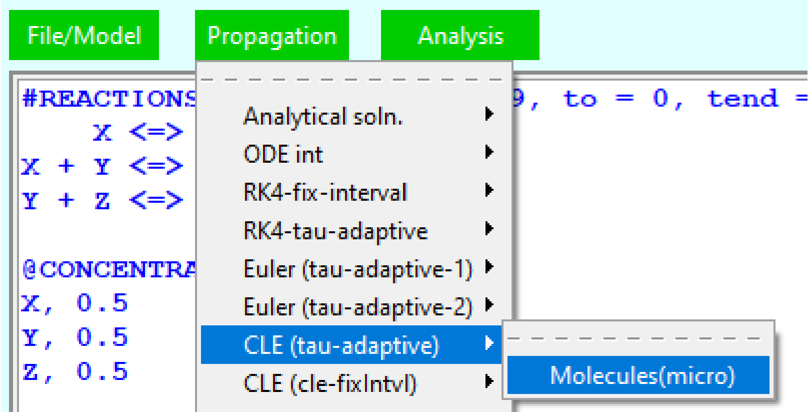

8. Don't change anything in the parameter settings.

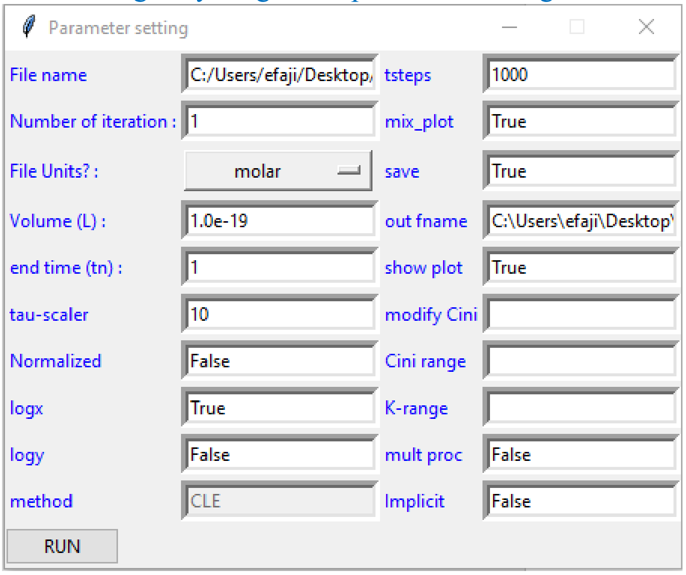

We can see that the file unit is in molars, which is what is in the file. Because this is stochastic simulation, BioSANS will automatically transform the units in the file to molecules.

9. Click run

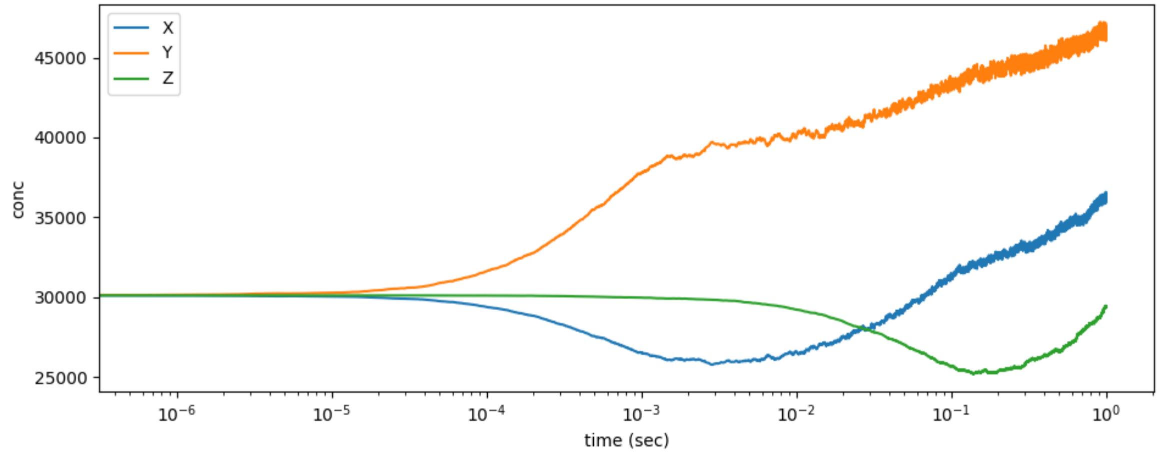

The resulting plot is log-scale time in the x-axis and molecules in the y-axis.

10. Modify the number of iterations to 30 and change “mult proc” to True.

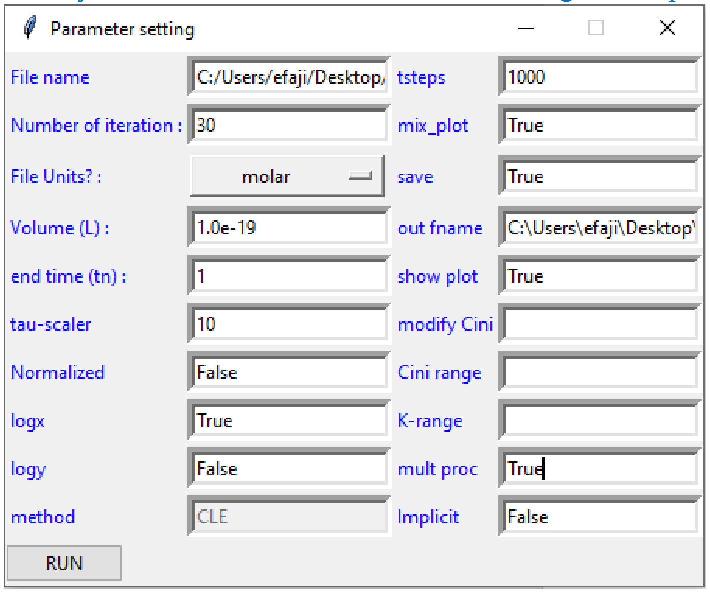

11. Click run

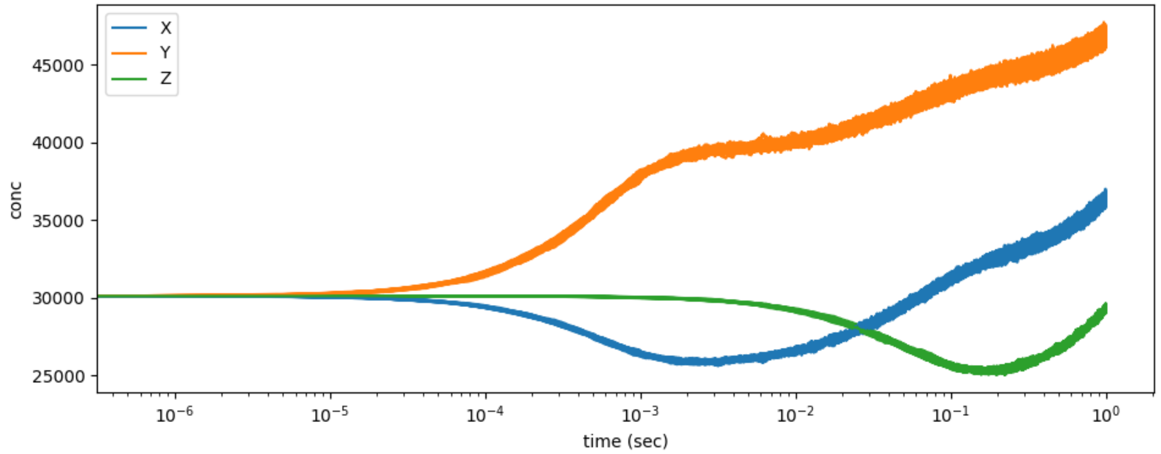

For a faster run, we can set the "show plot" to False. This won't give a plot but if we check the folder, we can see the trajectories.

12. Change the "number of iterations" to 100, and set the "show plot" to False.

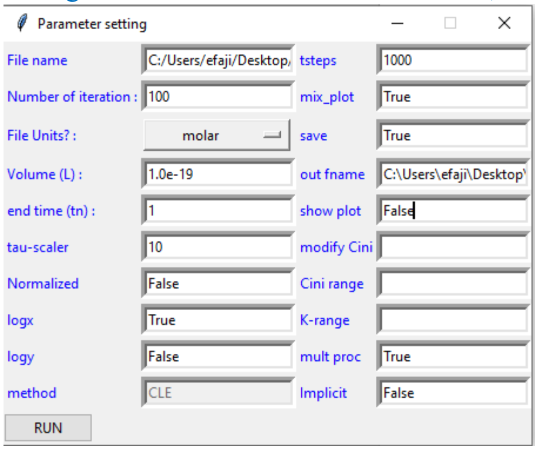

13. Click run
14. Check the files in the working directory if it changes the file size. Wait for a while until it finishes running and change file size.
15. File/Model => Open => Trajectory file => VGCN.dat_YYYYMMDD_HHMMSS_CLE.dat
16. Analysis => Covariance

```
covariance

X and X                                = 40437.039021148084
X and Y                                = -22851.999470890307
X and Z                                = -9308.741380101346
Y and Y                                = 83958.01408417325
Y and Z                                = -5641.603419772824
Z and Z                                = 19430.58271756951

correlation

X and X                                = 1.0
X and Y                                = -0.392196264683901
X and Z                                = -0.33209197312771493
Y and Y                                = 1.0
Y and Z                                = -0.13967817840734545
Z and Z                                = 1.0

Fano Factor

X                                      = 1.1174601970418716
Y                                      = 1.8237930800416395
Z                                      = 0.6662337937092787
```

17. Click Analysis => Probability density

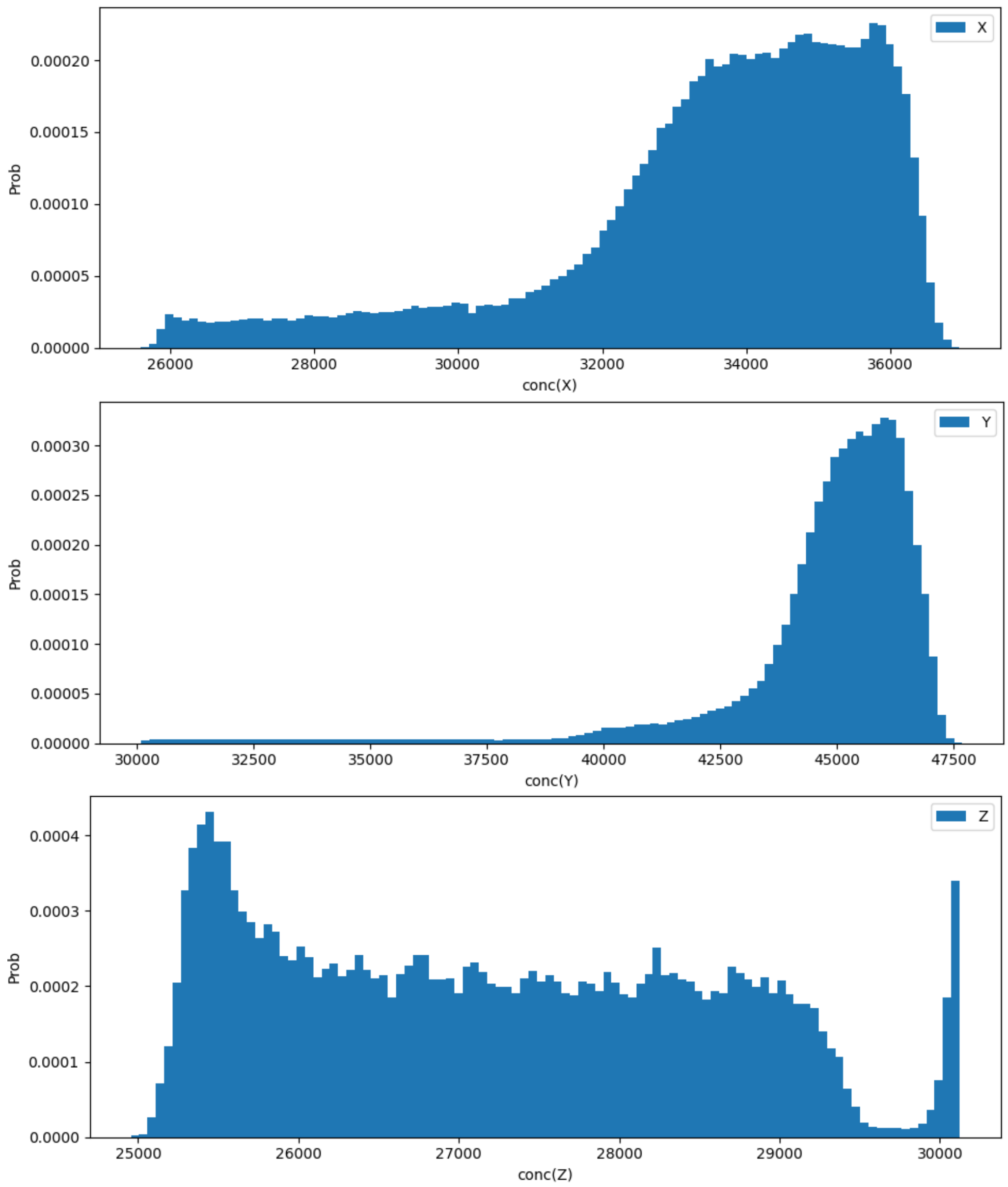

9.3 A <=> B reversible reaction

```
#REACTIONS, tend = 1000, tsteps = 100
A + B <=> C     ,0.045  ,0.011
C       => D    ,0.01

@CONCENTRATION
A, 100
B, 90
C, 0
D, 0
```

Figure 44. Basic reversible reaction.

Modeling steps:

1. File => Open => Topology/File
2. Select the topology "AtoB.dat" and click Open
3. Propagation => CLE (tau-adaptive) => Molecules (micro)
4. Change number of iterations to 100
5. Set "mult proc" to true
6. Click run

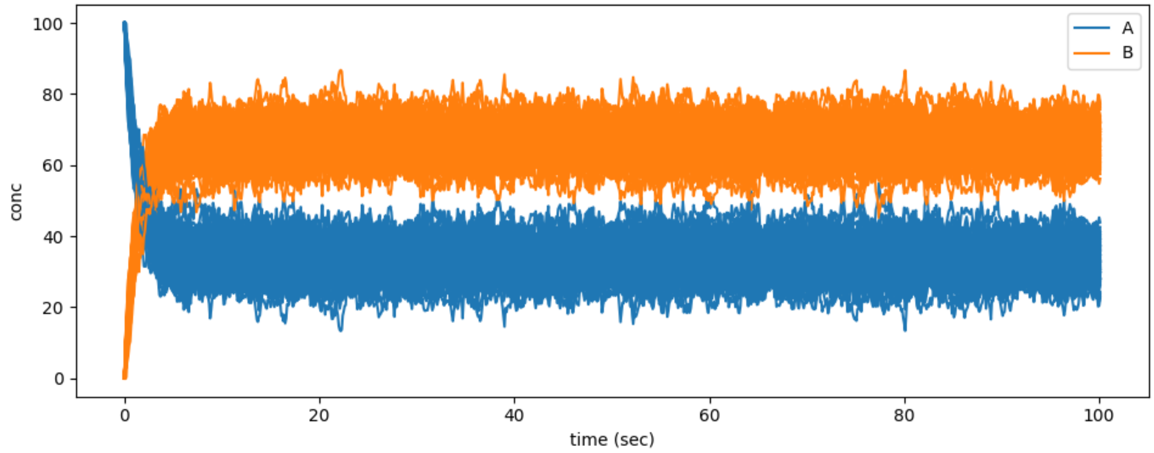

7. Load the trajectory file. It's the ".dat" file without "_params" in the filename. File/Model => Open => Trajectory file => Select traj. File => Click Open
8. Analysis => Freq. Dist w/r to t

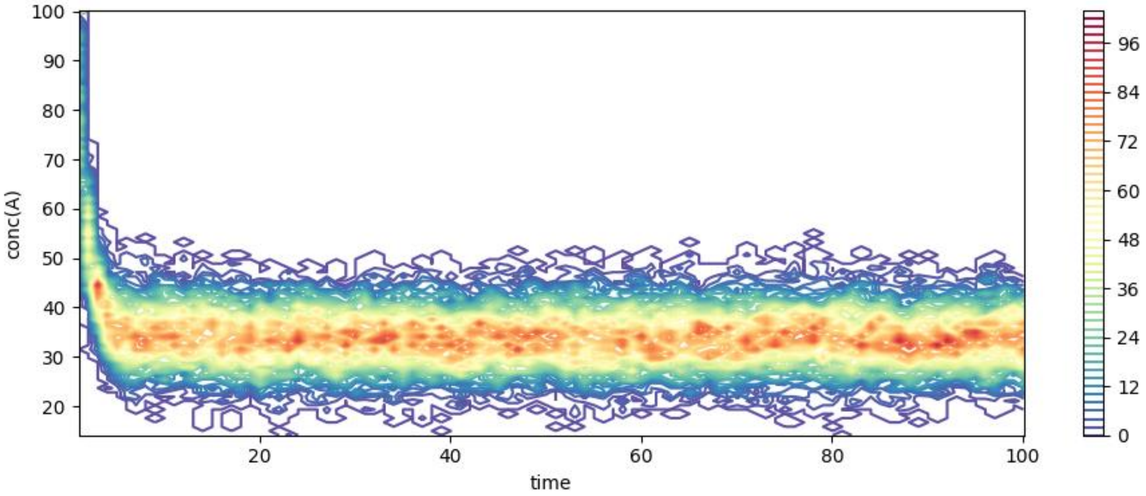

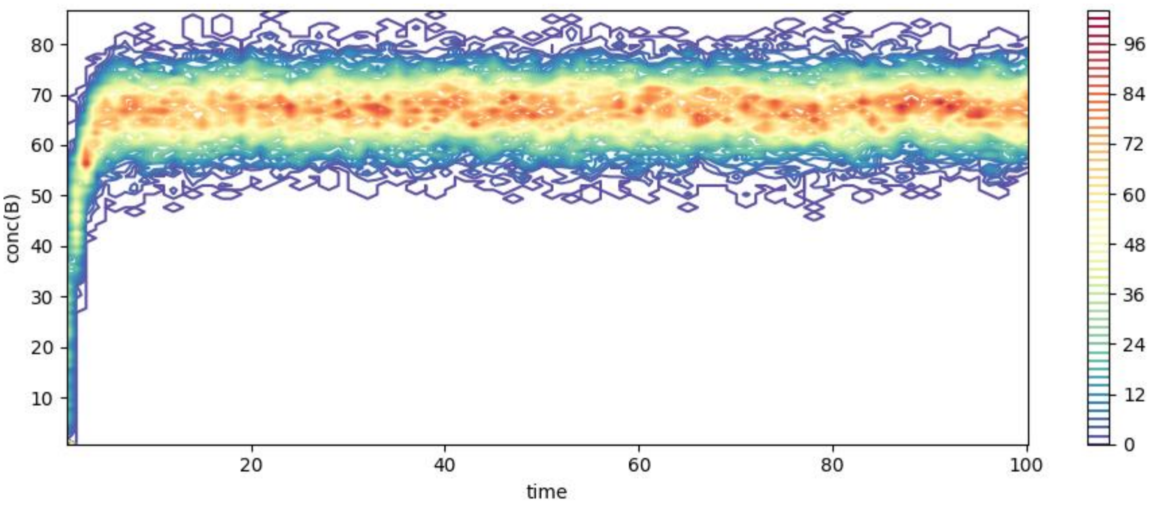

conc(B)
time

10 Algorithm Implementation and Description

10.1 Symbolic computations

BioSANS currently relies on SymPy for symbolic computations. SymPy is among the best computer algebra systems today. Conversely, it does not always give the best simplified result and might arrive at complex expressions that are mathematically correct but domain-wise difficult to interpret. To overcome this, we impose some domain knowledge to drive the computation to a simplified expression whenever possible. It will jump to the use of regular SymPy functions when the suggested solution fails.

10.1.1 Species analytical expression as a function of time

Analytical solutions for species time series are supported for linear systems of ordinary differential equations and a few (separable and simple) non-linear ordinary differential equations. Initially, BioSANS will try a preferred way of derivation based on the topology of the problem. It aims to simplify the solution to a form that is small enough to see some physical insights.

The following equation is a typical form of systems of linear differential equations [6,7]:

$$x' = Ax + b$$

In this expression, $\boldsymbol{x}$ is a vector of chemical species and $\boldsymbol{A}$ is a matrix of rate constants. The preferred solution to this as implemented in the software is as follows:

$$x^* = -A^{-1}b$$

$$x(t) = x^* + e^{At}(x_o - x^*)$$

where $\boldsymbol{x^*}$ is the vector of steady-state concentrations and $\boldsymbol{x_o}$ is a vector of initial concentration. When the matrix exponential part is difficult to converge, we may still be able to solve the problem by expressing $\boldsymbol{A}$ as a sum of 2 matrices and using the Lie product formula. This is still not implemented in the current development. For nonlinear cases, the implemented algorithm will try to use a species-balanced equation to facilitate separation of variables followed by integration.

$$\frac{dx}{f(x^n, x_o)} = g(t)dt$$

If the preferred way of solving did not work, the problem will be transferred to the desolve Python function, which can give answers for most linear ODEs but sometimes not as meaningful as needed for interpretation. The use of steady-state assumption and identification of rate-limiting steps may further help reduce the problem but is currently not implemented.

We provide support to pure analytical expressions, which are a function of time, initial concentration, and rate constant (***f(t,xo,k)***). We also provide support to semi-analytical expressions in which some of the variables are substituted with true values. The semi-analytical expressions are easier to handle and more likely to produce output. The semi-analytical expressions can be reported as a function of time (***f(t)***), time and initial concentration (***f(t,xo)***), and time and rate constant (***f(t,k)***).

10.1.2 LNA covariance matrix and steady-state concentration

The LNA implemented in BioSANS follows the same step-by-step procedure for solving LNA manually manually. We adopt the standard way of LNA formulation based on Van Kampen and Elf [8,9]. For a set of chemical species ***x*** with propensity vector ***a*** and stoichiometric matrix ***V***, we can express the Jacobian matrix ***A*** as follows:

$$A = \frac{\partial Va}{\partial x}$$

and the noise-generating diffusion matrix ***B*** as

$$B = V\, diag(a)\, V^T$$

The covariance matrix ***C*** can be obtained by solving the following Lyapunov matrix equation:

$$AC + CA^T + \Omega B = 0$$

In this equation, Ω pertains to the system volume. This Lyapunov equation can be transformed into a basic linear algebra problem in which ***C*** serves as the coefficient (of a system of linear equations) that is solvable by Gaussian elimination and or by using the Python solve function. All concentration terms in the final expression are then substituted by the steady-state concentration expression of each chemical species.

Pure symbolic and semi-symbolic solutions are also available for LNA analytical expressions. We also allow for reporting the actual values instead of analytical expressions.

10.1.3 Network localization

For the investigation of steady-state perturbation effects, we implement the “Law of Localization in Chemical Reaction Network” introduced by Okada et al. [10]. Their theory gives the relation between sensitivity and structural topology of a network. The sensitivity ***S*** derived from the law of localization can be summarized as follows:

$$S = A^{-1}$$

$$A = \left(\frac{\partial w}{\partial x} | -c_1 \ \dots c_N\right)$$

where $\boldsymbol{w}$ is the propensity vector, $\boldsymbol{x}$ is a vector of chemical species, and $\boldsymbol{c}$ is the basis vector of the nullspace of the stoichiometric matrix. $\boldsymbol{S}$ is a matrix in which the columns can be labelled with their corresponding rate constant and the rows with the corresponding chemical species. The derivation follows a knockdown perturbation, and we can infer from their result that most of the time when the rate constant decreases, the sign of the sensitivity tells what happens to the species concentration. When the rate constant increases, the reverse of the sign of the sensitivity tells what happen to the species concentration.

10.2 Numerical computations

For numerical computations, we took advantage of NumPy and multiprocessing libraries to render simulations that can run in parallel. Both stochastic and deterministic calculations are supported. The following summarizes the numerical algorithm in the software.

10.2.1 Species concentration as a function of time

For systems in which the analytical expression is not easy to derive, species concentration as a function of time can be calculated by numerical integration. We use the "odeint" from Python, which is based on the LSODA algorithm. Euler (2 different tau-adaptive versions) and Runge-Kutta (tau-adaptive and fixed interval) algorithms are coded and are also available for use. We allow for reporting output in explicit (exact simulation times) and implicit (interpolated fixed interval times) modes.

Euler method

We implement the following "tau-adaptive Euler," which is somehow analogous to the tau-leaping algorithm for stochastic simulation. This has a simple form but works fast for most ODE systems except for very stiff problems.

$$x_i(t+\tau) = x_i(t) + \sum_{j=1}^{M} V_{j,i} a_j(x_t)\, \tau * f$$

$$\tau = \min_i \left(\frac{1}{\sum_{j=1}^{M} V_{j,i} a_j(x_t)} \right)$$

In the above expression, $\boldsymbol{V}$ is the stoichiometric matrix, $\boldsymbol{M}$ is the number of reaction channels, $\boldsymbol{a}$ is the propensity vector, and $\boldsymbol{f}$ is a constant that modifies the step size $\boldsymbol{\tau}$, $\sum_{j=1}^{M} V_{j,i} a_j(x_t)$ is the rate of change of x_i, and $min(1/\sum_{j=1}^{M} V_{j,i} a_j(x_t)$ is the time needed for species with the largest rate to change by 1 unit of concentration. Whatever $\boldsymbol{f}$ is, we are allowing the associated species to change that much unit of concentration and other species change by less than $\boldsymbol{f}$ units. Along the time axis, the magnitude of the rate is changing and the species with the largest rate also changes. The overall effect of this is to sample more

points at regions where the rate is too fast (stiff regions) and sample less points where the rate is slow. This provides smoothly sampled points with good resolution on crucial regions of the ODE. For very stiff problems, $\boldsymbol{f}$ needs to be large or the simulation will take too long. Currently, we did not make $\boldsymbol{f}$ adaptive, but we think it would be better if it is also adaptive based on stiffness.

The second tau-adaptive Euler we implement makes use of a second-order Runge-Kutta method to estimate the error in the Euler method. At each iteration, the step size is adjusted such that the error is maintained within some threshold limit, which we set not greater than 10^{-4}.

Runge-Kutta method

Both fixed-interval and tau-adaptive versions are implemented. The fourth-order Runge-Kutta method is used. The Tau-adaptive version is implemented by estimating the error based on higher-order Runge-Kutta methods and adjusting the step size such that the error in every step is maintained. We implement the algorithm proposed by Press et. al. in their paper allowing to minimize the number of function calls while estimating errors [11].

10.2.2 LNA covariance matrix

The numerical way of solving the LNA is exactly the same as the symbolic way except that the Jacobian matrix $\boldsymbol{A}$, the steady-state concentrations, and the final covariance matrix $\boldsymbol{C}$ are numerically evaluated. The numerical solution is important for large systems in which the symbolic expression no longer makes sense and for systems in which symbolic computations are difficult. To compute the steady-state concentrations, the values at some time $\boldsymbol{t}$ close to the steady state is first evaluated by using ODE integrators. The fsolve function is then used to find the actual steady-state concentration starting from the values calculated at time $\boldsymbol{t}$. This aims to avoid numerical values going to regions that are also a null space of the stoichiometric matrix but not feasible to achieve in the current state of the simulation (based on topology and initial concentration).

10.2.3 Estimation of rate constant given some experimental data

We incorporate various optimization algorithms from SciPy and added our version of the Monte Carlo expectation maximization algorithm for parameter estimation. The following summarizes our implementation and the parameter we chose as a default in the SciPy function calls.

Monte Carlo expectation maximization (MCEM)

This function allows for estimating the rate constant by using the concept of metropolis [12] and expectation maximization algorithm [13,14]. Our implementation draws a sample from the posterior distribution using the metropolis algorithm and the expectation step is a simple averaging of the parameters after several sampling steps. For chemical systems, we

assume that the error in the estimated values of the data given the current values of the sampled parameters follows a normal distribution. We define the error as follows:

$$E_i = T_i - X_i$$

where $\boldsymbol{T_i}$ is the true value of the data and $\boldsymbol{X_i}$ is the estimated value given the current parameter $\boldsymbol{K}$. We assume that the mean of the error is zero and we can express the probability of $\boldsymbol{X}$ given $\boldsymbol{K}$ in the following form:

$$P\left(\frac{X}{K}\right) = \prod_{i=1}^{m} \frac{1}{\sqrt{2\pi\sigma^2}} e^{-\frac{E_i{}^2}{2\sigma^2}}$$

The constant $\boldsymbol{m}$ pertains to the number of observations, which can be time points in a chemical kinetics trajectory or number of some measurable observation, here $\boldsymbol{T_i}$. The corresponding log likelihood can be simplified as follows (constants are omitted):

$$ln\left[P\left(\frac{X}{K}\right)\right] = -\sum_{i=1}^{m}(T_i - X_i)^2$$

Sampling involves drawing rate constants (also initial concentration if it is unknown) from a distribution (we use log-normal or normal distribution) and checking the resulting value of the log-likelihood. The ratio of consecutive draws is compared and the current set of rate constants is accepted or rejected based on a uniform random variable. After several draws, the expected values of the rate constant are estimated, as is their standard deviation. They are then used as centers in the next set of sampling and this is repeated until we reach the posterior maximum (typically when the SSE or AAD threshold is met).

Nelder-Mead algorithm

The Nelder-Mead algorithm [15] is a gradient free optimizer available in SciPy that is robust for moderate- to medium-size problems. This requires the function to optimize, initial conditions, tolerance, maximum number of iterations, and other optional inputs. In this development, we use tol = 1.0e-10, maxiter = 100000, and the adaptive = True option. For most cases, it is enough, but probably an advanced user will modify these for extremely large systems (more than 50 species).

Differential evolution

We implement the differential evolution algorithm [16] by importing from SciPy. It is gradient-free and evolutionary in nature, so it is slow but very good if we can provide a good range to search the parameters. In our implementation, we combined differential evolution with the Nelder-Mead algorithm because we normally do not know how to select the proper range of rate constants. The first step is to use the Nelder-Mead algorithm to calculate values close to the optimum solution and expand those values into a range. We set minimum values to an order of magnitude lower than Nelder-Mead result and maximum

values to an order of magnitude higher. The range is then used in the differential evolution algorithm.

Powell

This algorithm [17] is also from SciPy and also does not calculate derivatives. Currently, we set tol=1e-10, maxiter = 100000, ftol = 1e-10, and xtol = 1e-10.

L-BFGS-B

This algorithm [18] is gradient-based and is also from SciPy. This is a fast algorithm but more likely to get stuck on local minima and not suitable for problems for which the gradient is likely to become rough and flat. We use the same settings as in the Powell algorithm as a default.

Parameter slider

This is a simple GUI element that allows a user to investigate the effect of parameter perturbation on the overall time series plot. It also allows for tuning the parameters manually by dragging the slider until it produces a plot that matches the graph of experimental data.

10.2.4 Propagation of stochastic trajectories

We code various stochastic simulation algorithms and modified versions of those algorithms. A time-step scaling hyper-parameter is added that can speed up the simulation by a constant factor giving approximate trajectories. This is to facilitate the need for urgent answers to problems, which requires only qualitative trajectory information. There is a trade-off between accuracy of the simulation and speed, but a properly chosen scaling parameter will allow for obtaining trajectories that can be used to explain biological phenomena.

Chemical Langevine equation

The basic chemical Langevine equation [19] published 20 years ago was implemented. A modified version we call tau-adaptive CLE was developed, which is similar to the tau-adaptive Euler discussed above. This time we also use the drift term to decide for $\boldsymbol{\tau}$ as follows:

$$x_i(t+\tau) = x_i(t) + \sum_{j=1}^{M} V_{j,i} a_j(x_t) * \tau * f \; + \sum_{j=1}^{M} V_{j,i} \sqrt{a_j(x_t) * \tau * f} * N$$

$$\tau = min\left(\frac{1}{\sum_{j=1}^{M} V_{j,i} a_j(x_t)}, \frac{1}{\sum_{j=1}^{M} V_{j,i} \sqrt{a_j(x_t)} * N}\right)$$

where ***N*** is the normal distribution with mean 0 and variance of 1. Similar to tau-adaptive Euler, this allows for more samples to be taken for regions where $\sum_{j=1}^{M} V_{j.i} a_j(x_t)$ is big or the stiff regions of the trajectory provide higher resolution to crucial regions of ODE, which can have substantial information.

Tau-leaping algorithm

We implement the new tau-selection procedure [20] for the tau-leaping algorithm but also added a scaling factor ***f*** for ***dt*** to allow for speed-up of computation as needed. The following set of expressions describes how the tau-leaping algorithm is propagated.

$$x_i(t+\tau) = x_i(t) + \sum_{j=1}^{M} V_{j,i}\, P_j\big(a_j(x_t), \tau\big)$$

$$\tau = min\,(\tau', \tau''),\ \epsilon = 0.03{*}f$$

$$\tau' = min\left\{ \frac{max\left\{\frac{\epsilon x_i}{g_i}, 1\right\}}{\left|\sum_{j\epsilon j_{ncr}}^{M} V_{j,i}\, a_j(x_t)\right|}, \frac{max\left\{\frac{\epsilon x_i}{g_i}, 1\right\}^2}{\sum_{j\epsilon j_{ncr}}^{M} {V_{j,i}}^2\, a_j(x_t)} \right\}$$

$$\tau'' = \frac{1}{a_o^c}\, ln\left(\frac{1}{r_1}\right)$$

In the expression above, ***ncr*** superscript pertains to non-critical reactions and ***P*** is the poison probability distribution. Non-critical reactions are the reactions that will not end up consuming the reactants after firing several times (≥10). The ***c*** superscript pertains to critical reactions that are close to consuming all reactants. Tau is selected such that a critical reaction will not fire more than once in one tau-leap. The x_i in the numerator is the number of species ***i*** at that particular moment, v_i is the stoichiometric vector for species ***i***, and g_i is a function of the highest order of reaction (HOR) as follows:

$$g_i = \{1\ if\ HOR(i) = 1$$
$$2\ if\ HOR(i) = 2$$
$$2 + \frac{1}{x_i - 1}\ if\ HOR(i) = 2\ and\ S_i = 2$$

The S_i =2 in the last condition means that there are 2 molecules of the same species that contribute to make HOR = 2. We also implement our variants of the above algorithm in the current development.

Gillespie algorithm

The standard implementation of the Gillespie algorithm known as the stochastic simulation algorithm [21] was adopted. It assumes that the probability of reaction is proportional to

the propensity ***a*** multiplied by ***dt***. The choice of which reaction to propagate at a particular time is based on the normalized cumulative probability of each reaction R_j, which can be expressed as follows:

$$f(R_j) = \frac{\sum_{i=1}^{j} a_i}{\sum_{i=1}^{m} a_i}$$

where ***m*** pertains to the number of chemical reactions and ***j*** to the index of reaction. A uniform random variable $\boldsymbol{r_1}$ is drawn and if it falls into the region of $\boldsymbol{f(R_j)}$, then the concentration of each species $\boldsymbol{x_i}$, which are reactants of $\boldsymbol{R_j}$, will be adjusted by their corresponding stoichiometric vector $\boldsymbol{v_{i,j}}$.

$$x_i = x_i + v_{i,j}$$

Time is incremented based on another uniform variable $\boldsymbol{r_2}$ as follows:

$$t_{n+1} = t_n + \frac{1}{\sum_{i=1}^{m} a_i} ln\left(\frac{1}{r_2}\right)$$

10.2.5 Miscellaneous functions

Post-simulation processing functions are also included to transform and manipulate trajectory data for calculating statistical parameters. It also allows for plotting distribution, correlation, time density distribution, etc. that may be useful in interpreting the data.

11 BioSANS testing results

11.1 Symbolic test cases

The following figures are the test cases used for species analytical expression and parameter estimations. The test cases include varying complexity of reaction topology that can be classified as follows: linear chains, reversible reactions, reactions with cyclic structures, overlapping reversible reactions, overlapping cyclic structures, mixture of reversible and cyclic structure, and overlapping reversible and cyclic structures. Each of those classes has various levels of difficulty in arriving at the analytical expression and determining parameters. Most of the cases are linear differential equations with only a few non-linear differential equations.

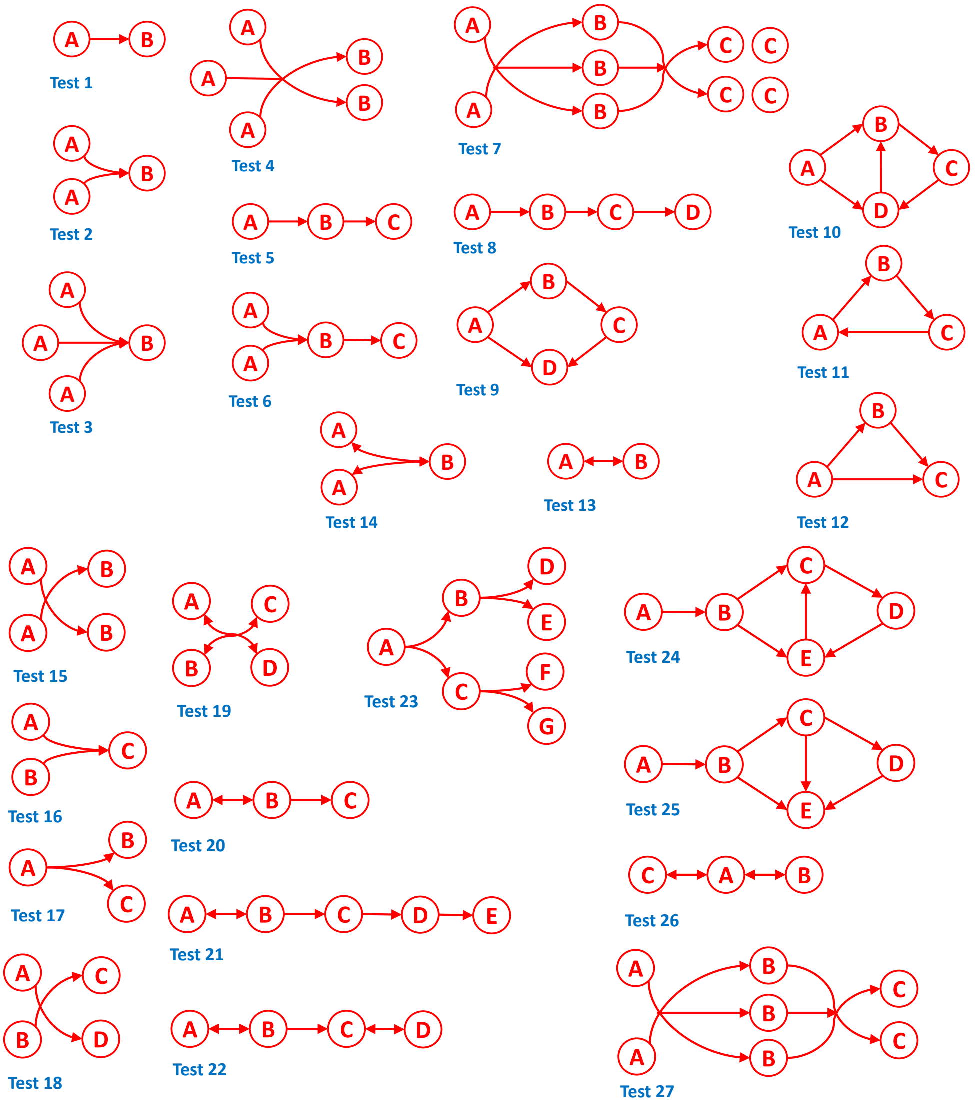

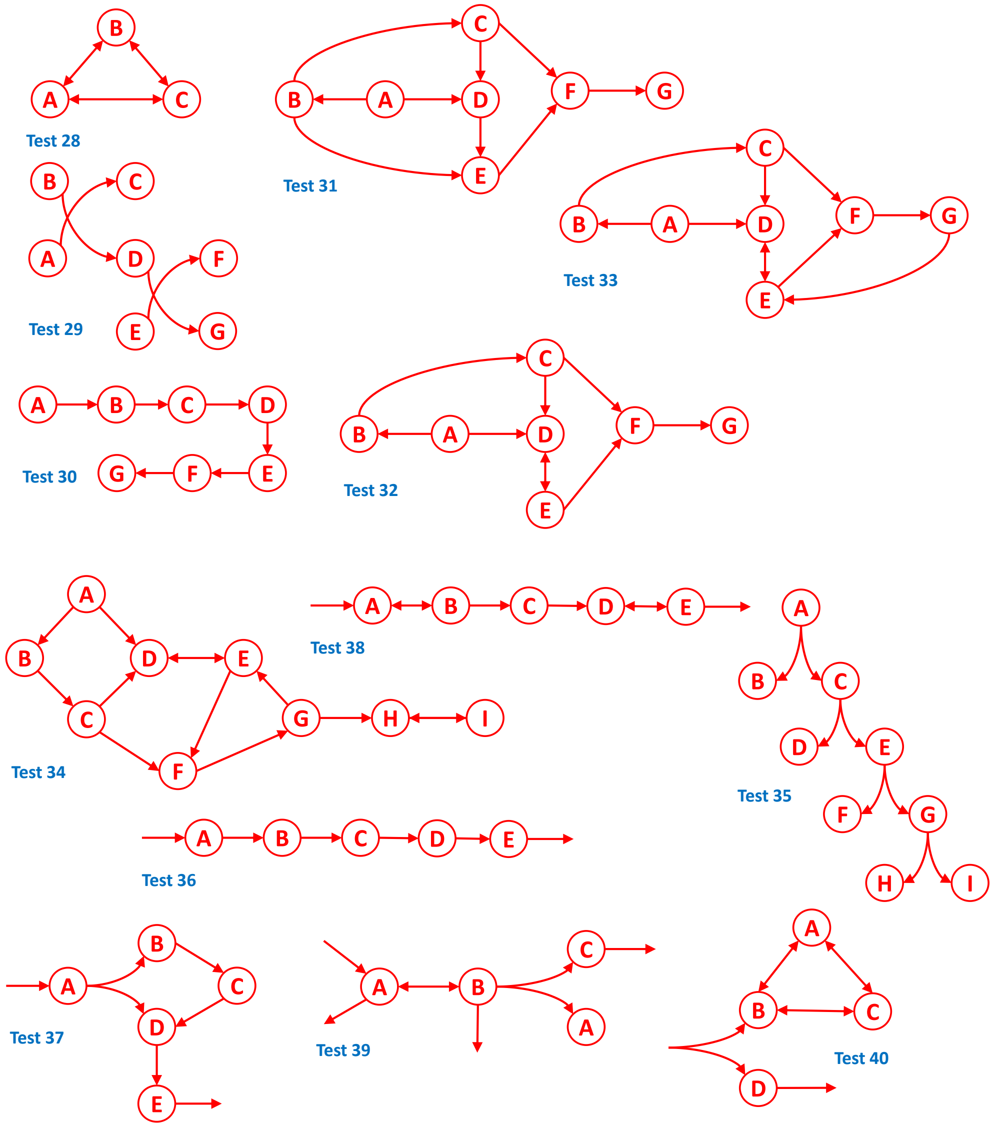

Figure 45. List of test cases topology for symbolic analytical expression and parameter estimation. Test case number 5 with modified difference in order of magnitude was used as an additional test case (41 to 45) for parameter estimation.

The following figures are test cases for LNA and steady-state concentration expressions:

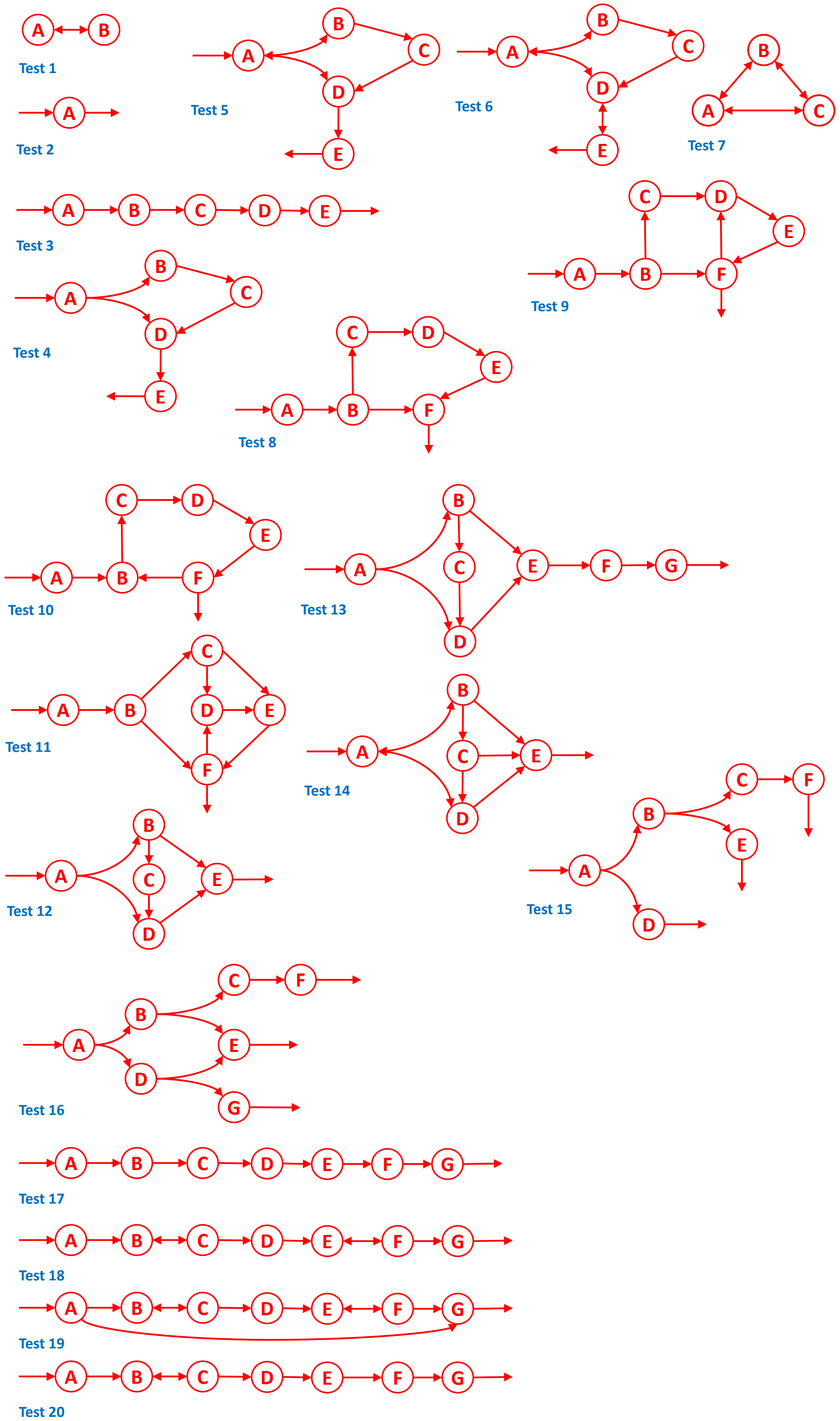

Figure 46. List of test cases topology for symbolic LNA expression and steady-state concentration.

11.2 Performance on semantic test

The following table summarizes the results for BioSANS for the SBML semantic test cases. Currently BioSANS passes most of the test cases with > 70% correct cases.

Table 4. List of test cases in SBML semantic test. Those in green are the test cases for which BioSANS produced correct results.

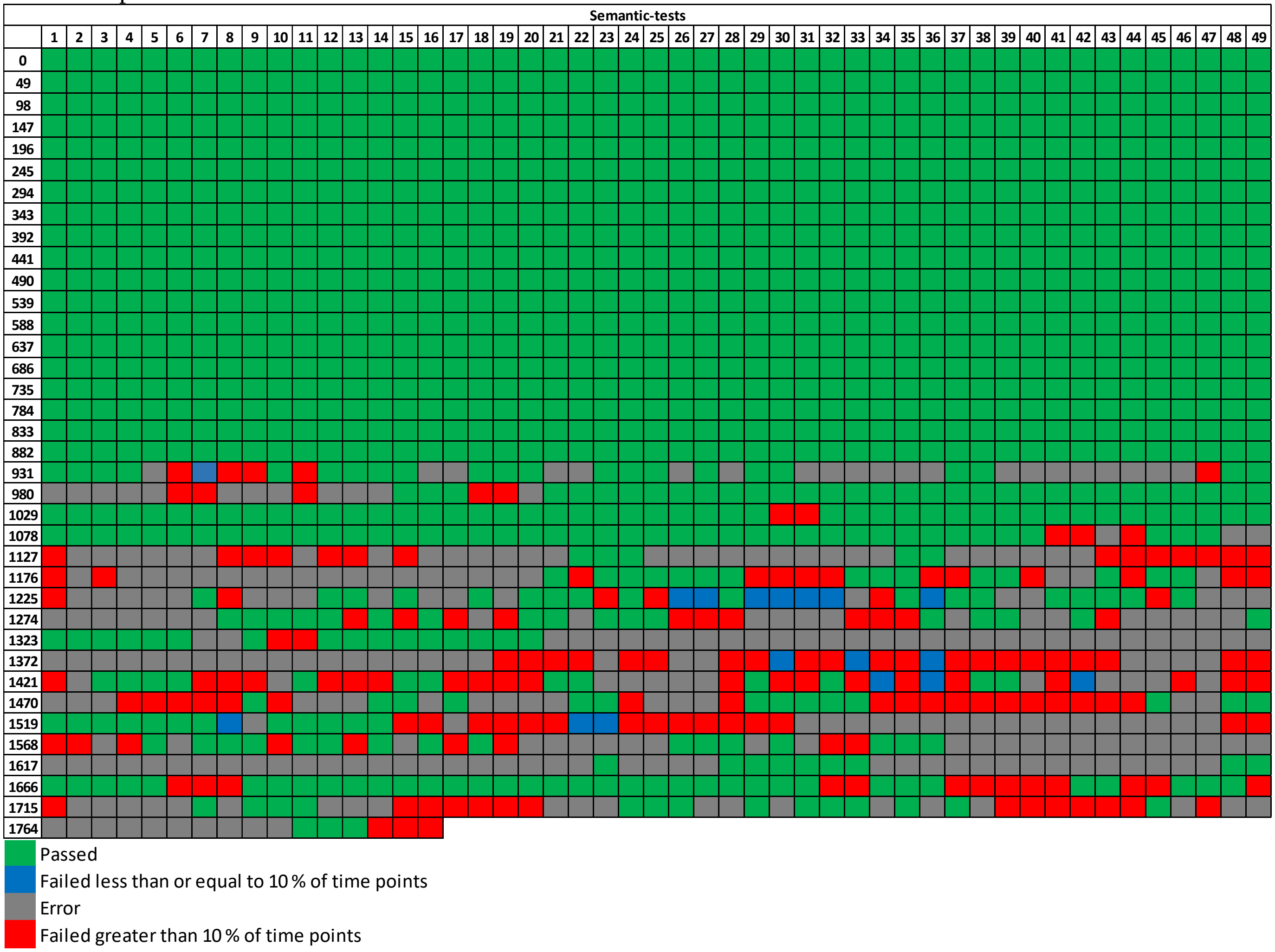

11.3 Performance on stochastic tests

Table 5. Performance of stochastic algorithms under the exact and inexact test.

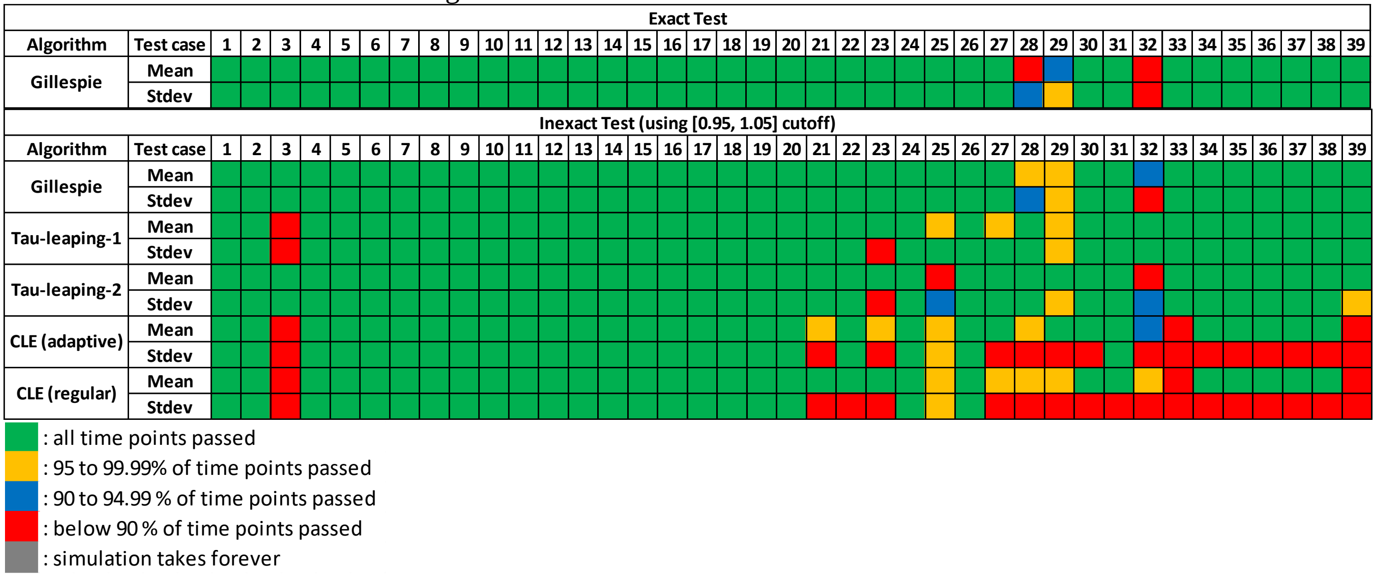

The image above shows that most algorithms fail in test case number 3. This is expected based on SBML documentation. The Gillespie direct method we provide passed the test but sometimes fails in the standard deviation test. In **Figure 47**, we compare the standard deviation plot in SBML with the standard deviation plot in our implementation for this particular case. It is only slightly different when it fails the test, and the actual values is just a matter of a few decimal points different.

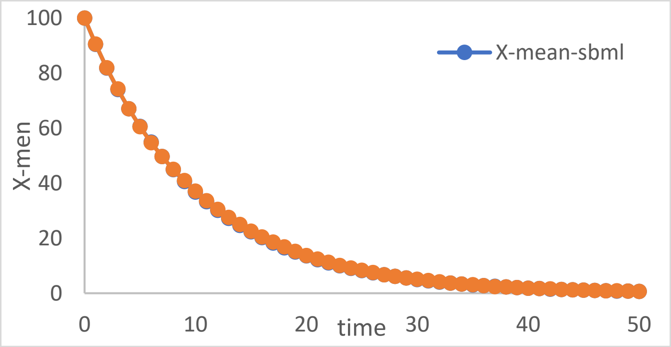

Figure 47. Performance on SBML-DSMTS test case 3.

For test case 32, 50% of tests failed in mean and 11% in standard deviation with the Gillespie direct method because we implemented delay and events in a slightly different way, so our trajectory deviates from SBML-test case 32. They are probably using a special type of delay and events dedicated for exact algorithms such as Gillespie. The delay and event approach we use in the semantic cases is also what we use in the stochastic case. We will adopt new algorithms for delay and events in stochastic cases in future BioSANS updates.

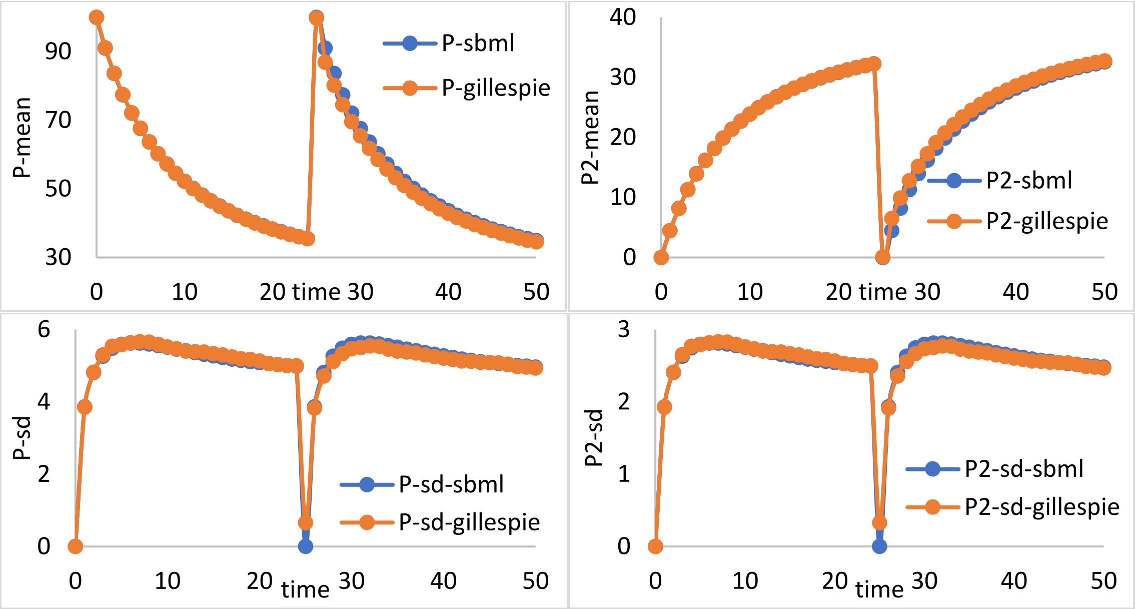

Figure 48. Performance on SBML-DSMTS test case 32.

We have the same explanation for test cases 28 and 29, in which the results for SBML and our Gillespie-direct method slightly differ because of some implementation discrepancy. More or less, our implementation passed the stochastic test case of not more than 3 failures in the mean test and not more than 6 in the standard deviation test.

For the inexact test, the Gillespie algorithm still shows good performance. Our implementation of tau-leaping algorithm passed most of the inexact tests, and as expected, CLE failed many of the tests because most test cases are beyond the assumptions in the CLE algorithm. The figures above show only one of the many possible outcomes after testing. The outcome can change for a particular run.

11.4 Performance in symbolic tests

Table 6. Performance in derivation of analytical expression for chemical species.

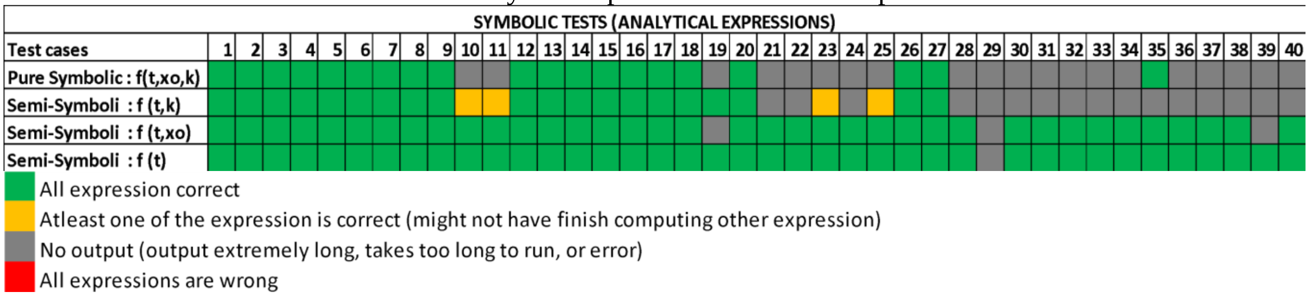

Table 7. Performance in derivation of LNA expression.

SYMBOLIC TESTS (LINEAR NOISE APPROXIMATION)																					
Algorithm	Test cases	1	2	3	4	5	6	7	8	9	10	11	12	13	14	15	16	17	18	19	20
LNA : symbolic -f(xo,ks)	mean at Steady State																				
	variance at Steady state																				
LNA: symbolic-f(xo)	mean at Steady State																				
	variance at Steady state																				
LNA : symbolic-f(ks)	mean at Steady State																				
	variance at Steady state																				
LNA : symbolic-values	mean at Steady State																				
	variance at Steady state																				

All expression correct
Takes forever
Wrong answer

11.5 Performance in parameter estimation

Table 8. Performance of various algorithms in parameter estimation.

Quantitative Parameter Estimation Tests																																													
Test cases	1	2	3	4	5	6	7	8	9	10	11	12	13	14	15	16	17	18	19	20	21	22	23	24	25	26	27	28	29	30	31	32	33	34	35	36	37	38	39	40	41	42	43	44	45
Nelder-Mead (NM)																																													
Monte Carlo EM																																													
Differential Evolution (DE)																																													
NM-DE-NM combined																																													
Powell																																													
L-BFGS-B																																													

trajectory close to true trajectory and parameter estimates are close to true value
Same as above but runtime more than 20 minutes
Same as above but need to manually provide search boundary; provided boundary is narrow which is the reason it can find the answer
trajectory close to true trajectory but some rate constant is different from true value (stuck at local minima)
takes too long, can give result if waited long enough
calculated rate constants are wrong or too far from true value even if sometimes trajectory looks correct

Table 9. Performance of parameter slider.

Qualitative Parameter Estimation Tests																																													
Test cases	1	2	3	4	5	6	7	8	9	10	11	12	13	14	15	16	17	18	19	20	21	22	23	24	25	26	27	28	29	30	31	32	33	34	35	36	37	38	39	40	41	42	43	44	45
Parameter slider																																													

trajectory close to true trajectory and parameter estimates are close to true value
trajectory close to true trajectory but rate constant is different
Not easy to match true value, requires quantitative algorithm

12 Additional Examples

https://github.com/efajiculay/BioSANS_installers/tree/main/TutorialTopoFiles

12.1 BioSANS console interface (examples)

SSL is a console interface in BioSANS that has similar syntax to MySQL commands. It is an alternative to GUI and uses Python as a library.

If the user installs BioSANS using an Anaconda or Python terminal, BioSSL can be lauched by typing BioSSL and pressing enter. For Anaconda users who created a BioSANS environment, they need to activate the environment first before the BioSSL command. If it was named BioSANS (following the installation tutorial in section 1.2.1), just type “conda activate BioSANS”.

BioSSL can also be launched from the BioSANS tab by choosing “File/Model => RunSSL”. The complete list of commands that BioSSL accepts is discussed in section 6.3. This section focuses on example systems. Refer to section 6.2.1 for the list of methods.

12.1.1 A => B => C (stochastic simulation)

The system shown in **Figure 30** can be modelled by typing it in the BioSSL console or by saving it into a file and loading it in BioSSL using the load command.

Modeling steps:

1. Type the following command in console. Don’t forget “;” in the end of command.

```
propagate A => B, 0.2 & B => C, 0.3 where A=100 & B=0.2
& C=0 using CLE with tn=50 & tlen=1000 & miter=30 &
mult_proc=True & fout=example1;
```

2. Press enter
3. When the run is finished, the user will see “>” in the console:

```
>
> propagate A => B, 0.2 & B => C, 0.3 where A=100 & B=0.2 & C=0 using CLE with
tn=50 & tlen=1000 & miter=30 & mult_proc=True & fout=example1;
>
```

4. Check the current working directory by running the following command

   pwd;

5. The current directory will be printed in the screen.

```
> pwd;
C:/Users/Erickson/BioSSL_temporary_folder
```

6. To list the contents of current working directory, issue the following command:

   ls;

7. The contents of current directory will be printed.

```
> ls;
file        : example1_CLE.dat
file        : example1_CLE.jpg
file        : temp.txt
```

8. To open the current working directory, type the following command:
   open_cwd;

9. The folder of the directory will be shown with the files:

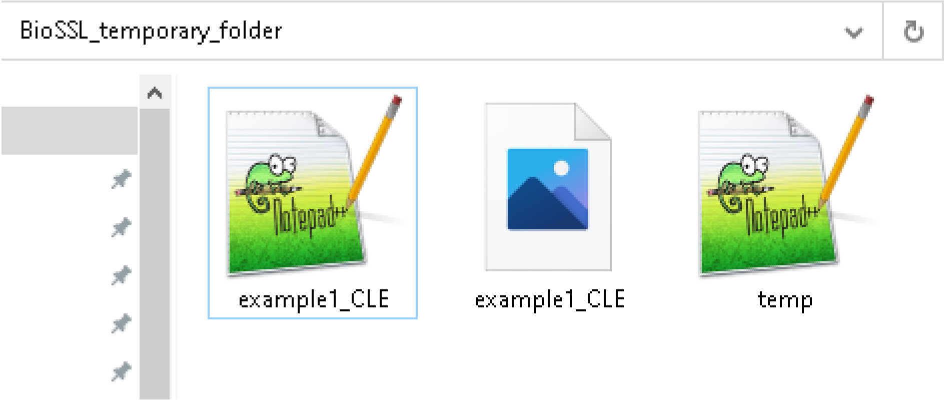

10. Check each file. The content of temp.txt is the equivalent topology file of the SSL command in the propagation.

```
#REACTIONS
A => B, 0.2
B => C, 0.3

@CONCENTRATION
A,100
B,0.2
C,0
```

Example1_CLE.jpg contains a plot of the data:

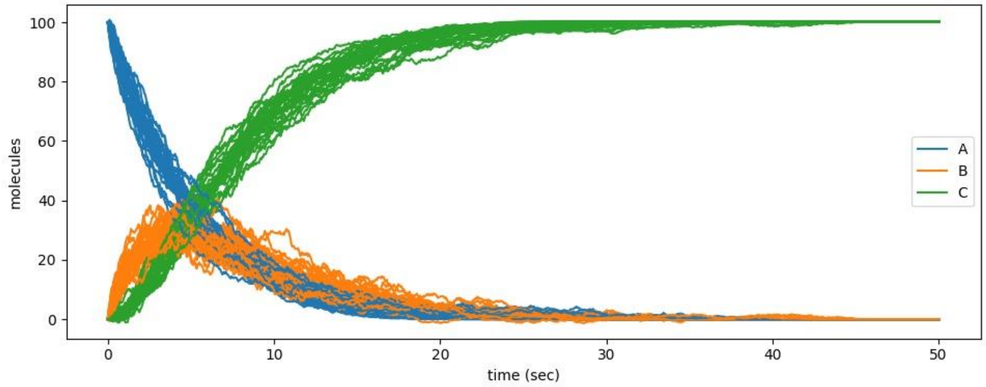

Example1_CLE.dat contains the trajectory:

```
time	A	B	C
0.0 100.0	0.2 0.0
0.04999999999999716 97.23594765403244	2.9391348330104554	0.024917512957101522
0.09999999999999432 95.2984713923171	4.362005746451036	0.5395228612318713
0.14999999999999147 92.52235908866797	7.3226688568727925	0.35497205445924196
0.19999999999998863 90.68325928659557	9.102091618500003	0.41464909490442897
0.2499999999999858 89.87471969231544	9.622383067373754	0.7028972403108071
0.29999999999998295 88.8394159081996	9.960850137664234	1.399733954136167
```

11. We can read the trajectory data into a variable for further analysis and processing.
    read_traj example1_CLE.dat as data;

12. The trajectory is saved in the variable data, which we can print and perform numerical calculations.
    print data;

```
> print data;
([array([[ 0.00000000e+00,  1.00000000e+02,  2.00000000e-01,
           0.00000000e+00],
         [ 5.00000000e-02,  9.72359477e+01,  2.93913483e+00,
           2.49175130e-02],
         [ 1.00000000e-01,  9.52984714e+01,  4.36200575e+00,
           5.39522861e-01],
         ...,
```

    calc_covariance data 100;

```
> calc_covariance data 100;
covariance

A and A                                    = 1.692711865184094e-36
A and B                                    = -1.8055593228630336e-35
A and C                                    = -7.395570986446986e-32
B and B                                    = 1.925929944387236e-34
B and C                                    = 7.888609052210118e-31
C and C                                    = 3.2311742677852644e-27
```

The small values are because A=>B=>C goes to completion and the last 100 points A, B, and C are almost not changing (constant).

13. We could also plot the data but we need to read the data using pdread_traj.
    pdread_traj example1_CLE.dat as data2;

14. Printing from data2 looks better because it is panda-based.
    print data2;

```
> print data2;
     time           A         B         C
0    0.00  100.000000  0.200000  0.000000
1    0.05   97.235948  2.939135  0.024918
2    0.10   95.298471  4.362006  0.539523
3    0.15   92.522359  7.322669  0.354972
4    0.20   90.683259  9.102092  0.414649
...   ...         ...       ...       ...
```

15. Plot the result:

plot data2 time A B C;

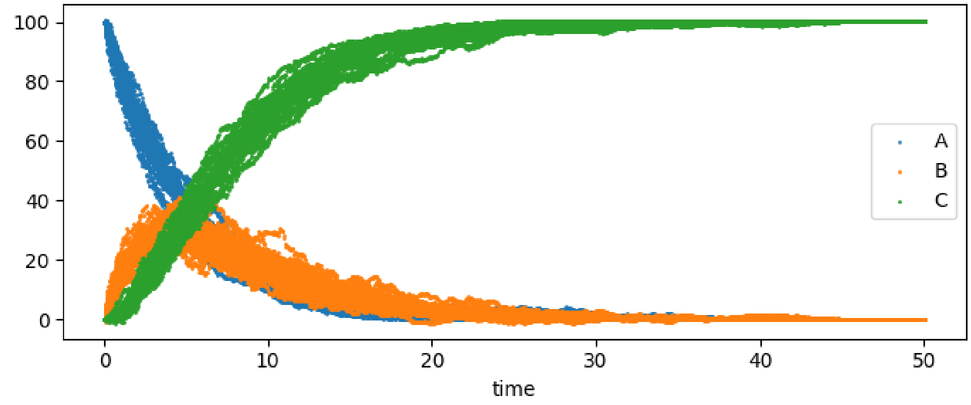

Plotting the phase portrait is relatively easy:

plot data2 A B;

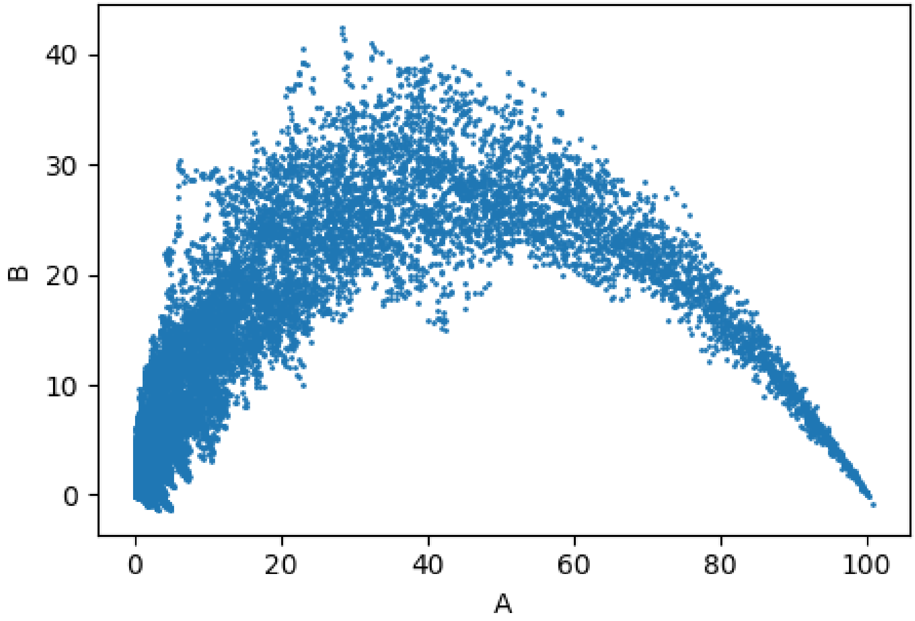

16. To generate a probability density plot, enter the following commands:

prob_density_wtime data;

17. Open in current folder

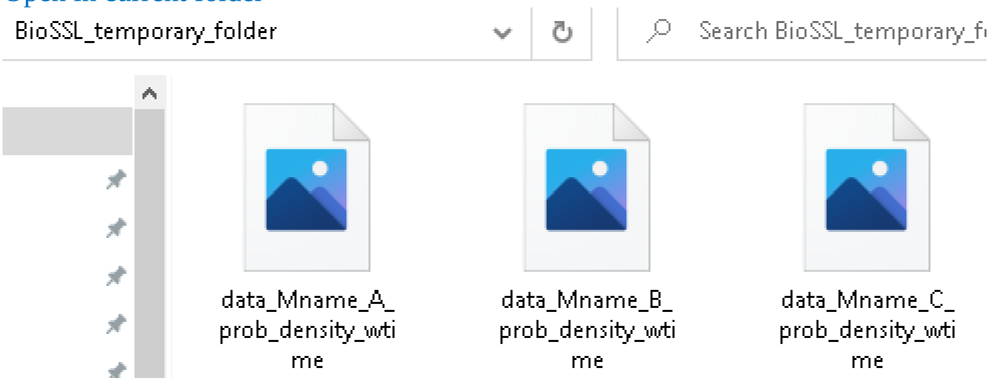

Those plots will look better if we use at least 1000 trajectories in our propagation step.

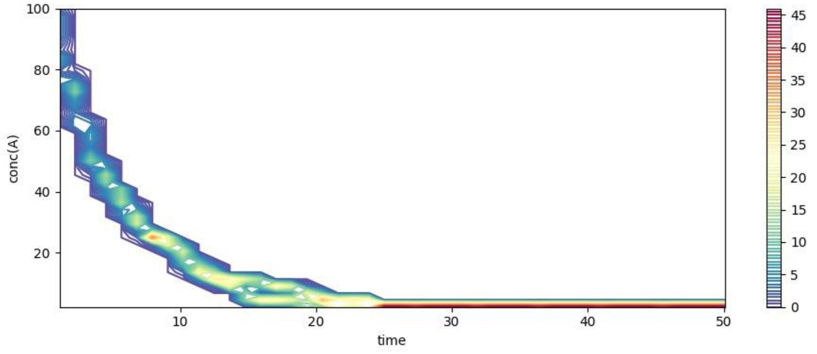

12.1.2 Parameter estimation (A => B => C)

In this example, we will use the files inside the “ParameterEstimation_examples” folder.

1. Open BioSSL as usual
2. cd to the directory or path of the “TutorialTopoFiles” folder downloaded from github. After the download, it will normally be in the downloads folder.
   cd %Userprofile%\Downloads\TutorialTopoFiles
   cd ParameterEstimation_examples

3. Type the following command:
   load param_est.ssl.txt;

4. The following output will appear:

```
Final result =
  final_simplex: (array([[0.5, 0.3],
       [0.5, 0.3],
       [0.5, 0.3]]), array([5.18947686e-18, 8.19852494e-18, 2.62531235e-17]))
           fun: 5.189476861565852 4e-18
       message: 'Optimization terminated successfully.'
          nfev: 139
           nit: 71
        status: 0
       success: True
             x: array([0.5, 0.3])



kf1 = 0.5000000000154016

kf2 = 0.30000000000837307
```

The contents of “param_est.ssl.txt” are as follows:

propagate A => B, -1 & B => C, -1 where A=100 & B=0 & C=0 using k_est6 **with** EdataFile=AtoBtoC_data.txt**;**

It tells BioSANS that the rate constant in reaction **A => B** is unknown as well as **B => C** and the initial amount of A is 100, for B and C are 0. The method used here is **k_est6,** which pertains to the Nelder-Mead algorithm, Macroscopic, and the experimental data or expected trajectory is in **AtoBtoC_data.txt** file.

12.1.3 Symbolic computation

Type the following command and press enter:

```
propagate A => B, 1 & B => C, 1 where A=100 & B=0 & C=0 using Analyt;
```

The method "Analyt" performs pure symbolic computation. See section 6.2.1 for a complete list of symbolic keywords. The output of this run can be seen in the terminal window.

```
> propagate A => B, 1 & B => C, 1 where A=100 & B=0 & C=0 using Analyt;


Complex Analytical expressions


The complex expression is because sympy do not know how you want to simplify th
e expression

1

 A(t)   =   Ao*exp(-kf1*t)


 B(t)   =   (-Ao*kf1*exp(kf2*t) + (Ao*kf1 + Bo*(kf1 - kf2))*exp(kf1*t))*exp(-t*(k
f1 + kf2))/(kf1 - kf2)


 C(t)   =   (Ao*kf2*exp(kf2*t) + (kf1 - kf2)*(Ao + Bo + Co)*exp(t*(kf1 + kf2)) -
(Ao*kf1 + Bo*(kf1 - kf2))*exp(kf1*t))*exp(-t*(kf1 + kf2))/(kf1 - kf2)
```

12.1.4 Propagation from topology file

This time we will be working on the "TutorialTopoFiles" folder. First cd to that directory. If the current directory is "ParameterEstimation_examples" just issue the following command:

```
cd ..;
```

To use topology files, add a topo="topology file" after the "with" keyword. The following example makes use of "AtoB.dat" as the topology file. Even if we have the details in the topology, we still need to put the using <method> and with <options> in full detail. Run the following command:

```
propagate where A=100 & B=0 using ODE-2 with topo=AtoB.dat & fileUnit=molar;
```

Check the current working directory folder, which is "TutorialTopoFiles". There are new files created in the directory.

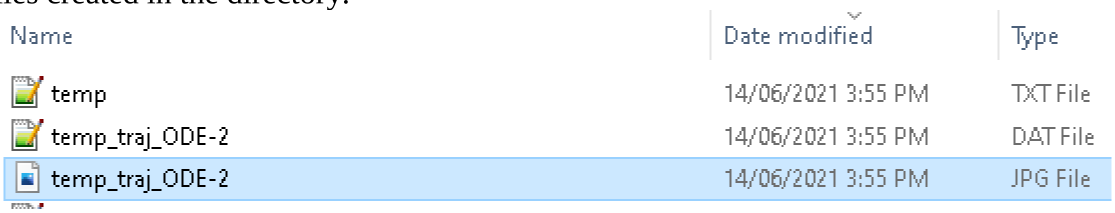

The temp folder contains some incomplete toplogy, which is not used because we have topo in the "with" keyword. The trajectory is in temp_traj_ODE-2.dat file. If we put an fout="something", the name of our trajectory will be that something. The following image is the plot of the trajectory:

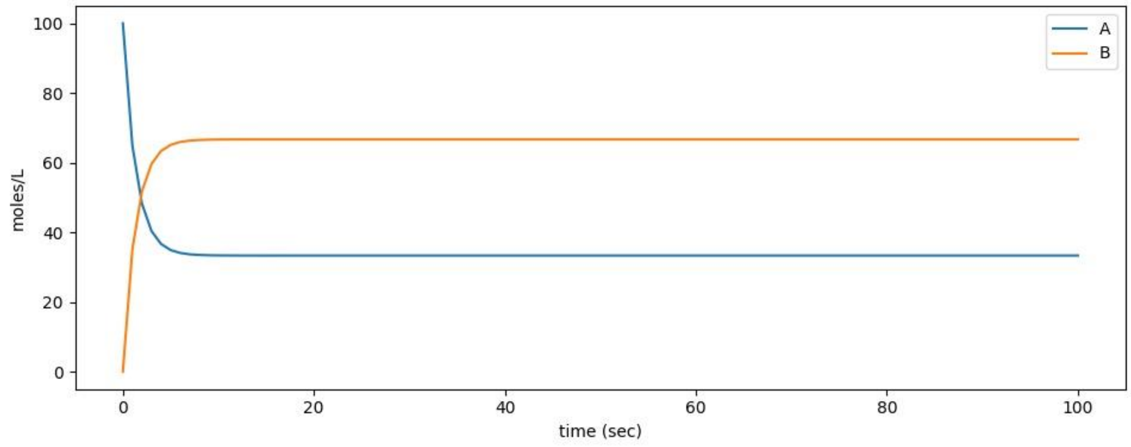

12.1.5 Automated simulations in one file

We can perform automated simulation by just typing all our commands in one file and then loading that file in BioSSL. For example, type the following commands in a text file:

```
propagate A <=> B, 0.1, 0.2 where A=100 & B=0 using CLE with tn=50 & tlen=1000 & miter=30 &
mult_proc=True & fout=SSL_AtoB;
pdread_traj SSL_AtoB_CLE.dat as data;
print data;
plot data time A B;
```

Save it into a file "MySSL.txt" then load the file in BioSSL with

```
load MySSL.txt;
```

We can also perform propagation of many systems in one file

12.2 Using BioSANS as a Python import

The details of how to use BioSANS as a Python import were discussed in section 6.2. Several examples are provided in the following GitHub repository:

https://github.com/efajiculay/BioSANS_installers/tree/main/BioSANS_as_python_library

and the following GitHub page;

https://efajiculay.github.io/SysBioSoft/

REFERENCES

1. Alon U. An Introduction to Systems Biology: Design Principles of Biological Circuits. 1st edition. Boca Raton, FL: Chapman and Hall/CRC; 2006.

2. Monod J, Wyman J, Changeux J-P. On the nature of allosteric transitions: A plausible model. Journal of Molecular Biology. 1965;12: 88–118. doi:10.1016/S0022-2836(65)80285-6

3. Hucka M, Bergmann F, Hoops S, Keating SM, Novère NL, Myers CJ, et al. Systems Biology Markup Language (SBML) Level 3Core. : 173.

4. Oliveira SMD, Chandraseelan JG, Häkkinen A, Goncalves NSM, Yli-Harja O, Startceva S, et al. Single-cell kinetics of a repressilator when implemented in a single-copy plasmid. Mol BioSyst. 2015;11: 1939–1945. doi:10.1039/C5MB00012B

5. Lorenz EN. Deterministic Nonperiodic Flow. Journal of the Atmospheric Sciences. 1963;20: 130–141. doi:10.1175/1520-0469(1963)020<0130:DNF>2.0.CO;2

6. Putzer EJ. Avoiding the Jordan Canonical Form in the Discussion of Linear Systems with Constant Coefficients. The American Mathematical Monthly. 1966;73: 2–7. doi:10.1080/00029890.1966.11970714

7. Moya-Cessa HM, Soto-Eguibar F. Differential equations: an operational approach. 2011.

8. Kampen NGV. Stochastic Processes in Physics and Chemistry. 3 edition. Amsterdam ; Boston: North Holland; 2007.

9. Elf J, Ehrenberg M. Fast Evaluation of Fluctuations in Biochemical Networks With the Linear Noise Approximation. Genome Res. 2003;13: 2475–2484. doi:10.1101/gr.1196503

10. Okada T, Mochizuki A. Law of Localization in Chemical Reaction Networks. Phys Rev Lett. 2016;117: 048101. doi:10.1103/PhysRevLett.117.048101

11. Press WH, Teukolsky SA. Adaptive Stepsize Runge-Kutta Integration. Comput Phys. 1992;6: 188. doi:10.1063/1.4823060

12. Ross S. Chapter 12 - Markov Chain Monte Carlo Methods. In: Ross S, editor. Simulation (Fifth Edition). Academic Press; 2013. pp. 271–302. doi:10.1016/B978-0-12-415825-2.00012-7

13. Theodoridis S. Chapter 12 - Bayesian Learning: Inference and the EM Algorithm. In: Theodoridis S, editor. Machine Learning. Oxford: Academic Press; 2015. pp. 585–638. doi:10.1016/B978-0-12-801522-3.00012-4

14. Do CB, Batzoglou S. What is the expectation maximization algorithm? Nature Biotechnology. 2008;26: 897–899. doi:10.1038/nbt1406

15. Gao F, Han L. Implementing the Nelder-Mead simplex algorithm with adaptive parameters. Comput Optim Appl. 2012;51: 259–277. doi:10.1007/s10589-010-9329-3

16. Storn R, Price K. Differential Evolution – A Simple and Efficient Heuristic for global Optimization over Continuous Spaces. Journal of Global Optimization. 1997;11: 341–359. doi:10.1023/A:1008202821328

17. Powell MJD. An efficient method for finding the minimum of a function of several variables without calculating derivatives. The Computer Journal. 1964;7: 155–162. doi:10.1093/comjnl/7.2.155

18. Zhu C, Byrd RH, Lu P, Nocedal J. Algorithm 778: L-BFGS-B: Fortran subroutines for large-scale bound-constrained optimization. ACM Trans Math Softw. 1997;23: 550–560. doi:10.1145/279232.279236

19. Gillespie DT. The chemical Langevin equation. The Journal of Chemical Physics. 2000;113: 297–306. doi:10.1063/1.481811

20. Cao Y, Gillespie DT, Petzold LR. Efficient step size selection for the tau-leaping simulation method. The Journal of Chemical Physics. 2006;124: 044109. doi:10.1063/1.2159468

21. Gillespie DT. Exact stochastic simulation of coupled chemical reactions. J Phys Chem. 1977;81: 2340–2361. doi:10.1021/j100540a008
